# Social and Health Conditions That Drive the Need and Demand for, Use of and Expenditure on Social Care in the UK: A Systematic Review

**DOI:** 10.1002/cesm.70093

**Published:** 2026-08-12

**Authors:** Anna Mae Scott, Ben Amies‐Cull, Emre Oguzman, James Webster, Rhys Llewellyn Thomas, Anne Mason, Laura Bojke, Matthew Quinn, Paul Clarkson, Joe Spearing, Sasha Shepperd

**Affiliations:** ^1^ Warwick Medical School University of Warwick Coventry UK; ^2^ Nuffield Department of Primary Care Health Sciences University of Oxford Oxford UK; ^3^ Nuffield Department of Population Health University of Oxford Oxford UK; ^4^ Centre for Health Economics University of York York UK; ^5^ Division of Nursing University of Manchester Manchester UK

**Keywords:** inequalities, social care

## Abstract

**Background:**

The need for social care, in the form of practical assistance and personal care, is increasing alongside a growing older population with long‐term conditions and places those with limited or no access to publicly funded care at risk of increasing levels of unmet need.

**Aim:**

This systematic review sought to understand the role of social and health conditions in the need, demand, utilization, and expenditure on social care in the United Kingdom.

**Methods:**

We searched Medline, CINAHL, EconLit, ASSIA, and the Campbell Collaboration from January 1, 2009 to April 14, 2025, and gray literature on August 21, 2024, for randomized trials, cohort studies, case control studies, and interrupted time series, cross‐sectional, and modeling studies of populations aged ≥ 60 years old, in a UK setting, that examined the association of social and health conditions with the need and demand for, use of and expenditure on social care. We conducted risk of bias assessments using ROBINS‐E for longitudinal studies, and intended to use Risk of Bias 2 for randomized trials. We applied GRADE to assess the certainty of evidence. Results were synthesized narratively, and presented in an Evidence Gap Map.

**Results:**

We included 10 longitudinal cohort and eight cross sectional studies, study sample size ranged from 526 to > 400,000 participants. Five of the cohort studies were assessed as high risk of bias, and the cross sectional studies as moderate concern or high risk of bias. Older age was associated with increased unmet need for care in most studies, and living alone with increased unmet need for care and use of residential care. Results for ethnicity, deprivation and sex varied across studies, with studies using different measures of deprivation that limited comparability of findings. A range of long‐term health conditions were assessed, some studies indicated an association with an increased need and use of care; there were mixed findings for cost. For populations with cognitive impairment or dementia, the association with age and the use and cost of care varied, there was no clear association for sex, ethnicity and living alone; a previous hospital admission or ongoing health problems that included severity of dementia were associated with increased use of care.

**Conclusion:**

Our review of UK evidence indicates that there is insufficient evidence to accurately identify populations age > 60 years that are at increasing risk for needing care due to their social and health conditions.

## Summary

- What is the impact of social conditions (in parallel to health conditions, or alone) on the need and demand for, use of and expenditure of social care?
- **What did we do?**
- We followed standard systematic review methods to identify and synthesize evidence on the association of social and health conditions with the need and demand for and use and cost of care, in UK populations aged 60 years and older.
- **What did we find?**
- We identified 18 studies, with sample sizes between 526 to > 400,000. We summarized the evidence narratively as studies used different sources of data and methods.
- **Main results**
- Older age and living alone, and for some studies long‐term conditions, were associated with increased need for care, and living alone and long‐term conditions with increased use of residential care. Results for ethnicity, deprivation and sex varied across studies. For populations with cognitive impairment or dementia, the association with age and the use and cost of care varied, there was no clear association for sex, ethnicity and living alone, and a previous hospital admission or ongoing health problems were associated with increased use of care.
- **Authors conclusions**
- There is low to very low certainty of evidence on the impact of social and health conditions on the need and use of social care in later life.

## 1 Introduction

Efforts to optimize independent living in later life are challenged by an increase in the number of older people living with complex health and care needs due to long‐term conditions (LTC) that disproportionately affect those who are most deprived [1, 2, 3], demographic changes such as ageing without children [4], and limited public funding [5]. When social and economic determinants of health affect the conditions that people live in, such as inadequate housing or living alone, they can have a lasting influence on the trajectory of care [6, 7, 8].

The provision of funded social care, in the form of practical assistance and personal care to support the well‐being, independence and dignity of older populations varies across countries [5, 9]. While care can be defined by specific tasks, such as meal preparation and assistance with dressing and washing, the concepts underpinning the need and demand for care lack clear definition. In part this can be explained by the relative nature of need for care [10], and the overlap between health and care needs [11]. Setting eligibility criteria to define entitlement to services, used in the UK, provides some indication of need but misses those whose needs are not formally assessed. Similarly, defining the demand for care is not straightforward as relying on requests for paid care will miss the demand for care from informal networks of unpaid carers, from those who do not meet a set threshold for eligibility or have limited access to formal arrangements for assessing eligibility. Accurate measurement of the demand for care is further complicated by a lack of available data if there are multiple providers of care within a given system of health and care [12, 13].

Previous reviews have focused on specific populations, the concepts of need and demand for care [14, 15, 16] or levels of government expenditure on social care and its impact on unpaid carers [17]. To the best of our knowledge, evidence on the need, demand, use and expenditure on social care in an older population in the context of the conditions people live in has not been systematically reviewed.

To understand the extent that social conditions (in parallel to health conditions or alone) drive the need and demand for, use of and expenditure on care we conducted a systematic review to identify, appraise and summarize the evidence from individual studies in a UK context.

## 2 Methods

### 2.1 Protocol Development and Registration

The review protocol is registered on the Open Science Framework (osf.io/rpyd2), and available on medRxiv [18]. Changes to the review protocol are described in Appendix S1.

### 2.2 Criteria for Considering Studies for This Review

#### 2.2.1 Research Question

What is the impact of social conditions (in parallel to health conditions, or alone) on the need and demand for, use of and expenditure of social care?

#### 2.2.2 Population

We included studies of adults aged ≥ 60 years old who lived in any setting and who were assessed as needing social care. We included studies conducted in one or more of England, Scotland, Wales, and Northern Ireland.

#### 2.2.3 Exposures

We included studies that examined the association of social conditions, that is, the circumstances that affect people's lives, in parallel to health conditions such as dementia, on the need and demand for, use of and expenditure on social care in the UK. These have been described as the “causes of the causes,” that is, factors that are beyond the immediate cause of poor health and the need for care [19]. Social conditions of interest included, but were not limited to, socio‐economic (e.g., education, deprivation), environmental (e.g., housing conditions), social and community networks (e.g., living alone) and individual (e.g., age, sex) factors that can influence an individual's health and need for care in later life [6, 20, 21, 22, 23].

#### 2.2.4 Outcomes

We defined the outcome need for care as requiring “personal care and support due to needs arising from age, illness, disability or other circumstances,” [8] usually measured by limitations to activities of daily living and/or mobility difficulties, and included evidence for unmet and met need, as described by study authors. Demand was defined as requests for funded or unfunded care; use as receiving paid or unpaid care; and, expenditure as the amount spent on care or the cost of care.

#### 2.2.5 Types of Studies

We aimed to include randomized trials, follow‐up studies of any design, including but not restricted to prospective cohort studies, case‐control studies and interrupted time series, cross sectional studies (single time point) and simulation studies. Qualitative studies were excluded.

### 2.3 Search Methods for Identification of Studies

We designed a search strategy for Medline to identify drivers, predictors or determinants of need and demand for, use of and expenditure of social care. We translated this from Medline to other databases using the Polyglot Search Translator https://www.sr-accelerator.com/#/polyglot and tested it against a validation set of articles. The search strategy was designed by AMS and reviewed by an information specialist (NR).

We searched the following databases on June 13, 2024, and updated the search on April 14, 2025:

1. PubMed
2. CINAHL (via Ovid)
3. EconLit (via Ovid)
4. Applied Social Science Index and Abstracts, ASSIA (via ProQuest)
5. The Campbell Collaboration (via www.campbellcollaboration.org)

We restricted the searches to articles published in English, and from January 1st, 2009 due to changes in the provision of social care that occurred at this time [24]. For the searches in Medline, CINAHL and EconLit, an adapted version of the NICE UK geographic filter was used [25]; and for ASSIA, the database's filter for UK, England, Wales, Scotland or Northern Ireland. No geographic filter was used for the Campbell systematic review database. We conducted a forward and backward citation search using the SpiderCite tool (https://sr-accelerator.com/#/spidercite) on October 29, 2024. We searched the websites in August 21st, 2024 for NHS England, the Department of Health and Social Care, the National Service Scotland, NHS Wales, Health and Social Care Northern Ireland, the Social Care Institute for Excellence, the National Institute for Health and Care Excellence, and the National Institutes for Health and Care Research, the Nuffield Trust, the King's Fund and The Health Foundation; and the Overton Knowledge Database for unpublished and policy documents. Full details of the searches are provided in Appendix S2.

### 2.4 Data Collection and Analysis

#### 2.4.1 Screening Studies for Inclusion

After de‐duplication we used Covidence for screening articles for inclusion [26], one reviewer (AMS) screened all titles and abstracts for inclusion/exclusion, and two reviewers (out of AMS, SS, MQ, BAC) independently assessed the full text of studies assessed as eligible. Disputes or uncertainties were resolved by discussion with AM and LB.

#### 2.4.2 Data Extraction

The data extraction form was designed by the authors and piloted on three of the included studies, data extraction was completed by AMS and SS. Data extraction included:

1. Study characteristics: reference, study location, study design, study duration, data source.
2. Participant/population characteristics: number, age, gender, ethnicity, other PRO‐EDI characteristics [27], and health conditions.
3. Concept/exposure: social conditions in parallel to health conditions or alone that are known to increase the risk of long‐term need for care, such as demographics, living alone, dementia or multiple long‐term condition (MLCT) [20].
4. Outcomes: need, as measured by the study authors, demand for or use of and expenditure on formal (e.g., home, residential/nursing care) or informal care.
5. Setting, for example, community, nursing or residential care homes and geographic location.

#### 2.4.3 Risk of Bias Assessment

Following guidance, two reviewers out of AMS, JW, BAC, EO, SS independently assessed risk of bias for the longitudinal cohort studies using the seven ROBINS‐E risk of bias domains: D1 (domain 1) confounding, D2 measurement of the exposure, D3 selection of participants into the study or analysis, D4 post‐exposure interventions, D5 missing data, D6 measurement of the outcome, and D7 selection of the reported result. Each domain was rated as low risk of bias, some concerns, or high risk of bias. We provide a rating of an overall risk of bias, which matches the rating for the domain with the greatest risk of bias [28]. We limited our appraisal of cross‐sectional studies to (1) confounding, (2) measurement of social and health conditions (exposures) and (3) measurement of the outcomes; and rated each domain as low risk, some concerns or high risk of bias as consensus on the key concepts of bias for this study design is lacking [29].

#### 2.4.4 Data Synthesis

We had planned to calculate the effect size using meta‐analysis for each social or health condition with outcome data for ≥ 3 comparable studies. If sufficient data were available, we planned to analyze the findings by specific health condition (e.g., diabetes, dementia, etc.) of the study population or combination of health conditions. Due to heterogeneity in the range and measurement of conditions and study designs we report the results narratively, using the SWiM guidance [30]. We grouped results by the specific social and/or health conditions: age, sex and ethnicity; living alone; deprivation; long‐term conditions; and diagnosis of dementia or cognitive impairment.

Two authors (SS, AMS) assessed the certainty of the evidence for each outcome by applying the five GRADE domains that lower certainty: risk of bias, inconsistency, indirectness, imprecision and publication bias; and three which may increase certainty: a large effect, a dose response gradient and/or when there is plausible confounding that might reinforce the inference of effect. The certainty of evidence for each outcome was rated as very low (the true effect is probably markedly different from the estimated effect), low (the true effect might be markedly different from the estimated effect), moderate (the authors believe that the true effect is probably close to the estimated effect) or high (the authors have a lot of confidence that the true effect is similar to the estimated effect) [31, 32, 33].

#### 2.4.5 The Role of the Funder

The funder had no role in the design, analysis or reporting the results of this systematic review.

## 3 Results

### 3.1 Search Results

**FIGURE 1:**
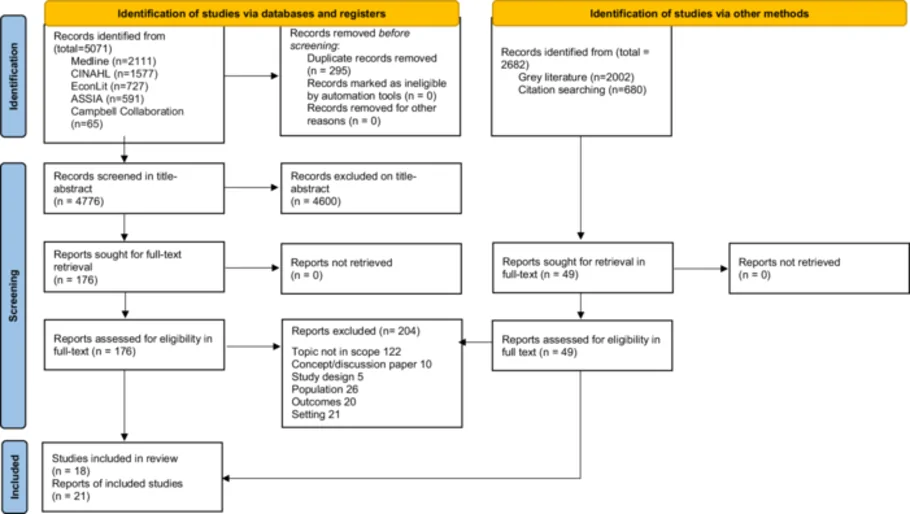
PRISMA flow diagram.

Searches yielded 7753 references, of these 225 full‐text publications were assessed for inclusion. Eighteen studies published in 21 reports were included [34, 35, 36, 37, 38, 39, 40, 41, 42, 43, 44, 45, 46, 47, 48, 49, 50, 51, 52, 53, 54]; no eligible studies were identified from the search of websites (Figure 1). Reasons for excluding studies are provided in Appendix S3.

### 3.2 Included Studies

The 18 studies included 10 longitudinal cohorts (reported in 13 publications) with follow‐up from 6 months to 10 years [35, 36, 37, 38, 40, 41, 42, 43, 45, 47, 48, 50, 52], and eight cross‐sectional studies [34, 39, 44, 46, 49, 51, 53, 54]. The longitudinal cohorts included one randomized trial that combined randomized groups and two secondary analyses of electronic health data. Fourteen studies were set in England [34, 36, 38, 39, 43, 44, 45, 46, 47, 50, 51, 52, 53, 54], one in Scotland [35], one in Wales [49], and two in England, Scotland and Wales [41, 48]. Five studies used linked health and social care data [35, 46, 47, 50, 52].

**FIGURE 2:**
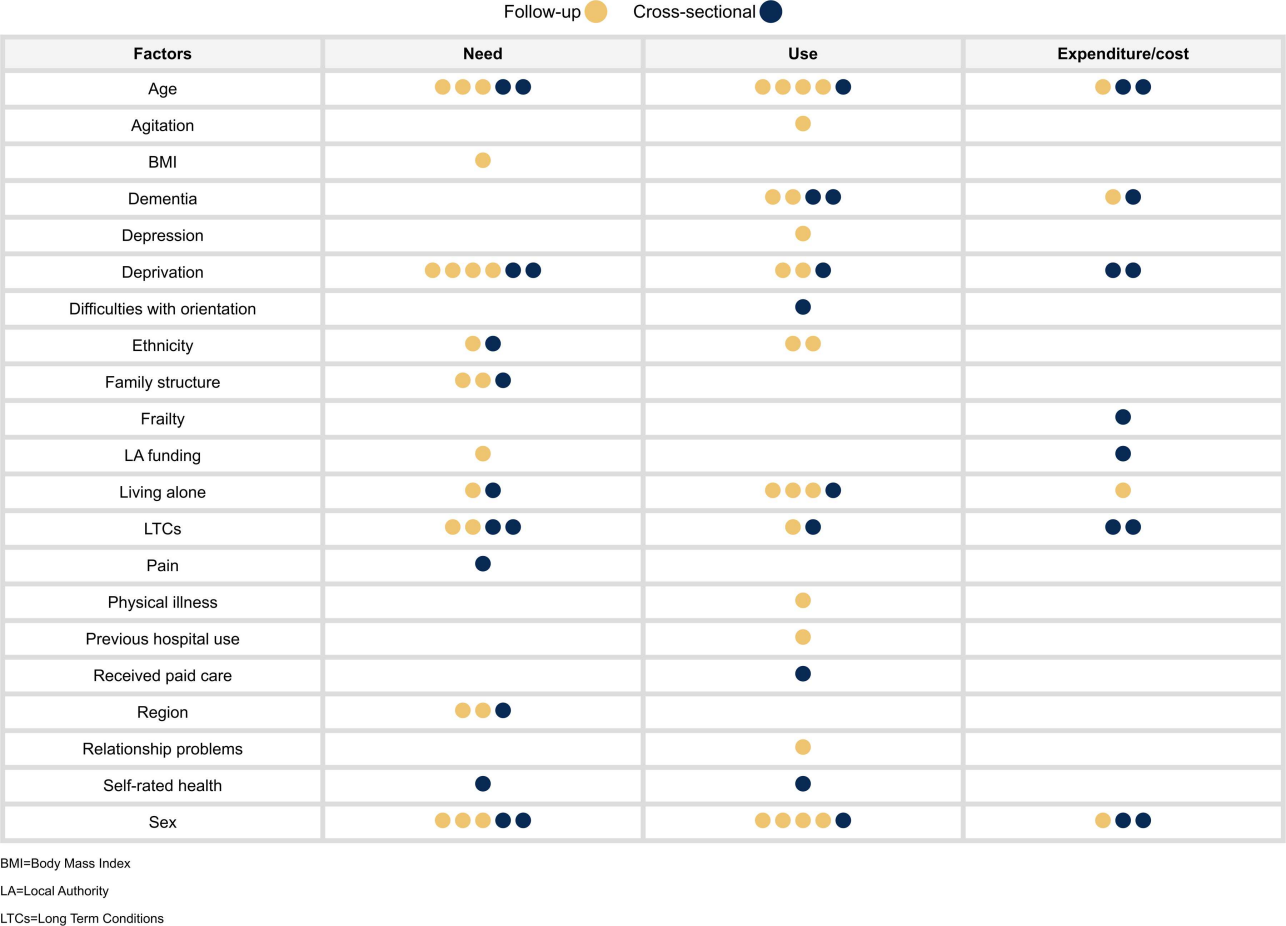
Social conditions associated with the need for, use of, cost and expenditure of social care.

Study populations included those at risk of cardiovascular disease, average age 76 years [35]; three or more long‐term conditions, average age 78 years [46]; MLTC, aged ≥ 65 years [34]; post‐elective hip or knee surgery (sample eligible for this review aged ≥ 65) [52]; the Newcastle cohort ≥ 85 years [36]; a cohort aged > 75 years [50]; populations aged between 60 to 75 years sampled from the English Longitudinal Study of Ageing (ELSA) (+ /− another data source) [38, 39, 43, 44, 45, 51, 53, 54]; cognitive impairment, aged ≥ 65 years [49]; a diagnosis of Alzheimer's Disease, 98.4% ≥ 60 years [47]; and mild or moderate cognitive impairment or a diagnosis of dementia, average age > 75 years [40, 48]. Among the PRO‐EDI characteristics, age and location were reported by all 18 studies, ethnicity by 6/18 [39, 43, 47, 49, 50, 52], sex by 17/18 [34, 35, 36, 38, 39, 41, 43, 44, 46, 47, 48, 49, 50, 51, 52, 53, 54], education level by 8/18 studies [36, 38, 39, 41, 43, 44, 51, 53], and disability by 16/18 [35, 36, 38, 39, 41, 44, 45, 46, 47, 48, 49, 50, 51, 52, 53, 54]. Details of the characteristics of the study populations and sources of data are reported in Tables S1 and S2. To illustrate the breadth of social and health conditions measured by the included studies, we created an evidence gap map [55] using the D3 library in JavaScript (Figure 2).

The need for care was measured by the number of difficulties with activities of daily living (ADLs), instrumental activities of daily living (IADLs) or mobility difficulties, with studies using different thresholds for the number of difficulties to determine need [36, 38, 39, 45, 53]. One study classified participants into trajectories of low, medium or high long‐term need for care (defined by the number of functional limitations) and low, medium or high long‐term intensity care (defined by the hours of formal and informal care received) [43]. The demand for care was not reported. Ten studies reported the use of care [35, 42, 43, 44, 47, 48, 49, 50, 52, 54], three reported unpaid care [43, 44, 54]. Five studies reported the cost of and/or expenditure on care [34, 41, 46, 47, 51].

### 3.3 Risk of Bias of Included Studies

**FIGURE 3:**
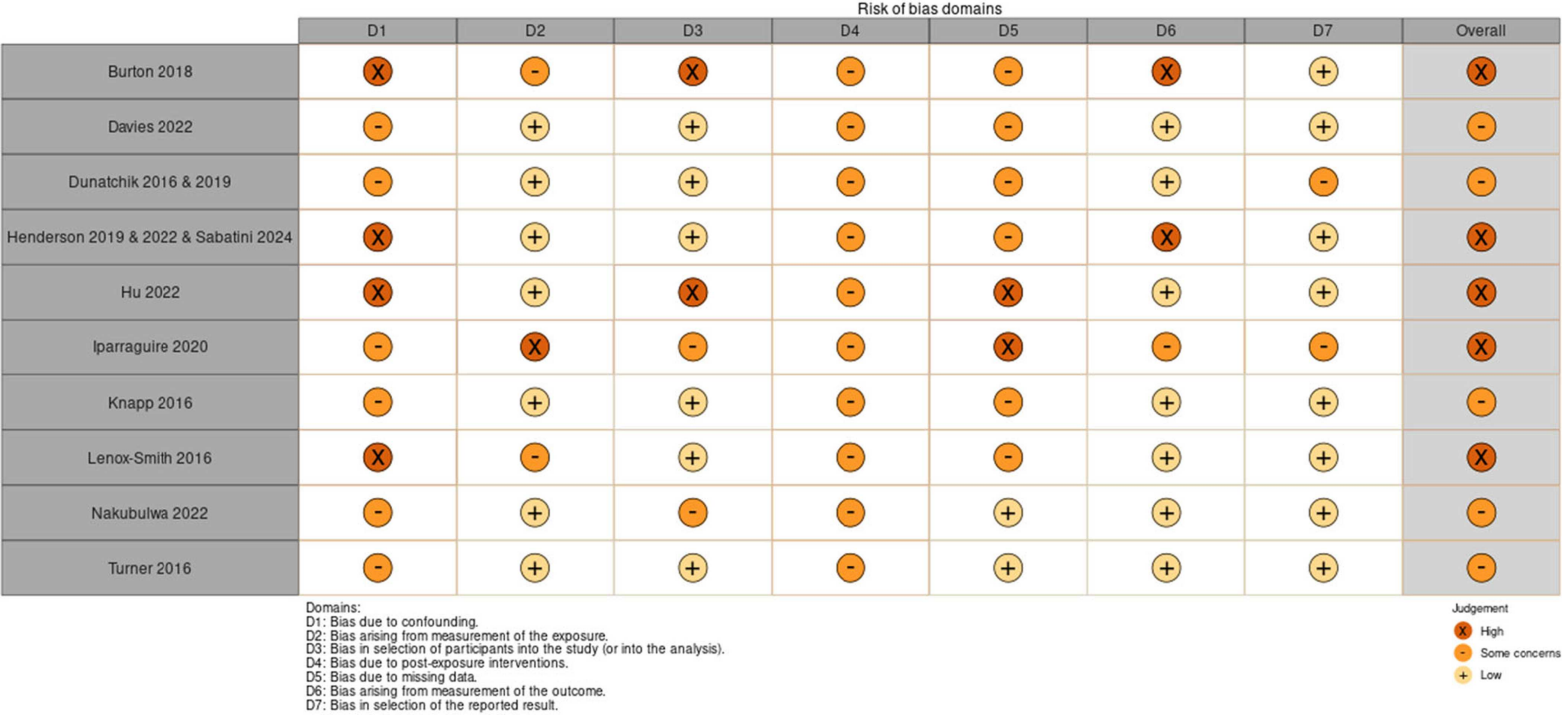
ROBINS‐E risk of bias ratings for the longitudinal cohort studies.

The risk of bias for the longitudinal cohort studies is summarized in Figure 3. The main sources of bias were residual confounding, a lack of information on post‐exposure interventions in all studies, measurement of outcomes and missing data. For the cross‐sectional studies, the majority of studies were assessed as some concerns about the risk of bias for residual confounding, measurement of the exposures and outcomes (Table 1).

**TABLE 1:** Risk of bias ratings for cross‐sectional studies.

Study	Confounding	Measurement of exposure	Measurement of outcome
Chukwusa 2023	Some concerns	Low	Some concerns
Gousia 2021	Some concerns	Some concerns	Some concerns
Hu 2024 [Ageing & Soc]	Some concerns	Some concerns	Some concerns
Kasteridis 2015	High	Some concerns	Some concerns
MacLeod 2021	Some concerns	High	High
Nikolova 2022	Some concerns	Some concerns	Some concerns
Vlachantoni 2015	Some concerns	Some concerns	Some concerns
Vlachantoni 2019	Some concerns	Some concerns	Some concerns

### 3.4 Effect of Social and Health Conditions

We report the findings by the most frequently reported social and health conditions associated with the need for, use and cost of and expenditure on social care in an older population; we have reported the results for sub‐groups if these are a closer match to our inclusion criteria than the overall sample (e.g., by age). Full details of study results are reported in Table S3, and an assessment of the certainty of the evidence is presented in the Summary of Findings (Table S4).

#### 3.4.1 Age, Sex and Ethnicity

##### 3.4.1.1 Need for, Use and Cost of Care

Eight studies assessed age, with most finding an association with increasing age and the need for care, mixed results for the use of care and little evidence on cost *(very low certainty evidence for need and cost; low certainty for use of care)*. Increasing age in a population aged > 65 years was associated with being more likely to have unmet need in one cohort (ELSA data) (OR 1.03, 95% CI 0.88–1.01, *N* = 4058, 8‐year follow‐up) [45] and two cross‐sectional studies (range: OR 1.01, 95% CI 1.00–1.02, *N* = 81,338, to OR 3.00, 95% CI 1.8–4.99, *N* = 5182) [39, 53]. As age increased, one study that analyzed ELSA data found unmet need for care was less likely (range OR 0.70, 95% CI 0.36–1.36 age 65–69 years, to OR 0.24, 95% CI 0.12–0.47 age 75+; *N* = 1082, 10‐year follow‐up); this was explained by being more likely to receive formal paid care as people age [38]. One longitudinal cohort (the Newcastle 85+ cohort) of an older population aged > 85 years found no clear association with age and the need for care (HR 1.02, 95% CI 0.96–1.09; *N* = 845, 10 year follow‐up) [36]. For the use of care, age was associated with a higher risk of admission to a long‐term care facility in one randomized trial which combined randomized groups (HR 1.06 per year, 95% CI 1.03–1.09, *N* = 2,033, 3‐year follow‐up) [35], and accessing all types of long‐term care arranged and fully or partly funded by a local government in one retrospective longitudinal cohort study (OR 2.17, 95% CI 1.55–3.04, *N* = 13,394; 12 months follow‐up) [50]. A cross‐sectional study reported no clear association for age and social care costs in a population with ≥ 3 long‐term conditions [46].

Ten studies assessed sex with mixed results *(very low certainty evidence)*. One longitudinal cohort study (the Newcastle 85+ cohort) found that females had a higher risk of mobility disability (need) (HR 1.64, 95% CI 1.25–2.14; *N* = 845, 10‐year follow‐up) [36], and two cross‐sectional studies that males were more likely to have unmet need (OR 1.64, 95% CI 1.44–1.87, *N* = 81,338; OR 1.47, 95% CI 1.07–2.02 and OR 2.00, 95% CI 1.49–2.67, *N* = 5591 for different measures of need) [39, 53]. Two longitudinal cohort studies, both used ELSA data, reported no clear association with sex and unmet need (OR 1.35, 95% CI 0.93–1.95, *N* = 1082, 10 years follow‐up; OR 1.003, 95% CI 0.895–1.123, *N* = 4058, 8 years follow‐up). [38, 45]. For trajectories of need and use of care, one longitudinal cohort study (ELSA data) found that females were more likely to have a medium‐need/medium intensity trajectory versus low‐need/low‐intensity trajectory of care (OR 1.35, SE 0.1, *p* < 0.001; *N* = 4629, 6 year follow up), indicating more need and/or use of care with needs defined by the number of functional limitations and intensity by the weekly hours of care [43]. For use of care, in one longitudinal cohort that combined randomized groups males were less likely to have a long‐term care admission (HR 0.72, 95% CI 0.53–0.99, *N* = 2033; 3 year follow‐up), [35] no clear association was found by one retrospective cohort for male/female and the use of all types of long‐term care arranged by a local authority (OR 1.25, 95% CI 0.94–1.67, *N* = 13,394, 12 months follow‐up) [50], and a cross‐sectional study found that females were more likely to use paid care (OR 1.63, 95% CI 1.08–2.47, *N* = 3395) [54]. For cost, a cross‐sectional study of a population with ≥ 3 long‐term conditions found that compared with females, males were more likely to have lower social care costs (‐37%, *N* = 96,005; 2012/2013 GBP) [46].

Three studies assessed ethnicity with no clear association *(very low certainty evidence)*. For the need for care, one cross‐sectional study assessed the association of self‐reported ethnic background (ref white, OR 1.15, 95% CI 0.83–1.59, *N* = 81,338) [39]. Ethnicity was associated with being more likely to follow a medium‐need/medium intensity trajectory of care versus a low‐need/low intensity trajectory in one longitudinal cohort study (ELSA data) (ref: white OR 2.02, SE 0.47, *p* < 0.1; *N* = 4629, 6‐year follow‐up), indicating a trajectory of more need and/or use of care [43]. For use of care, a retrospective cohort study found no clear association by ethnic group (ref white; Asian/British Asian OR 1.28, 95% CI 0.77–2.03; Black/Black British OR 1.31, 95% CI 0.79–2.10; Mixed 1.16, 95% CI 0.77–1.72; *N* = 13,394, 12 months follow‐up) [50].

#### 3.4.2 Living Alone

##### 3.4.2.1 Need for, Use and Cost of Care

Five studies assessed living alone with some evidence that living alone is associated with being more likely to have unmet need for care *(low certainty evidence for need, very low certainty for use)*. A longitudinal cohort (ELSA data) and a cross‐sectional study found that living alone increased the likelihood of having unmet need (OR 1.74; 95% CI 1.08–2.80; *N* = 1082, 10 years follow‐up [38]; OR 3.30, 95% CI 1.71–6.37 for help with ADLs, OR 1.93, 95% CI 1.09–3.41 for help with mobility) [53]. For trajectories of need and use of care, married versus single people were more likely to have high‐needs/high‐intensity use of care versus high‐needs/medium‐intensity trajectory (OR 3.57; SE 0.84, *p* < 0.001, *N* = 40,629, 6 years follow‐up), although marital status is a proxy for living alone results indicated that single individuals received fewer hours of care due to less unpaid informal care (one longitudinal cohort that used ELSA data) [43]. For use of care, there was an increased probability of discharge to a nursing home following a hip or knee replacement (0.60 percentage points, *p* < 0.001; *N* = 48,146, 6 months follow‐up) [52], and in one retrospective cohort study no clear association with living alone and the use of long‐term care (OR 1.35, 95% CI 0.69–2.98; *N* = 13,394, 12 months follow‐up) [50].

#### 3.4.3 Deprivation

##### 3.4.3.1 Need for, Use of and Cost of Care

Eight studies assessed deprivation, measured by level of education, income, wealth, housing conditions, occupation and area deprivation with mixed results *(very low certainty evidence)*. For studies that measured income and wealth, we report the findings for wealth rather than income due the age of the population. For education, two longitudinal cohort studies (the Newcastle 85+ cohort; and ELSA data), each with 10‐year follow‐up, found no clear association with education and the need for care (years of education HR 0.97, 95% CI 0.82–1.14, *N* = 845; no education vs. higher education OR 1.05, 95% CI 0.57–1.93, *N* = 1082) [36, 38], a cross‐sectional study that unmet need for care was less likely for those with higher levels of education (school qualifications at age 16 years and education at age ≥ 18 years vs. no school qualifications, OR 0.77, 95% CI 0.66–0.91; OR 0.58, 95% CI 0.49–0.69, *N* = 81,338) [39] For wealth, one longitudinal cohort study that used ELSA data reported no clear association with unmet need (OR 1.00, 95% CI 1.00–1.00, *N* = 4058, 8‐year follow‐up) [45], and a cross‐sectional study that unmet need was less likely with increased levels of non‐housing wealth (ref: 1st quintile, range 2nd quintile OR 0.74, 95% CI 0.65–0.85 to 5th quintile OR 0.41, 95% CI 0.34–0.50, *N* = 81,338) [39]. One longitudinal cohort study that used ELSA data found that multiple housing problems such as shortage of space, dampness, low temperatures during the winter, were associated with being more likely to follow a medium‐need/medium intensity trajectory of care versus a low‐need/low intensity trajectory (OR 1.71 (SE 0.05), *p* < 0.001; *N* = 4269, 6 year follow‐up), indicating a trajectory of more need and use of care; and a higher level of wealth and higher education were associated with being less likely to be in a medium intensity need and use of care versus lower intensity need and use of care trajectory (OR 0.76, SE 0.08, *p* < 0.05; OR 0.80 [SE 0.08] *p* < 0.05) [43]. For the use of care, in a cross‐sectional study, the findings from a wealth‐based concentration index (CI) found the use of formal and informal care was concentrated among the poor (CI −0.052, (SE 0.005), *p* < 0.001; CI −0.186 (SE 0.007), *p* < 0.001, *N* = 16,458) [44]; with similar findings from a retrospective longitudinal cohort study that found living in a less deprived area, defined by an Index of Multiple Deprivation (IMD), was associated with less use of local authority arranged and funded long term care (ref 1st quintile [most deprived], IMD 4th/5th quintile OR 0.37, 95% CI 0.23–0.56; *N* = 13,394, 12 months follow‐up) [50]. For cost, one cross‐sectional study reported no association with deprivation [46].

#### 3.4.4 Long‐Term Conditions (LTCs)

##### 3.4.4.1 Need for, Use of and Cost of Care

Eight studies assessed one or more LTCs, with mixed results *(very low certainty evidence)*. One of two longitudinal cohort studies (the Newcastle 85+ cohort), each with 10‐year follow‐up, found an additional LTC was associated with increased mobility disability (need for care) (HR 1.16, 95% CI 1.07–1.25; *N* = 845) in a population aged ≥ 85 years [36]; and the second study (ELSA data) that a LTC was associated with a reduction in unmet need for care (OR 0.56, 95% CI 0.39–0.81 *p* < 0.05, *N* = 1082), that might be explained by being connected to services due to poor health and/or having care needs provided by unpaid informal carers [38] Two cross‐sectional studies of ELSA data used different counts of difficulties with ADLs to measure need; in one there was an increase in unmet need for eight LTCs (range: OR 1.52, 95% CI 1.29–1.80 for diabetes to OR 2.69, 95% CI 2.37–3.07 for arthritis, *N* = 81,338) [39], in the second a reduction in unmet need with a long‐term illness for help with mobility (OR 0.59, 95% CI 0.39–0.89, *N* = 5591) [53]. For use of care, two out of seven LTCs were associated with increased use of long‐term care (a pre‐existing mental health condition OR 1.76, 95% CI 1.27–2.40 and a neurological condition OR 2.11, 95% CI 1.41–3.06; *N* = 13,394, 12 months follow‐up) in a retrospective longitudinal cohort study [50]. Three cross‐sectional studies measured the cost of care, a one percentage point increase in the prevalence of MLTC across England was associated with an increase of £8.13 (FY 2018/19) in per capita local authority expenditure on social care (death registry data across England, *N* = 409,551) [34]; annual savings on expenditure on formal social care, excluding care homes, were estimated as £4.4 million (inflated to 2018 value, *N* = 2408) for every 1% of a population ≥ 75 years who lived at home and did not develop frailty between 2012 and 2018 with frailty measured by the accumulation of deficits that included LTCs [51]; and in a population with 3 + LTC, there was no clear association with the number of LTC and social care costs (*N* = 1458; 2012/13 GBP) [46].

#### 3.4.5 Populations With a Diagnosis of Dementia or Cognitive Impairment

##### 3.4.5.1 The Use of and Cost of Care

Three studies measured a range of associations for the use and cost of care in populations with cognitive impairment or dementia *(low to very low certainty evidence)*. In a longitudinal cohort study of people with mild to moderate dementia (Improving the Experience of Dementia and Enhancing Active Life [IDEAL]), aged < 80 years was associated with a reduced risk of moving into a care home over 2 years (reported in 5‐year age bands), and Parkinson's disease dementia/dementia with Lewy bodies with an increased risk (RR 3.20, 95% CI 1.43–7.77; *N* = 851, 2 year follow‐up) [42]. There was no clear association for female/male and living in a care home (RR 0.65, 95% CI 0.34–1.19, *N* = 851, 2‐year follow‐up) [42]. In a population with Alzheimer's disease (AD) (the majority aged > 70), a longitudinal cohort study (secondary analysis of electronic health records (EHR) with 6 months follow‐up found that age was associated with care home admission (OR 1.04, 95% CI 1.02–1.06, *N* = 3075), no association for male (ref female) (OR 1.11, 95% CI 0.85–1.44, *N* = 3075), significant problems with living conditions (OR 1.65, 95% CI 1.18–2.32, *N* = 3075), admission to hospital (OR 1.54 95% CI 1.22–1.94, *N* = 3075) or a mental health facility (OR 2.59, 95% CI 1.42–4.74, *N* = 3075) in the previous twelve months, and agitation (OR 1.98, 95% CI 1.45–2.70, *N* = 3075); non‐white ethnicity was associated with a reduced risk (ref white, range OR 0.30, 95% CI 0.12–0.74 for mixed ethnicity to OR 0.57, 95% CI 0.38–0.86 for Caribbean/African, *N* = 3075), there was no association for physical illness, depression, or ethnicity and cost [47]. A prospective longitudinal cohort study of a population with probable AD found the severity of dementia increased the probability of being institutionalized at 18 months (mild dementia 9.0, 95% CI: 5.6–14.3; moderate severe/severe dementia 28.0, 95% CI: 21.0–36.7) [48], and a cross sectional study that cognitive impairment was associated with increased use of social care (OR 1.90, 95% CI 1.28–2.83; *N* = 3593) [49]. For cost, a longitudinal cohort (the IDEAL cohort) with 4 year follow‐up found that an additional year of age (sample mean 76 years) was associated with a 0.8% (ExpB 1.008, 95% CI 1.002–1.015, *N* = 846) increase in the rate of weekly costs of paid and unpaid care (2014/15 costs), no clear association was found for sex (female (ref male) ExpB 1.070, 95% CI 0.945–1.211, *N* = 846) [41]. Two longitudinal cohort studies (one secondary analysis of linked EHRs, and one the IDEAL cohort) with 6 month and 4 year follow‐up respectively, found that living alone was associated with increased cost from a care home admission [47], and for paid and unpaid care but the change in cost was not significantly different for those that lived alone and those who lived with others (ExpB 0.969, 95% CI 0.778, 1.208, *N* = 910) [41].

## 4 Discussion

We sought to understand the role of social and health conditions on the need, demand, utilization, and expenditure on social care in the United Kingdom. Eighteen studies eligible for inclusion assessed the association of more than 20 social and health conditions on the need for, use of and cost or expenditure of care. The majority of the studies reported on the need for or use of care, with need measured by different counts of activities of daily living, instrumental activities of daily living or mobility difficulties. Study sample size ranged from 526 (a longitudinal cohort study) to > 400,000 (ONS death registry data). Older age was associated with an increased need for or use of care, though for the older old population unmet need was less likely and might be explained by being more likely to receive formal paid care as people age. Living alone was associated with unmet need for care, receiving less unpaid care and increased use of residential care. Eight studies assessed the association of deprivation and care using different measures, there was some indication that increased levels of education and wealth were associated with a lower need for care and lower wealth was associated with the use of informal care. The findings for long‐term conditions varied due to the different LTCs measured, different definitions of need and study designs.

Compared with health [56], there is relatively little evidence of the direct impact of social conditions on the need and use of social care in the UK as people age. We found no studies that measured the demand for care. The evidence partially addressed a few of the major policy concerns. The increased likelihood of unmet need and use of paid care for those who lived alone has implications for the population that is ageing without children [57]; and although there was inconsistency in the findings for LTCs, a few studies indicated that the need for care was more likely in an older population with a LTC [3]. The evidence did not address the interdependency of health and social care that will continue to increase as the population ages [11]. How to measure the need for care is an area that has long been debated, with different conceptual models developed to frame need [10, 58]. A count of difficulties in ADLs, IADLs and mobility is the most frequently used measure; it aligns with formal assessments of need but does not reflect other domains such safety and social participation that promote independence [59]. In contrast to countries that operate social health insurance systems, the English care system does not explicitly define entitlement to care but there is a formal assessment to determine eligibility and ability to pay. This might limit the applicability of the evidence on the use of care to other settings.

The certainty of evidence was downgraded for risk of bias for all outcomes. A lack of data on post‐exposure interventions, for example a pharmacological or disease management intervention, that might have attenuated the need for care was a source of bias for all the included studies. Residual confounding and missing outcome data further lowered our certainty in the evidence. Inconsistency among findings for the majority of outcomes was due to heterogeneity in study design that included different sources of data, different populations and different categories of social conditions and measurements, different counts of daily and/or instrumental activities of living to measure the need for care and a high level of variability in cost data.

### 4.1 Limitations of the Review

Selecting studies for inclusion was not straightforward due to the complex study designs, a range of statistical techniques and a lack of clarity of terms that is not uncommon in complex reviews [60]. To address this, we designed a search strategy that focused on the main concepts of drivers, predictors and determinants. In addition, two reviewers independently selected studies for inclusion from a long list of full‐text articles and a third reviewer re‐assessed potentially eligible studies. We designed a broad search strategy to be over inclusive, but it is possible that we missed potentially eligible studies. A second issue was that it was not always possible to determine the chronological sequence of social and health conditions and the need for care, we dealt with this by using the term association instead of predictors [61, 62] but this provides limited understanding of the causal relationship between social conditions and the need for care.

We identified one systematic review of 46 studies conducted in Canada that assessed a range of social conditions associated with unmet need for primary care [63]. The large majority were cross‐sectional and similar to our review the findings were heterogeneous; low income and LTCs were associated with unmet need for primary care. While not directly related to the scope of our review, a systematic review of the predictors of nursing home placement for older people found inconsistent results for age, sex, education, income and some long‐term conditions and evidence that cognitive impairment and dementia predicted nursing home placement [64].

### 4.2 Implications for Practice

To enable the delivery of sustainable equitable social care and targeted prevention strategies, robust evidence is required on the causal relationship of the health and social conditions that drive the need for and use of social care. Regardless of how a country organizes and funds care, all face the challenge of providing fair and equitable social care [65, 66]. With limited public funding, reliance on unpaid informal carers when available will continue to increase [67]. This risks further disadvantaging populations who are already disadvantaged, and those living alone [57].

### 4.3 Implications for Research

Future research to strengthen the evidence of the impact of social conditions on a loss of independence and identify modifiable risk factors for effective intervention, such as prevention and improved disease management, is a long‐standing priority to reduce a loss of independence in populations most at risk [2, 3, 68, 69]. Large follow‐up studies with detailed health data have potential to generate evidence on the impact of social conditions in populations with one or more long‐term condition and the concepts underpinning the causal relationship with the need for care. Although complex, a qualitative evidence synthesis might inform the concepts underpinning need, demand, use and expenditure on care. Heterogeneity in the measures of common social conditions, such deprivation, partly reflected the availability of data but with wider adoption of reporting standards this could be improved [61, 70, 71]. On‐going research on assessing the risk of bias for cross‐sectional studies might address the lack of comparability between measures [27].

## 5 Conclusion

An increasing need for social care is a societal challenge, that has the potential to widen inequalities in the need for and use of social care. Without an improved evidence‐base on the social conditions that drive a loss of independence it will not be possible to accurately identify the populations at most risk of needing care.

## Author Contributions

**Anna Mae Scott:** conceptualization, screening and selecting studies, data extraction, risk of bias assessments, review and editing the manuscript; **Ben Amies‐Cull:** conceptualization, screening and selection of studies, data extraction, review and editing the manuscript; **Emre Oguzman:** data curation (maintaining datasets and code for both the static and online EGM), visualization (creation of the EGM), and review and editing the manuscript; **James Webster:** selection of studies, risk of bias assessment, review and editing the manuscript; **Rhys Llewellyn Thomas:** narrative interpretation of the results, review and editing of the manuscript; **Anne Mason:** funding acquisition, conceptualization, screening and selection of studies, review and editing the manuscript; **Laura Bojke:** funding acquisition, conceptualization, screening and selection of studies, review and editing of the manuscript; **Matthew Quinn:** study screening and selection of studies, review and editing of the manuscript. **Paul Clarkson:** conceptualization, review and editing of the manuscript; **Joe Spearing:** screening and selection of studies, review and editing the manuscript; **Sasha Shepperd:** funding acquisition, conceptualization, screening and selection of studies, data extraction, risk of bias assessment, writing the original and subsequent drafts of the manuscript.

## Conflicts of Interest

The authors declare no conflicts of interest.

## Supporting information


Supporting File 1

Supporting File 2

Supporting File 3

Supporting File 4

Supporting File 5

Supporting File 6

Supporting File 7

## Data Availability

The data that support the findings of this study are available in the supporting material of this article.
