# Supplementary material for: Social and Health Conditions That Drive the Need and Demand for, Use of and Expenditure on Social Care in the UK: A Systematic Review
Source: Cochrane Evid Synth Methods. 2026 Aug 12;4(5):e70093. doi: 10.1002/cesm.70093 (PMC13489229; doi:10.1002/cesm.70093)
Supplement: Supplementary file 1 [file CESM-4-e70093-s001.docx]

# Table S2 – Sources of data

| ***Author*** | ***Data source*** | ***Social condition & measure*** | ***Health condition & measure*** |
| --- | --- | --- | --- |
| **Burton 2018** | Combined the intervention and control groups from the PROSPER randomised trial, recruitment 1997 to 1999, follow-up 2012 to 2015, for a multivariate analysis of social condition; linked Scottish health and social care data: (1) the Prescribing Information System (which indicates if a drug prescription was issued to a patient registered in a long-term care facility); (2) Scottish mortality register (which indicates if a patient attending secondary care was discharged to a long-term care facility); and (3) the care-home census (a national annual census of long-term care residents). [N.B. Also analysed data from the WOSCOPS randomised trial, excluded from our review due to age <65 years] | Age, sex |  |
| **Chukwusa 2022** | ONS death registry data  NHS Digital Adult Social Care Activity and Finance Report for the period of 2018-19 | Sex, marital status, location at time of death (urban/rural), government office region, underlying and contributory cause of death, place of death and IMD score | Diagnosis of diabetes, cardiovascular diseases that included hypertension and stroke, dementia, chronic kidney diseases |
| **Davies 2022** | Newcastle 85+ cohort | Age, sex, education, Index of Multiple Deprivation (IMD) <25th centile 25% (N=213) (income, employment, crime, living environment, education, health and barriers to housing and services, in various proportions, to produce an overall index) | Measured by presence/absence of one of nine chronic diseases: arthritis, diabetes, hypertension, cardiac disease, chronic obstructive pulmonary disease, other respiratory disease, stroke, other cerebrovascular disease and cancer in the past 5 years excluding non-melanoma skin cancer. Multi-dimensional health assessment in the person's residence by a trained research nurse and GP records at baseline, 18, 36, 60 and 120 months  Mobility disability, based on self-reported ability to get around the house, go up and down the stairs/steps and walk at least 400 yards measured by the validated Groningen Activity Restriction Scale; mobility disability present if the response was yes to “I have some difficulty doing this by myself,” or “I can only do this by myself if I use an aid or appliance”) or could not perform them (responding yes to “I am unable to do this by myself, I need someone else’s help. |
| **Dunatchik 2019 & Dunatchik 2016 (IPSO report)** | ELSA wave 1 (2002) and 6 (2012.)  At baseline, in 2002–2003, the sample comprised 11,391 core participants who were recruited from households that had earlier participated in the Health Survey for England (HSE) in 1998, 1999 and 2001 | Age, gender, household composition, spousal loss, housing tenure | Long-term conditions, ADLs, cognitive function, quality of life, poor eye sight, smoking, physical activity |
| **Gouisa 2020** | ELSA 8 waves from 2002/03 to 2016/17 | Age, sex, education, family structure, working status, ethnicity, wealth quintiles, region in England | Diagnosis of cancer vs no cancer diagnosis, a long-term condition (one of arthritis, high blood pressure, asthma, diabetes, osteoporosis, dementia) and pain |
| **Henderson 2019 (baseline IDEAL data)**  **Henderson 2022 (3 waves of IDEAL)**  **Sabatini 2024 (3 waves of IDEAL)** | Improving the Experience of Dementia and Enhancing Active Life (IDEAL) waves 1 to 3 2014 to 2018; participants followed-up annually by face-to-face completion of questionnaires and self-completion for caregivers.  The caregiver questionnaire (lost working time, unpaid care provided by the caregiver and by other relatives/friends at Waves 2 and 3) and from participant questionnaire data (unpaid care received from all relatives/friends at Waves 2 and 3). | Age, sex, living alone, dementia type, family or friend caregiver (ref spouse caregiver), no caregiver | Clinical diagnosis of dementia; dementia type (vascular, frontotemporal, Parkinson’s Disease Dementia, dementia with Lewy bodies, unspecified)  Validated Mini-Mental State Examination (score 15 or higher for inclusion) |
| **Hu 2022** | ELSA waves 6–9 (2012–2018) | Age, sex, marital status, education, ethnicity, occupation, housing problems, geographical region, living with children (identified from the Behavioural Model of Care Utilisation); income, wealth, occupation, housing problems, regions in England aggregated to three regions | n/a |
| **Hu 2024** | ELSA waves 6, 7 and 8; 2012, 2014, 2016 | Calculated a concentration index to measure socio-economic inequality in care utilisation.  Age and sex measured as need factors that determined care utilisation; marital status, living with children in the same household, level of education, equivalised income and total net wealth were non-need factors that determined care utilisation. | Validated measures: Informant Questionnaire on Cognitive Decline in the Elderly (IQCODE), functional ability measured by ELSA, depression using the Centre for Epidemiology Studies Depression Scale, self-reported health on a 5 points Likert scale and illness were measured as need factors that determined care utilisation |
| **Iparraguire 2020** | ELSA wave 2 2004/2005 to wave 6 2012/2013  Council with Adult Social Services  Responsibility (CASSR) data  2015/16 prices | Age, sex, income, non-housing wealth, household size, income deprivation (the proportion of older people who receive low-income benefits by local area. Rural/Urban classification by local authority area. |  |
| **Kasteridis 2015** | Linked health and social care data for the entire South Somerset population for the financial year 2012/2013 | Age, sex, deprivation | Number of chronic conditions  Chronic conditions included diabetes, cardiac disease, chronic obstructive pulmonary disease or occupational pulmonary disease, chronic kidney disease or renal failure, depression or anxiety, dementia, stroke and cancer. |
| **Knapp 2016** | Data extracted from routine care data derived from electronic medical records of the South London and Maudsley National Health Service (NHS) Foundation Trust (SLAM). Data available from 2008, extracted in 2012. | Age, sex, partner (yes/no); ethnicity, living alone (yes/no); living conditions (not a problem; minor problems; significant problems); relationship problems (not a problem; minor problems; significant problems), housing problems/living conditions; relationship problems | ADL (not a problem; minor problems; significant problems); physical illness (not a problem; minor problems; significant problems); agitated (not a problem; minor problems; significant problems); depression (not a problem; minor problems; significant problems); the validated Mini-Mental State Examination for cognition, and Health of the Nation Outcomes Scale for ADLs, physical illness, disability, agitated behaviour, depression |
| **Lenox-Smith 2016** | GERAS a prospective follow-up study, a multi-centre European study including UK, France and Germany, 2010 data. | NR | Dementia, defined by the National Institute of Neurological and Communicative Disorders, and Stroke and Alzheimer’s Disease and Related Disorders Association (NINCDS-ADRDA) criteria [21], the validated Mini-Mental State examination (MMSE) score of ≤26, and presented within the normal course of care (i.e., for the diagnosis, treatment, or follow-up care of AD). |
| **MacLeod 2021** | Cognitive Function and Ageing Study Wales (CFAS Wales), participants selected from primary care lists from 2011 to 2013. | Socio-economic status, occupation,  deprivation | Self-reported health: (excellent, good, fair or poor for someone their age)  Cognitive function (validated measures): Mini Mental State Examination score of <26=cognitive impairment or dementia diagnosed by Geriatric Mental State - Automated Geriatric Examination for Computer-Assisted Taxonomy or a clinical diagnosis by a medically qualified member of the study team  Activities of daily living (no disability, IADL disability only, or ADL and IADL disabilities) |
| **Nakubulwa 2022** | Pseudo-anonymised person-level data from an integrated care dataset (Northwest London Discover Database) in two NW London boroughs; 2012 to 2018 | Sex  Age  Ethnicity  IMD quintiles (IMD 1 reference, IMD 4 to 5 least deprived)  Household structure  Disabilities | Multi-morbidity measured by number and type of long-term conditions [disability, autoimmune, cardiovascular disease, respiratory disease, malignant disease, endocrine diseases, neurological diseases, mental health end of life], emergency admissions, day electives, outpatients, accidence and emergency arrivals by ambulance, X-ray investigations |
| **Nikolova 2022** | Community Ageing Research 75+ (CARE75+) 2018 data; ELSA (wave 6) 2012/13 data; and ONS population estimates 2020 | Age, sex, education, living with partner | Frailty defined in both data sets using the validated frailty index (FI) |
| **Turner 2015** | NHS Health and Social Care Information Centre (HSCIC); 2009/10 and 2010/11 | IMD - income deprivation, living alone/not living alone | Hip or knee replacement |
| **Vlachantoni 2015** | ELSA wave 4 2008 data | Demographic and socio-economic characteristics | Difficulty with ADL, IADL, mobility using validated ELSA measures |
| **Vlachantoni 2019** | ELSA wave 7 2014/15 data | Sex, education, occupation, marital status, housing tenure, | Difficulty with ADLs, IADL or mobility using validated ELSA measures |

**References to validated measured of outcome list**

The Centre for Epidemiology Studies Depression Scale

Seong-Hi Park, Hye Yon Yu. How useful is the Center for Epidemiologic Studies Depression Scale in screening for depression in adults? An updated systematic review and meta-analysis. Psychiatry Research, Volume 302, 2021, 114037, ISSN 0165-1781, https://doi.org/10.1016/j.psychres.2021.114037.

Geriatric Mental State:

Copeland, J. R. M., Dewey, M. E., & Saunders, P. (1991). The epidemiology of dementia: GMS-AGECAT studies of prevalence and incidence, including studies in progress. *European Archives of Psychiatry and Clinical Neuroscience, 240*(4), 212–217. https:// doi.org/10.1007/bf02189529

Groningen Activity Restriction Scale Groningen Activity Restriction Scale

Kempen, G. I. J. M., Miedema, I., Ormel, J., & Molenaar, W. (1996). Groningen Activity Restriction Scale (GARS) . APA PsycTests. [https://doi.org/10.1037/t11080-000](https://psycnet.apa.org/doi/10.1037/t11080-000)

Mini-Mental State Examination

Folstein MF, Folstein SE, McHugh PR. Mini-mental state: a practical method for grading the cognitive state of patients for the clinician. J Psychiatr Res 1975;12:189–98.

Frailty Index:

Mitnitski, A. B., Mogilner, A. J., & Rockwood, K. (2001). Accumulation of deficits as a proxy measure of aging. *The Scientific World Journal*, *1*, 323–336.https://doi.org/10.1100/tsw.2001.58 Mitnitski, A. B., Mogilner, A. J., & Rockwood, K. (2001). Accumulation of deficits as a proxy measure of aging. *The Scientific World Journal*, *1*, 323–336. https://doi.org/10.1100/tsw.2001.58

Health of the Nation Outcome Scales

Pirkis JE, Burgess PM, Kirk PK, et al. A review of the psychometric properties of the Health of the Nation Outcome Scales (HoNOS) family of measures. Health Qual Life Outcomes 2005; 3:76.

IQCODE:

Quinn TJ, Fearon P, Noel-Storr AH, Young C, McShane R and Stott DJ (2014) Informant Questionnaire on Cognitive Decline in the Elderly (IQCODE) for the diagnosis of dementia within community dwelling populations. Cochrane Database of Systematic Reviews 2014, CD010079.
