# Supplementary material for: Social and Health Conditions That Drive the Need and Demand for, Use of and Expenditure on Social Care in the UK: A Systematic Review
Source: Cochrane Evid Synth Methods. 2026 Aug 12;4(5):e70093. doi: 10.1002/cesm.70093 (PMC13489229; doi:10.1002/cesm.70093)
Supplement: Supplementary file 2 [file CESM-4-e70093-s002.docx]

**Search strategy (final version)**

**The search dates were 1 January 2009 to 13 June 2024, and updated on 14 April 2025:**

# PubMed <https://pubmed.ncbi.nlm.nih.gov/>

(("predict*"[Title/Abstract] OR "determinant*"[Title/Abstract] OR "driver*"[Title/Abstract] OR "projecti*"[Title/Abstract] OR "promot*"[Title/Abstract]))

AND

("Health Services Needs and Demand"[MeSH Terms:noexp] OR "need*"[Title/Abstract] OR "demand*"[Title/Abstract] OR "using"[Title/Abstract] OR "utilis*"[Title/Abstract] OR "utiliz*"[Title/Abstract] OR "access*"[Title/Abstract] OR "Health Expenditures"[MeSH Terms:noexp] OR "resourc*"[Title/Abstract])

AND

("long term care"[MeSH Terms:noexp] OR "long term care"[Title/Abstract] OR "Social Work"[MeSH Terms:noexp] OR "social work*"[Title/Abstract] OR "formal care"[Title/Abstract] OR "informal care"[Title/Abstract] OR "Home Care Services"[MeSH Terms:noexp] OR "home care"[Title/Abstract] OR "homecare"[Title/Abstract] OR "communit*"[Title/Abstract] OR "Community Health Services"[MeSH Terms:noexp] OR "social service*"[Title/Abstract] OR "social care"[Title/Abstract])

AND

**(2009/01/01:2024/12/31[dp])**

AND

(("United Kingdom"[MeSH Terms] OR ("national health service*"[Title/Abstract] OR "nhs"[Title/Abstract]) OR ("gb"[Title/Abstract] OR "g b"[Title/Abstract] OR "britain*"[Title/Abstract] OR ("british*"[Title/Abstract] NOT "british columbia"[Title/Abstract]) OR "uk"[Title/Abstract] OR "u k"[Title/Abstract] OR "united kingdom*"[Title/Abstract] OR ("england*"[Title/Abstract] NOT "new england"[Title/Abstract]) OR "northern ireland*"[Title/Abstract] OR "northern irish*"[Title/Abstract] OR "scotland*"[Title/Abstract] OR "scottish*"[Title/Abstract] OR (("wales"[Title/Abstract] OR "south wales"[Title/Abstract]) NOT "new south wales"[Title/Abstract]) OR "welsh*"[Title/Abstract]) OR ("bath"[Title/Abstract] OR "bath's"[Title/Abstract] OR (("birmingham"[Title/Abstract] NOT "alabama*"[Title/Abstract]) OR ("birmingham's"[Title/Abstract] NOT "alabama*"[Title/Abstract]) OR "bradford"[Title/Abstract] OR "bradford's"[Title/Abstract] OR "brighton"[Title/Abstract] OR "brighton's"[Title/Abstract] OR "bristol"[Title/Abstract] OR "bristol's"[Title/Abstract] OR "carlisle*"[Title/Abstract] OR ("cambridge"[Title/Abstract] NOT ("massachusetts*"[Title/Abstract] OR "boston*"[Title/Abstract] OR "harvard*"[Title/Abstract])) OR ("canterbury"[Title/Abstract] NOT "zealand*"[Title/Abstract]) OR "chelmsford"[Title/Abstract] OR "chester"[Title/Abstract] OR "chester's"[Title/Abstract] OR "chichester"[Title/Abstract] OR "coventry"[Title/Abstract] OR "coventry's"[Title/Abstract] OR "derby"[Title/Abstract] OR "derby's"[Title/Abstract] OR ("durham"[Title/Abstract] NOT ("carolina*"[Title/Abstract] OR "nc"[Title/Abstract])) OR ("durham's"[Title/Abstract] NOT ("carolina*"[Title/Abstract] OR "nc"[Title/Abstract])) OR "ely"[Title/Abstract] OR "ely's"[Title/Abstract] OR "exeter"[Title/Abstract] OR "gloucester"[Title/Abstract] OR "hereford"[Title/Abstract] OR "hull"[Title/Abstract] OR "hull's"[Title/Abstract] OR "lancaster"[Title/Abstract] OR "lancaster's"[Title/Abstract] OR "leeds*"[Title/Abstract] OR "leicester"[Title/Abstract] OR ("lincoln"[Title/Abstract] NOT "nebraska*"[Title/Abstract]) OR ("lincoln's"[Title/Abstract] NOT "nebraska*"[Title/Abstract]) OR ("liverpool"[Title/Abstract] NOT ("new south wales*"[Title/Abstract] OR "nsw"[Title/Abstract])) OR ("liverpool's"[Title/Abstract] NOT ("new south wales*"[Title/Abstract] OR "nsw"[Title/Abstract])) OR (("london"[Title/Abstract] NOT ("ontario*"[Title/Abstract] OR "ont"[Title/Abstract] OR "toronto*"[Title/Abstract])) OR ("london's"[Title/Abstract] NOT ("ontario*"[Title/Abstract] OR "ont"[Title/Abstract] OR "toronto*"[Title/Abstract])) OR "manchester"[Title/Abstract] OR "manchester's"[Title/Abstract] OR ("newcastle"[Title/Abstract] NOT ("new south wales*"[Title/Abstract] OR "nsw"[Title/Abstract])) OR ("newcastle's"[Title/Abstract] NOT ("new south wales*"[Title/Abstract] OR "nsw"[Title/Abstract])) OR "norwich"[Title/Abstract] OR "nottingham"[Title/Abstract] OR "nottingham's"[Title/Abstract] OR "oxford"[Title/Abstract] OR "oxford's"[Title/Abstract] OR "peterborough"[Title/Abstract] OR "plymouth"[Title/Abstract] OR "plymouth's"[Title/Abstract] OR "portsmouth"[Title/Abstract] OR "preston"[Title/Abstract] OR "preston's"[Title/Abstract] OR "ripon"[Title/Abstract] OR "salford"[Title/Abstract] OR "salisbury"[Title/Abstract] OR "salisbury's"[Title/Abstract] OR "sheffield"[Title/Abstract] OR "sheffield's"[Title/Abstract] OR "southampton"[Title/Abstract] OR "southampton's"[Title/Abstract] OR "st albans"[Title/Abstract] OR "stoke"[Title/Abstract] OR "stoke's"[Title/Abstract] OR "sunderland"[Title/Abstract] OR "sunderland's"[Title/Abstract] OR "truro"[Title/Abstract] OR "wakefield"[Title/Abstract] OR "wakefield's"[Title/Abstract] OR "wells"[Title/Abstract] OR "westminster"[Title/Abstract] OR "winchester"[Title/Abstract] OR "wolverhampton"[Title/Abstract] OR ("worcester"[Title/Abstract] NOT ("massachusetts*"[Title/Abstract] OR "boston*"[Title/Abstract] OR "harvard*"[Title/Abstract])) OR ("york"[Title/Abstract] NOT ("new york*"[Title/Abstract] OR "ny"[Title/Abstract] OR "ontario*"[Title/Abstract] OR "ont"[Title/Abstract] OR "toronto*"[Title/Abstract])) OR ("york's"[Title/Abstract] NOT ("new york*"[Title/Abstract] OR "ny"[Title/Abstract] OR "ontario*"[Title/Abstract] OR "ont"[Title/Abstract] OR "toronto*"[Title/Abstract]))))) OR ("bangor"[Title/Abstract] OR "cardiff"[Title/Abstract] OR "newport"[Title/Abstract] OR "newport's"[Title/Abstract] OR "st asaph"[Title/Abstract] OR "st davids"[Title/Abstract] OR "swansea"[Title/Abstract]) OR ("aberdeen"[Title/Abstract] OR "dundee"[Title/Abstract] OR "dundee's"[Title/Abstract] OR "edinburgh"[Title/Abstract] OR "edinburgh's"[Title/Abstract] OR "glasgow"[Title/Abstract] OR "glasgow's"[Title/Abstract] OR "inverness"[Title/Abstract] OR ("perth"[Title/Abstract] NOT "australia*"[Title/Abstract]) OR ("perth's"[Title/Abstract] NOT "australia*"[Title/Abstract]) OR "stirling"[Title/Abstract] OR "stirling's"[Title/Abstract]) OR ("armagh"[Title/Abstract] OR "belfast"[Title/Abstract] OR "lisburn"[Title/Abstract] OR "londonderry"[Title/Abstract] OR "derry"[Title/Abstract] OR "derry's"[Title/Abstract] OR "newry"[Title/Abstract])) NOT ("africa"[MeSH Terms] OR "americas"[MeSH Terms] OR "antarctic regions"[MeSH Terms] OR "arctic regions"[MeSH Terms] OR "asia"[MeSH Terms] OR "oceania"[MeSH Terms])) NOT ("United Kingdom"[MeSH Terms] OR "europe"[MeSH Terms:noexp])

## CINAHL (via Ebsco)

NB: adjusted the “community” in PubMed’s version to “community care” in CINAHL

((TI predict* OR AB predict*) OR (TI determinant* OR AB determinant*) OR (TI driver* OR AB driver*) OR (TI projecti* OR AB projecti*) OR (TI promot* OR AB promot*))

AND

((MH "Health Services Needs and Demand") OR (TI need* OR AB need*) OR (TI demand* OR AB demand*) OR (TI using OR AB using) OR (TI utilis* OR AB utilis*) OR (TI utiliz* OR AB utiliz*) OR (TI access* OR AB access*) OR (MH "Health Expenditures") OR (TI resourc* OR AB resourc*))

AND

((MH "long term care") OR (TI "long term care" OR AB "long term care") OR (MH "Social Work") OR (TI "social work*" OR AB "social work*") OR (TI "formal care" OR AB "formal care") OR (TI "community care" OR AB "community care") OR (TI "informal care" OR AB "informal care") OR (MH "Home Care Services") OR (TI "home care" OR AB "home care") OR (TI homecare OR AB homecare) OR (MH "Community Health Services") OR (TI "social service*" OR AB "social service*") OR (TI "social care" OR AB "social care"))

AND

(((MH "United Kingdom+") OR ((TI "national health service*" OR AB "national health service*") OR (TI nhs OR AB nhs)) OR ((TI gb OR AB gb) OR (TI "g b" OR AB "g b") OR (TI britain* OR AB britain*) OR ((TI british* OR AB british*) NOT (TI "british columbia" OR AB "british columbia")) OR (TI uk OR AB uk) OR (TI "u k" OR AB "u k") OR (TI "united kingdom*" OR AB "united kingdom*") OR ((TI england* OR AB england*) NOT (TI "new england" OR AB "new england")) OR (TI "northern ireland*" OR AB "northern ireland*") OR (TI "northern irish*" OR AB "northern irish*") OR (TI scotland* OR AB scotland*) OR (TI scottish* OR AB scottish*) OR (((TI wales OR AB wales) OR (TI "south wales" OR AB "south wales")) NOT (TI "new south wales" OR AB "new south wales")) OR (TI welsh* OR AB welsh*)) OR ((TI bath OR AB bath) OR (TI bath's OR AB bath's) OR (((TI birmingham OR AB birmingham) NOT (TI alabama* OR AB alabama*)) OR ((TI birmingham's OR AB birmingham's) NOT (TI alabama* OR AB alabama*)) OR (TI bradford OR AB bradford) OR (TI bradford's OR AB bradford's) OR (TI brighton OR AB brighton) OR (TI brighton's OR AB brighton's) OR (TI bristol OR AB bristol) OR (TI bristol's OR AB bristol's) OR (TI carlisle* OR AB carlisle*) OR ((TI cambridge OR AB cambridge) NOT ((TI massachusetts* OR AB massachusetts*) OR (TI boston* OR AB boston*) OR (TI harvard* OR AB harvard*))) OR ((TI canterbury OR AB canterbury) NOT (TI zealand* OR AB zealand*)) OR (TI chelmsford OR AB chelmsford) OR (TI chester OR AB chester) OR (TI chester's OR AB chester's) OR (TI chichester OR AB chichester) OR (TI coventry OR AB coventry) OR (TI coventry's OR AB coventry's) OR (TI derby OR AB derby) OR (TI derby's OR AB derby's) OR ((TI durham OR AB durham) NOT ((TI carolina* OR AB carolina*) OR (TI nc OR AB nc))) OR ((TI durham's OR AB durham's) NOT ((TI carolina* OR AB carolina*) OR (TI nc OR AB nc))) OR (TI ely OR AB ely) OR (TI ely's OR AB ely's) OR (TI exeter OR AB exeter) OR (TI gloucester OR AB gloucester) OR (TI hereford OR AB hereford) OR (TI hull OR AB hull) OR (TI hull's OR AB hull's) OR (TI lancaster OR AB lancaster) OR (TI lancaster's OR AB lancaster's) OR (TI leeds* OR AB leeds*) OR (TI leicester OR AB leicester) OR ((TI lincoln OR AB lincoln) NOT (TI nebraska* OR AB nebraska*)) OR ((TI lincoln's OR AB lincoln's) NOT (TI nebraska* OR AB nebraska*)) OR ((TI liverpool OR AB liverpool) NOT ((TI "new south wales*" OR AB "new south wales*") OR (TI nsw OR AB nsw))) OR ((TI liverpool's OR AB liverpool's) NOT ((TI "new south wales*" OR AB "new south wales*") OR (TI nsw OR AB nsw))) OR (((TI london OR AB london) NOT ((TI ontario* OR AB ontario*) OR (TI ont OR AB ont) OR (TI toronto* OR AB toronto*))) OR ((TI london's OR AB london's) NOT ((TI ontario* OR AB ontario*) OR (TI ont OR AB ont) OR (TI toronto* OR AB toronto*))) OR (TI manchester OR AB manchester) OR (TI manchester's OR AB manchester's) OR ((TI newcastle OR AB newcastle) NOT ((TI "new south wales*" OR AB "new south wales*") OR (TI nsw OR AB nsw))) OR ((TI newcastle's OR AB newcastle's) NOT ((TI "new south wales*" OR AB "new south wales*") OR (TI nsw OR AB nsw))) OR (TI norwich OR AB norwich) OR (TI nottingham OR AB nottingham) OR (TI nottingham's OR AB nottingham's) OR (TI oxford OR AB oxford) OR (TI oxford's OR AB oxford's) OR (TI peterborough OR AB peterborough) OR (TI plymouth OR AB plymouth) OR (TI plymouth's OR AB plymouth's) OR (TI portsmouth OR AB portsmouth) OR (TI preston OR AB preston) OR (TI preston's OR AB preston's) OR (TI ripon OR AB ripon) OR (TI salford OR AB salford) OR (TI salisbury OR AB salisbury) OR (TI salisbury's OR AB salisbury's) OR (TI sheffield OR AB sheffield) OR (TI sheffield's OR AB sheffield's) OR (TI southampton OR AB southampton) OR (TI southampton's OR AB southampton's) OR (TI "st albans" OR AB "st albans") OR (TI stoke OR AB stoke) OR (TI stoke's OR AB stoke's) OR (TI sunderland OR AB sunderland) OR (TI sunderland's OR AB sunderland's) OR (TI truro OR AB truro) OR (TI wakefield OR AB wakefield) OR (TI wakefield's OR AB wakefield's) OR (TI wells OR AB wells) OR (TI westminster OR AB westminster) OR (TI winchester OR AB winchester) OR (TI wolverhampton OR AB wolverhampton) OR ((TI worcester OR AB worcester) NOT ((TI massachusetts* OR AB massachusetts*) OR (TI boston* OR AB boston*) OR (TI harvard* OR AB harvard*))) OR ((TI york OR AB york) NOT ((TI "new york*" OR AB "new york*") OR (TI ny OR AB ny) OR (TI ontario* OR AB ontario*) OR (TI ont OR AB ont) OR (TI toronto* OR AB toronto*))) OR ((TI york's OR AB york's) NOT ((TI "new york*" OR AB "new york*") OR (TI ny OR AB ny) OR (TI ontario* OR AB ontario*) OR (TI ont OR AB ont) OR (TI toronto* OR AB toronto*)))))) OR ((TI bangor OR AB bangor) OR (TI cardiff OR AB cardiff) OR (TI newport OR AB newport) OR (TI newport's OR AB newport's) OR (TI "st asaph" OR AB "st asaph") OR (TI "st davids" OR AB "st davids") OR (TI swansea OR AB swansea)) OR ((TI aberdeen OR AB aberdeen) OR (TI dundee OR AB dundee) OR (TI dundee's OR AB dundee's) OR (TI edinburgh OR AB edinburgh) OR (TI edinburgh's OR AB edinburgh's) OR (TI glasgow OR AB glasgow) OR (TI glasgow's OR AB glasgow's) OR (TI inverness OR AB inverness) OR ((TI perth OR AB perth) NOT (TI australia* OR AB australia*)) OR ((TI perth's OR AB perth's) NOT (TI australia* OR AB australia*)) OR (TI stirling OR AB stirling) OR (TI stirling's OR AB stirling's)) OR ((TI armagh OR AB armagh) OR (TI belfast OR AB belfast) OR (TI lisburn OR AB lisburn) OR (TI londonderry OR AB londonderry) OR (TI derry OR AB derry) OR (TI derry's OR AB derry's) OR (TI newry OR AB newry))) NOT ((MH africa+) OR (MH americas+) OR (MH "antarctic regions+") OR (MH "arctic regions+") OR (MH asia+) OR (MH oceania+))) NOT ((MH "United Kingdom+") OR (MH europe))

AND

Limiters - Publication Date: 20090101-20241231

## EconLit (via Proquest)

((TI,AB(predict*) OR TI,AB(determinant*) OR TI,AB(driver*) OR TI,AB(projecti*) OR TI,AB(promot*)) AND (TI,AB("health services need") OR TI,AB("health services demand") OR TI,AB(need*) OR TI,AB(demand*) OR TI,AB(using) OR TI,AB(utilis*) OR TI,AB(utiliz*) OR TI,AB(access*) OR TI,AB("health expenditure") OR TI,AB(resourc*)) AND (TI,AB("long term care") OR TI,AB(("social work" OR "social worker" OR "social workers" OR "social works")) OR TI,AB("formal care") OR TI,AB("informal care") OR TI,AB("Home Care Services") OR TI,AB("home care") OR TI,AB(homecare) OR TI,AB(communit*) OR TI,AB("Community Health Services") OR TI,AB(("social service" OR "social services")) OR TI,AB("social care")) AND (((TI,AB("United Kingdom") OR (TI,AB("national health service*") OR TI,AB(nhs)) OR (TI,AB(gb) OR TI,AB("g b") OR TI,AB(britain*) OR (TI,AB(british*) NOT TI,AB("british columbia")) OR TI,AB(uk) OR TI,AB("u k") OR TI,AB(("united kingdom" OR "united kingdoma" OR "united kingdomin" OR "united kingdoms" OR "united kingdomthe")) OR (TI,AB(england*) NOT TI,AB("new england")) OR TI,AB(("northern ireland" OR "northern irelandin" OR "northern irelands" OR "northern irelandthe")) OR TI,AB(("northern irish" OR "northern irishman")) OR TI,AB(scotland*) OR TI,AB(scottish*) OR ((TI,AB(wales) OR TI,AB("south wales")) NOT TI,AB("new south wales")) OR TI,AB(welsh*)) OR (TI,AB(bath) OR TI,AB(bath's) OR ((TI,AB(birmingham) NOT TI,AB(alabama*)) OR (TI,AB(birmingham's) NOT TI,AB(alabama*)) OR TI,AB(bradford) OR TI,AB(bradford's) OR TI,AB(brighton) OR TI,AB(brighton's) OR TI,AB(bristol) OR TI,AB(bristol's) OR TI,AB(carlisle*) OR (TI,AB(cambridge) NOT (TI,AB(massachusetts*) OR TI,AB(boston*) OR TI,AB(harvard*))) OR (TI,AB(canterbury) NOT TI,AB(zealand*)) OR TI,AB(chelmsford) OR TI,AB(chester) OR TI,AB(chester's) OR TI,AB(chichester) OR TI,AB(coventry) OR TI,AB(coventry's) OR TI,AB(derby) OR TI,AB(derby's) OR (TI,AB(durham) NOT (TI,AB(carolina*) OR TI,AB(nc))) OR (TI,AB(durham's) NOT (TI,AB(carolina*) OR TI,AB(nc))) OR TI,AB(ely) OR TI,AB(ely's) OR TI,AB(exeter) OR TI,AB(gloucester) OR TI,AB(hereford) OR TI,AB(hull) OR TI,AB(hull's) OR TI,AB(lancaster) OR TI,AB(lancaster's) OR TI,AB(leeds*) OR TI,AB(leicester) OR (TI,AB(lincoln) NOT TI,AB(nebraska*)) OR (TI,AB(lincoln's) NOT TI,AB(nebraska*)) OR (TI,AB(liverpool) NOT (TI,AB("new south wales*") OR TI,AB(nsw))) OR (TI,AB(liverpool's) NOT (TI,AB("new south wales*") OR TI,AB(nsw))) OR ((TI,AB(london) NOT (TI,AB(ontario*) OR TI,AB(ont) OR TI,AB(toronto*))) OR (TI,AB(london's) NOT (TI,AB(ontario*) OR TI,AB(ont) OR TI,AB(toronto*))) OR TI,AB(manchester) OR TI,AB(manchester's) OR (TI,AB(newcastle) NOT (TI,AB("new south wales*") OR TI,AB(nsw))) OR (TI,AB(newcastle's) NOT (TI,AB("new south wales*") OR TI,AB(nsw))) OR TI,AB(norwich) OR TI,AB(nottingham) OR TI,AB(nottingham's) OR TI,AB(oxford) OR TI,AB(oxford's) OR TI,AB(peterborough) OR TI,AB(plymouth) OR TI,AB(plymouth's) OR TI,AB(portsmouth) OR TI,AB(preston) OR TI,AB(preston's) OR TI,AB(ripon) OR TI,AB(salford) OR TI,AB(salisbury) OR TI,AB(salisbury's) OR TI,AB(sheffield) OR TI,AB(sheffield's) OR TI,AB(southampton) OR TI,AB(southampton's) OR TI,AB("st albans") OR TI,AB(stoke) OR TI,AB(stoke's) OR TI,AB(sunderland) OR TI,AB(sunderland's) OR TI,AB(truro) OR TI,AB(wakefield) OR TI,AB(wakefield's) OR TI,AB(wells) OR TI,AB(westminster) OR TI,AB(winchester) OR TI,AB(wolverhampton) OR (TI,AB(worcester) NOT (TI,AB(massachusetts*) OR TI,AB(boston*) OR TI,AB(harvard*))) OR (TI,AB(york) NOT (TI,AB(("new york" OR "new yorker" OR "new yorkers" OR "new yorks" OR "new yorkshire" OR "new yorkthe" OR "new yorkthis")) OR TI,AB(ny) OR TI,AB(ontario*) OR TI,AB(ont) OR TI,AB(toronto*))) OR (TI,AB(york's) NOT (TI,AB(("new york" OR "new yorker" OR "new yorkers" OR "new yorks" OR "new yorkshire" OR "new yorkthe" OR "new yorkthis")) OR TI,AB(ny) OR TI,AB(ontario*) OR TI,AB(ont) OR TI,AB(toronto*)))))) OR (TI,AB(bangor) OR TI,AB(cardiff) OR TI,AB(newport) OR TI,AB(newport's) OR TI,AB("st asaph") OR TI,AB("st davids") OR TI,AB(swansea)) OR (TI,AB(aberdeen) OR TI,AB(dundee) OR TI,AB(dundee's) OR TI,AB(edinburgh) OR TI,AB(edinburgh's) OR TI,AB(glasgow) OR TI,AB(glasgow's) OR TI,AB(inverness) OR (TI,AB(perth) NOT TI,AB(australia*)) OR (TI,AB(perth's) NOT TI,AB(australia*)) OR TI,AB(stirling) OR TI,AB(stirling's)) OR (TI,AB(armagh) OR TI,AB(belfast) OR TI,AB(lisburn) OR TI,AB(londonderry) OR TI,AB(derry) OR TI,AB(derry's) OR TI,AB(newry))) NOT (TI,AB (africa) OR TI,AB(americas) OR TI,AB("antarctic regions") OR TI,AB("arctic regions") OR TI,AB(asia) OR TI,AB(oceania))) NOT (TI,AB("United Kingdom") OR TI,AB(europe)))) AND pd(20090101-20241231)

## Applied Social Science Index and Abstracts, ASSIA (via ProQuest)

**NB: used local geographic filter (results for UK, Scotland, England, N Ireland, Wales) rather than NICE’s**

((TI,AB(predict*) OR TI,AB(driver*) OR TI,AB(determinant*) OR TI,AB(projecti*) OR TI,AB(promot*)) AND (MESH.EXACT("Health Services Needs and Demand") OR TI,AB(need*) OR TI,AB(demand*) OR TI,AB(using) OR TI,AB(utilis*) OR TI,AB(utiliz*) OR TI,AB(access*) OR MESH.EXACT("Health Expenditures") OR TI,AB(resourc*)) AND (MESH.EXACT("long term care") OR TI,AB("long term care") OR MESH.EXACT("Social Work") OR TI,AB(("social work" OR "social worker" OR "social workers" OR "social works")) OR TI,AB("formal care") OR TI,AB("informal care") OR MESH.EXACT("Home Care Services") OR TI,AB("home care") OR TI,AB(homecare) OR TI,AB(communit*) OR MESH.EXACT("Community Health Services") OR TI,AB(("social service" OR "social services")) OR TI,AB("social care"))) AND (location.exact("United Kingdom--UK" OR "England" OR "Wales" OR "Scotland" OR "Northern Ireland") AND pd(20090101-20241231))

## Campbell Collaboration search

1. Campbell Subject Category: Ageing AND Systematic reviews AND publication date: 2009 – 2024 [https://onlinelibrary.wiley.com/index/18911803?ConceptID=351939&startPage=&ConceptID=253710]
2. Campbell Subject Category: Social welfare AND Updated systematic reviews AND publication date: 2009 – 2024 [https://onlinelibrary.wiley.com/index/18911803?AfterYear=2009&BeforeYear=2024&ConceptID=229188&content=articlesChapters&journalCode=18911803&target=series-index&startPage=&ConceptID=253708]
3. Campbell Subject Category: Social welfare AND Systematic reviews AND publication date: 2009 – 2024 [https://onlinelibrary.wiley.com/index/18911803?AfterYear=2009&BeforeYear=2024&ConceptID=229188&content=articlesChapters&journalCode=18911803&target=series-index&startPage=&ConceptID=253710 ]
4. 1 and 2 and 3

**(NB: excluded protocols and Evidence Gap Maps)**

## **Gray literature search searched 1^st^ January 2009 to 21^st^ August 2024**

| **Source** | **Website searched** | **Search terms** | **URL for the search results** |
| --- | --- | --- | --- |
| Dept of Health – UK | https://www.gov.uk/government/organisations/department-of-health-and-social-care | Searched: “social care”  <https://www.gov.uk/search/all?keywords=%22social+care%22&order=relevance>  Restrictions on the results:  Topics: Health and social care  Sub-topic: Social care  Content type: restricted to “Research & Statistics” | <https://www.gov.uk/search/all?keywords=%22social+care%22&level_one_taxon=8124ead8-8ebc-4faf-88ad-dd5cbcc92ba8&level_two_taxon=7d67047c-bf22-4c34-b566-b46d6973f961&content_purpose_supergroup%5B%5D=research_and_statistics&public_timestamp%5Bfrom%5D=01/01/2009&order=relevance> |
| *Dept of Health – England* | *NHS England*  *https://www.england.nhs.uk/* | *Searched: “social care:*  *Restrictions on the results:*  *Filter by content type: Publications* | [*https://www.england.nhs.uk/?s=%22social+care%22*](https://www.england.nhs.uk/?s=%22social+care%22)  *[NB: does not provide a new URL with search results after restrictions; have to reenter]* |
| Dept of Health – Scotland | NHS Scotland  <https://www.nss.nhs.scot/> | Searched: “social care”  No search date restriction possible | <https://www.nss.nhs.scot/search?q=%22social+care%22> |
| *Dept of Health – Wales* | *NHS Wales*  [*https://www.nhs.wales/*](https://www.nhs.wales/) | *Searched “social care”*  *No options to restrict results from 1^st^ January 2009* | [*https://www.nhs.wales/search-results/?searchFilter=&Keywords=%22social+care%22&strippedKeywords=%22social+care%22&display=search&newSearch=true&noCache=1&csrf_token=E844372C76E3ABC60C268D466ED6B27B&csrf_token_expires=240729180441306*](https://www.nhs.wales/search-results/?searchFilter=&Keywords=%22social+care%22&strippedKeywords=%22social+care%22&display=search&newSearch=true&noCache=1&csrf_token=E844372C76E3ABC60C268D466ED6B27B&csrf_token_expires=240729180441306) |
| *Dept of Health – N. Ireland* | *Health and Social Care*  *https://online.hscni.net/* | *Searched “social care”*  *No options to restrict results from 1^st^ January 2009* | [*https://online.hscni.net/?s=%22social+care%22*](https://online.hscni.net/?s=%22social+care%22) |
| *Social Care Institute of Excellence* | [*https://www.scie.org.uk/*](https://www.scie.org.uk/) | *https://www.google.com/search?as_q=&as_epq=social+care&as_oq=&as_eq=&as_nlo=&as_nhi=&lr=&cr=&as_qdr=all&as_sitesearch=www.scie.org.uk&as_occt=title&as_filetype=&tbs=*  *googlesites search restricted to 2009 onwards with “social care” in the title* | [*https://www.google.com/search?as_q=&as_epq=social+care&as_oq=&as_eq=&as_nlo=&as_nhi=&lr=&cr=&as_qdr=all&as_sitesearch=www.scie.org.uk&as_occt=title&as_filetype=&tbs*](https://www.google.com/search?as_q=&as_epq=social+care&as_oq=&as_eq=&as_nlo=&as_nhi=&lr=&cr=&as_qdr=all&as_sitesearch=www.scie.org.uk&as_occt=title&as_filetype=&tbs)*=* |
| National Institute for Health and Care Excellence | https://www.nice.org.uk/ | Searched: “social care”  Restrictions:  Guidance programme: “social care guidelines”  *No options to restrict results from 1^st^ January 2009* | <https://www.nice.org.uk/search?q=%22social+care%22&ngt=Social+care+guidelines> |
|  | https://www.journalslibrary.nihr.ac.uk/advancedsearch/ | Project/publication title field: “social care”  Report published after field: January 2009 | [Public Health Research \| NIHR Journals Library](https://www.journalslibrary.nihr.ac.uk/phr)  [Health and Social Care Delivery Research \| NIHR Journals Library](https://www.journalslibrary.nihr.ac.uk/hsdr)  [Health Technology Assessment \| NIHR Journals Library](https://www.journalslibrary.nihr.ac.uk/hta)  *[NB: do not provide a new URL with search results after restrictions]* |
| Nuffield Trust, | https://www.nuffieldtrust.org.uk/ | Search: “social care”  <https://www.nuffieldtrust.org.uk/search?keywords=%22social+care%22>  Filter results:  Content type: research  Years: 2009 onwards | <https://www.nuffieldtrust.org.uk/search?keywords=%22social+care%22&type%5Bresearch%5D=research&tag=&year%5B2024%5D=2024&year%5B2023%5D=2023&year%5B2022%5D=2022&year%5B2021%5D=2021&year%5B2020%5D=2020&year%5B2019%5D=2019&year%5B2018%5D=2018&year%5B2017%5D=2017&year%5B2016%5D=2016&year%5B2015%5D=2015&year%5B2014%5D=2014&year%5B2013%5D=2013&year%5B2012%5D=2012&year%5B2011%5D=2011&year%5B2010%5D=2010&year%5B2009%5D=2009&author>= |
| King’s Fund, | <https://www.kingsfund.org.uk/> | Search: “social care”  <https://www.kingsfund.org.uk/search?term=%22social+care%22>  Filter results  Content type: Data and charts, evidence and consultations, Project, Report  (NB: each time, re-enter “social care” in the search bar, then apply the filters, otherwise the search doesn’t work) | <https://www.kingsfund.org.uk/search?term=%22social+care%22&category=data-and-charts&category=evidence-and-consultations&category=project&category=report> |
| The Health Foundation | <https://www.health.org.uk/> | Search by topic: “social care”  <https://www.health.org.uk/search/topic/164?textsearch> | Restricted by: “data analysis”:  <https://www.health.org.uk/search/long_form_sub_type/491/topic/164?textsearch=&sort_bef_combine=created_DESC> |
| Overton Knowledge Database | Access via SOLO | Published after: Jan 1^st^ 2009  Source: UK or England or N Ireland or Wales or Scotland  Document type: publication  AND…..  Title: social care AND need  Structured as: Title:social care need  Title: social care AND demand  Structured as: Title:social care demand  Title: social care AND use  Structured as: Title:social care use  Title: social care AND utilisation [tested also the spelling with Z]  Structured as: Title:social care utilisation  Title: social care AND use  Structured as: Title:social care use | Social care need  <https://app.overton.io/documents.php?overton_policy_document_series=Publication&source_country=_%3Adwi&published_after=2009-01-01&sort=relevance&query=title%3Asocial+care+need>  Social care demand  <https://app.overton.io/documents.php?overton_policy_document_series=Publication&source_country=_%3Adwi&published_after=2009-01-01&sort=relevance&query=title%3Asocial+care+demand>  Social care use  <https://app.overton.io/documents.php?overton_policy_document_series=Publication&source_country=_%3Adwi&published_after=2009-01-01&sort=relevance&query=title%3Asocial+care+use>  Social care utilisation [no results for spelling with Z, either)  <https://app.overton.io/documents.php?overton_policy_document_series=Publication&source_country=_%3Adwi&published_after=2009-01-01&sort=relevance&query=title%3Asocial+care+utilisation>  Social care expenditure  <https://app.overton.io/documents.php?overton_policy_document_series=Publication&source_country=_%3Adwi&published_after=2009-01-01&sort=relevance&query=title%3Asocial+care+expenditure> |
