# Supplementary material for: Social and Health Conditions That Drive the Need and Demand for, Use of and Expenditure on Social Care in the UK: A Systematic Review
Source: Cochrane Evid Synth Methods. 2026 Aug 12;4(5):e70093. doi: 10.1002/cesm.70093 (PMC13489229; doi:10.1002/cesm.70093)
Supplement: Supplementary file 3 [file CESM-4-e70093-s003.docx]

**Appendix 1**

Changes from the protocol

1. We planned to involve members of the Oxford Population Health’s Public Advisory Group (PAG) at all stages of the review process, but due to practical issues that related to funding their involvement was limited to prioritising the review question.
2. We had pre-specified independent double extraction for 15% of the studies, with the remainder single-extracted with an accuracy check. Due to the complex study designs and style of reporting, one author out of AMS and SS extracted data for all studies, with data extracted repeatedly cross checked for all included studies against each study publication. Authors for two studies (Quinn T, for Burton 2022; and Gouisa A 2021) were contacted with questions about the presentation of results.
3. We had prespecified the use of ROBINS-I tool for non-randomised trials but elected to use ROBINS-E as it more closely matched the key elements relevant to assessing bias in the included studies.{Higgins, 2024 #126}
