# Supplementary material for: Social and Health Conditions That Drive the Need and Demand for, Use of and Expenditure on Social Care in the UK: A Systematic Review
Source: Cochrane Evid Synth Methods. 2026 Aug 12;4(5):e70093. doi: 10.1002/cesm.70093 (PMC13489229; doi:10.1002/cesm.70093)
Supplement: Supplementary file 4 [file CESM-4-e70093-s004.docx]

Table S3– Table of results

| Author  Study Design  Follow-up  N= | Need | Demand | Use | Expenditure |
| --- | --- | --- | --- | --- |
| Burton 2018  Combined randomised groups from the PROSPER trial to analyse as a non-randomised follow-up study  3 years (2012-15)  Scottish participants only due to outcome ascertainment for Scotland: n=2033 |  |  | Reduced incident long-term care home admission with male sex (HR 0.72, 95% CI 0.53–0.99), results for females not reported; age (hazard ratio [HR] 1.06 per year, 95% CI 1.03–1.09)  Adjusted for age, sex, LTCs, total cholesterol, smoking, hypertension |  |
| Chukwusa 2022  Cross-sectional population-based area level analysis  n/a  N=409,551  (148/152 local authorities) |  |  |  | Adjusted for age, sex, deprivation and region (local authority income per capita and region)  A percentage point increase in the prevalence of multiple long-term health conditions across regions in England is associated with £8.13 per capita LA social care expenditure p<0.005; did not measure the frequency of care.  Area level analysis based on 148 local authorities  Adjusted for age, sex, deprivation, local authority that included income per capita and region |
| Davies 2022  Prospective cohort study  10 years follow-up (2006-16)  N=845 | Incident mobility disability (defined as difficulty with one or more ADLs/IADLs) adjusted for count of long-term conditions, age, sex, BMI and years in education (Table 2, model 4)  Age: HR 1.02 (0.96-1.09), p=0.55  Female: HR 1.64 (1.25-2.14), p< 0.001  Education (years): HR 0.97 (0.82-1.14)  With each additional long-term condition there was an increased risk of mobility disability (loss of independence): HR 1.16 (1.07-1.25), p< 0.001  BMI 25 to 29.99: HR 1.51 (1.14-2.02), p< 0.05  BMI >=30: HR 1.47 (0.86-2.52), p=0.16  Adjusted for age, sex, deprivation, LTCs, BMI |  |  |  |
|  | Prevalence of unmet need among those with qualifying social care needs (difficulty with one ADL, two IADLs or mobility difficulties; ELSA wave 6: N=918 (58%); and met need N=620 (42%))  Predictors of unmet social need:  Age: (49-54=ref)  Age 60-64: OR 0.70 (95% CI: 0.35-1.41)  Age 65-69: OR 0.70 (95% CI: 0.36 to 1.36)  Age 70-74: OR 0.35 (95% CI 0.19-0.66)  Age 75+: OR 0.24 (95% CI: 0.12-0.47)  Gender: Male (female=ref): OR 1.35 (95% CI: 0.93-1.95)  Living alone (living with others=ref)  OR 1.74 (95% CI 1.08-2.8)  Long-standing illness: (none=ref)  OR 0.56 (95% CI 0.39-0.81)  ADL needs in wave 6 as a predictor of unmet need (none=ref)  OR 14.38 (95% CI 7.48-27.67)  Widowed between waves 1 and 6 (no=ref):  OR 1.71 (95% CI 1.00-2.93) (N.B. not reported by gender)  Education (no education=ref):  Medium education: OR 1.27 (95% CI: 0.84-1.91)  High education: OR 1.05 (95% CI: 0.57-1.93)  NB: The most common difficulties include climbing several flights of stairs, climbing one flight of stairs, walking 100 yards and doing work around the house or garden.  Adjusted for age, sex, household, deprivation, LTCs, smoking status, physical activity, cognitive function, ADLs |  |  |  |
| Dunatchik 2019  Dunatchik 2016  English Longitudinal Survey of Ageing (ELSA)  10 years (waves 1 and 6, 2002-12)  N=1,082 individuals (2,164 observations) | Prevalence of unmet need among those with qualifying social care needs (difficulty with one ADL, two IADLs or mobility difficulties; ELSA wave 6: N=918 (58%); and met need N=620 (42%)  Predictors of unmet social need:  Age: (49-54=ref)  Age 60-64: OR 0.70 (95% CI: 0.35-1.41)  Age 65-69: OR 0.70 (95% CI: 0.36 to 1.36)  Age 70-74: OR 0.35 (95% CI 0.19-0.66)  Age 75+: OR 0.24 (95% CI: 0.12-0.47)  Gender: Male (female=ref): OR 1.35 (95% CI: 0.93-1.95)  Living alone (living with others=ref)  OR 1.74 (95% CI 1.08-2.8)  Long-standing illness: (none=ref)  OR 0.56 (95% CI 0.39-0.81)  ADL needs in wave 6 as a predictor of unmet need (none=ref)  OR 14.38 (95% CI 7.48-27.67)  Widowed between waves 1 and 6 (no=ref):  OR 1.71 (95% CI 1.00-2.93) (N.B. not reported by gender)  Education (no education=ref):  Medium education: OR 1.27 (95% CI: 0.84-1.91)  High education: OR 1.05 (95% CI: 0.57-1.93)  NB: The most common difficulties include climbing several flights of stairs, climbing one flight of stairs, walking 100 yards and doing work around the house or garden.  Adjusted for age, sex, household, deprivation, LTCs, smoking status, physical activity, cognitive function, ADLs |  |  |  |
| Gousia 2020  Cross-sectional  Follow-up: n/a  N=81,338 (ELSA data pooled for all waves) and N=4,767  people living with and beyond cancer (LWBC) | Prevalence of unmet social care needs  Full sample: 0.7 (SD 0.25); 95% CI: 6.1 to 6.5) N=81,338  People LWBC: 0.09 (SD 0.29; 95% CI: 8.3 to 10) N=4,767  Model 1 living with or beyond cancer N=4,767  Odds of having unmet needs:  OR 1.44 (95% CI: 1.20–1.72)  Male OR 1.52 (95% CI 1.32-1.74)  Age OR 1.01 (95% CI 1.00-1.02)  Education (ref no O levels) O-level or equivalent OR 0.72 (95% CI 0.61-0.84)  Education Higher than A-level OR 0.49 (95% CI 0.41-0.58)  Ethnicity/non-white OR 1.4 (95% CI 1.00-1.95)  Arthritis: OR 4.93 (95% CI 4.32-5.61)  High blood pressure OR 1.27 (95% CI 1.14-1.42)  Asthma OR 1.84 (95% CI 1.55-2.18)  Diabetes OR 1.59 (95% CI 1.35-1.88)  Osteoporosis OR 2.18 (95% CI 1.82-2.63)  Dementia OR 2.84 (95% CI 1.93-4.17)  Wealth quintiles (ref=1^st^)  2^nd^: OR 0.65 (95% CI 0.57-0.74)  3^rd^: OR 0.47 (95% CI 0.41-0.55)  4^th^: OR 0.35 (95% CI 0.29-0.41)  5^th^: OR 0.31 (95% CI 0.26-0.38)  Region in England (Ref: South West)  London OR 1.32 (95% CI 1.17–2.01)  East Midlands OR 1.54 (95% CI 1.18–2.02)  West Midlands OR 1.22 (95% CI 0.93–1.60)  North East OR 1.07 (95% CI 0.78–1.46)  North West OR 1.35 (95% CI 1.05–1.75)  Yorkshire and the Humber OR 1.14 (95% CI 0.87–1.51)  South East OR 0.86 (95% CI 0.66–1.11)  East of England OR 0.86 (95% CI 0.66–1.13)  Model 2, full sample N= 81,338  Odds of having unmet needs  OR 1.36 (95% CI: 1.14–1.64)  Male OR 1.64 (95% CI 1.44-1.87)  Age OR 1.01 (95% CI 1.00-1.02)  Education O-level or equivalent OR 0.77 (95% CI 0.66-0.91)  Education Higher than A-level OR 0.58 (95% CI 0.49-0.69)  Ethnicity/non-white OR 1.15 (95% CI 0.83-1.59)  Arthritis OR 2.69 (95% CI 2.37-3.07)  High blood pressure OR 1.20 (95% CI 1.07-1.34)  Asthma OR 1.61 (95% CI 1.36-1.89)  Diabetes OR 1.52 (95% CI 1.29-1.80)  Osteoporosis OR 1.73 (95% CI 1.45-2.08)  Dementia OR 2.79 (95% CI 1.59-4.54)  Pain  Mild often pain OR 2.45 (95% CI 2.06-2.91)  Moderate often pain OR 6.29 (95% CI 5.47-7.24)  Severe often pain OR 18.58 (95% CI 15.87-21.76)  Wealth quintiles (ref=1^st^)  2^nd^: OR 0.74 (95% CI 0.65-0.85)  3^rd^: OR 0.57 (95% CI 0.49-0.66)  4^th^: OR 0.43 (95% CI 0.37-0.51)  5^th^: OR 0.41 (05% CI 0.34-0.50)  Region in England (Ref: South West)  London OR 1.20 (95% CI 0.91 –1.57)  East Midlands OR 1.32 (95% CI 1.02–1.71)  West Midlands OR 1.08 (95% CI 0.83–1.40)  North East 1.01 (95% CI 0.75–1.35)  North West 1.09 (0.86–1.41)  Yorkshire and the Humber 0.97 (95% CI 0.74–1.27)  South East 0.80 (95% CI 0.63–1.04)  East of England 0.82 (95% CI 0.64–1.06)  Adjusted for age, sex, ethnicity, household characteristics, years of education, region, LTS, working status, pain |  |  |  |
| Henderson 2019  Prospective cohort  4 years follow-up  Baseline data  Participants with dementia N=1547  Carers N=1283 |  |  |  | Costs in 2014/15 GBP    Mean costs of unpaid care by:  Gender  Male: £3107 (2759, 3455)  Female: £2639 (2273, 3006)  Age:  65-69: £2357 (1798, 2915)  70-74: £ 2699 (2168, 3231)  75-79: £2902 (2427, 3378)  85+: £ 3084 (2631, 3537)  Carer status:  Spouse/partner: £3052 (2745, 3359)  Family/friend: £3645 (2654, 4637)  No carer: £ 1461 (1050, 1871)  Household status  Lives w/others: £3333 (3003, 3662)  Lives alone: £ 1033 (724, 1342)  Income:  First quintile (lowest): £3375 (2754, 3996)  Second quintile: £ 3025 (2413, 3637)  Third quintile: £ 3185 (2497, 3873)  Fourth quintile: £2632 (2109, 3155)  Fifth quintile: £ 2294 (1728, 2859) |
| Henderson 2022 (jointly with Henderson 2019)  (3 waves)  4 year follow-up (2014-18)  N=1537 wave 1; 1175 wave 2; 846 wave 3 |  |  | Service use  People without a caregiver: proportion receiving home care consistent over the 3 waves (18%, 22%, 21%); proportion using day centres (9%, 11%, 13%). Very few home admissions (temporary or permanent) in any wave.    People with a caregiver: proportion receiving home care across the 3 waves: 10%, 16%, 20% respectively; proportion using day centres (13%, 17%, 20%). Residential or nursing home stays: 1%, 6%, 10%.    Assistance with activities, participants without a caregiver (N=851/1183 at 24 months):  Wave 2, 53% (n=100) reported receiving assistance of appx 20hrs/week; Wave 3, 48% (n=66) reported appx. 18 hrs/week.    Participants with a caregiver:  Wave 1, 39hrs/week (1009 recipients of care); Wave 2, 48 hrs/week (787 recipients); Wave 3, 56 hrs/week (628 recipients).  Adjusted for age, sex, living alone, caregiver status, dementia sub-type | Costs of unpaid and paid care in 2014/15 GBP  Participants or their families paying for all of the participants homecare  Participants without a caregiver: At Wave 1, 23/43 (53%), Waves 2, 63% (N= 22/35), Wave 3 48% (N= 11/23).  Participants with a caregiver: At Wave 1, 53% (N= 61/115); Wave 2, 53% (N= 77/145); Wave 3, 56% (N= 83/149).    Unpaid care, all carers, mean weighted costs of care during the prior 3 months:  wave 1: £3,318, 95% CI, 95% CI £3068 to £3568; wave 2: £4,215, 95% CI £3,918 to £4,513) wave 3: £4981, 95% CI £4,619 to £5,342    An additional year of age was associated with an 0.8% increase in costs: ExpB 1.008 (95% CI 1.002, 1.015) p=0.013  Female ExpB 1.070 (95% CI 0.945 to 1.111)  Lives alone ExpB 0.969 (95% CI 0.778, 1.208)  Female (ref male) 1.070 (0.945, 1.211)  No caregiver ExpB 0.876 (95% CI 0.692 to 1.110)  Wave 3 data, N=846  (Table 5, page 146)  * Significant heterogeneity in the level and trajectories of the cost of care. |
| Sabatini 2024  (jointly with Henderson 2019 and 2022)  (3 waves)  N=1545 wave 1; 1183 wave 2; 851 wave 3 |  |  | Five percent of people with dementia (n = 77) moved into care over two years (n = 39 at 12-months, n = 38 at 24-months).  Associations with risk of moving into a care home over two years  Table 2 model 3 in Sabatini (self-rated functional ability, N=851/1545 (NB authors also report informant and care-giver self-rated measures N=759/1305)  Age (ref >80 years):  Age 65 to 69 years RR 0.35 (95% CI 0.15 to 0.82)  Age 70 to 74 years RR 0.28 (95% CI 0.13 to 0.63)  Age 75 to 79 years, RR 0.50 (95% CI 0.29 to 0.89)  Female (ref male) RR 0.63 (95% CI 0.39 to 1.01)  Type of dementia  Alzheimer’s Disease (ref):  Vascular dementia RR 1.38 (95% CI 0.65 to 2.89)  A mix of Alzheimer’s Disease/ vascular RR 1.12 (95% CI 0.63 to 1.97)  Frontotemporal dementia RR 1.60 (95% CI 0.36 to 7.24)  Parkinson’s disease dementia/ dementia with Lewy bodies RR 3.20 (95% CI 1.43 to 7.77)  Unspecified/other dementia, poorer cognition RR 1.08 (95% CI 0.24 to 4.76)    Self-rated functional ability (functional activities questionnaire, range 0 to 33) higher scores=poorer functional ability)  RR 1.05 (95% CI 1.01 to 1.08) |  |
| Hu 2022  ELSA  6 years follow-up study  (waves 6 to 9, 2012-2018)  N= 5,685/13,425 aged >65 years living in the community; 1,056 without care needs excluded  4,629/5,685 in wave 6 who developed care needs between waves 6 to 9 included in the analysis; 2,227/4,629 in wave 9  Dual trajectory analyses of care needs and use of unpaid informal or formal care in the home  Long-term need defined as difficulty with 1 or more of 6 ADLs, 4 IADLs and 3 mobility tasks; combined need with intensity of care utilization based on hours of paid formal or unpaid informal care to analyse need and use trajectories | Association between predictors in wave 6 and need and utilization trajectories, results from logistic regression (table 3, page 1371):  Low needs/low intensity use trajectory vs medium needs/ medium intensity use trajectory):  Female OR 1.35, SE 0.1, p<0.001  Ethnic minority OR 2.02 SE 0.47, p<0.01  Married OR 0.83, SE 0.06, p<0.05  Living with children OR 1.05, SE 0.12, NS  Secondary education OR 0.93, SE 0.08, NS  Higher education OR 0.80, SE 0.08, p<0.05  Occupation managerial professional OR 0.87, SE 0.09, NS  Occupation intermediate job OR 1.31, SE 0.12, p<0.001  Wealth OR 0.76, SE 0.08, p<0.05, Housing problems OR 1.71, SE 0.05, p<0.001  Living in Southern England OR 0.82, SE 0.07, p<0.05  Living in the Midlands OR 1.03, SE 0.1 NS  High needs/medium intensity use vs high needs/high intensity use trajectory:  Female OR 1.30, SE 0.29 (NS)  Ethnic minority OR 1.58, SE 0.92 (NS)  Married OR 3.57 SE 0.84, p<0.001  Living with children OR 1.75, SE 0.56, NS  Secondary education OR 1.56, SE 0.45  Higher education OR 1.25, SE 0.43  Occupation managerial professional OR 0.66, SE 0.22 NS  Occupation intermediate OR 1.29, SE 0.4 NS  Wealth OR 2.40, SE 1.52 NS  Housing problems OR 1.04, SE 0.12, NS  Living in the Midlands OR 0.61, SE 0.18  Living in Southern England OR 0.39 SE 0.11, p<0.01  Adjusted for demographic, socio-economic and environmental factors |  |  |  |
| Hu 2024  Cross-sectional  Concentration Index to calculate socio-economic inequality in the use of care.  n/a  N=16,458 analysed; 16,046 no dementia diagnosis, 412 had a diagnosis of dementia (Table 1, page 1607) |  |  | 70% people with dementia received informal care vs 18% with no dementia; 32% with dementia received formal care vs 7% without dementia    Wealth based corrected concentration index (CCI) for informal care (NS=non-significant), total sample CI -0.186 (SE 0.007) p<0.001 N=16,458, group with no dementia CI -0.177 (SE 0.007) p<0.001 N=16,046, group with dementia: CI -0.043 (SE 0.056) NS N=412    Wealth based CCI for formal care, total sample CI -0.052 (SE 0.005) p<0.001 N=16,458, group with no dementia CI -0.042 (SE 0.005) p<0.001 N=16,046, group with dementia: CI -0.222 (0.052) p<0.001 N=412  Income-based CCI for informal care, total sample -0.103 (SE 0.007) p<0.001 N=16,458, group with no dementia CI -0.096 (SE 0.007) p<0.001 N=16,046, group with dementia CI -0.026 (SE 0.052) NS N=412  Income based CCI for formal care, total sample CI 0.024 (SE 0.005) p<0.001 N=16,458, group with no dementia CI -0.017 (SE 0.005) p<0.001 N=16,046, group with dementia CI –0.116 (SE 0.053), p<0.05 N=412  Unmet activities of daily living care needs: wealth based: total sample CCI -0.054 (SE 0.023) p<0.05  Corrected for need and non-need factors |  |
| Iparraguirre 2020  ELSA  8 years follow-up  2004/05 to 2012/2013  N=1,695 wave 2, N=1,530 wave 3, N=1,762 wave 4, N=1,624 wave 5, N=1,737 wave 6 | Unmet need for care (difficulty with one or more ADLs/IALDs and did not receive help or the help received did not meet their needs all of the time)  Age coef 0.033 (SE 0.005) 95% CI 0.024 to 0.041; OR 1.03, 95% CI 0.88 to 1.01  Gender coef 0.003 (SE 0.058), 95% CI -0.111 to 0.116); OR 1.003, 95% CI 0.895 to 1.123  Non-housing wealth coef 8.2E-0.08 (SE1.8E-07), 95% CI -2.7E-07 to 4.4E-07; OR 1.0, 95% CI 1.00 to 1.00  Rural vs urban with major conurbations coef -0.05 (SE 0.24), 95% CI -0.52 to 0.42; OR 0.95, 95% CI 0.59 to 1.53  N=4,058  Adjusted for age, sex, living alone, household characteristics, income, non-housing wealth, local authority area average rankings for income deprivation, rural/urban, type of council with responsibility for adult social care. |  |  |  |
| Kasteridis 2015  Cross-sectional  Follow-up n/a  96,005 Somerset population,1,458 with 3+ chronic conditions measured by United Health’s (U.K) RISC tool, a patient level predictive modelling tool. |  |  |  | Results from multiple regression models. In part one, logistic regression was used to estimate the probability of incurring any expenditure. In part two, the amount of expenditure for those with positive costs was estimated. Primary care costs and total costs for the 3+ group had a small proportion of zeros and were estimated with a single model.  Costs in 2012-13 GBP  In the population with 3+ long-term conditions N=1,458  Co-efficient from generalized linear regression model for community care costs:  Age ≥55: 0.009 NS  Male: -0.371, p<0.001  Deprivation 0.002 NS  Number of MLTC: -0.009 NS  GLM regression model: R^2^ 0.113 for social care  Table 5, page 9  Average annual cost of the people in the 3+ chronic conditions group that utilized social care: 5622 GBP (Table 5)  Adjusted for age, sex, deprivation |
| Knapp 2016  Secondary analysis of electronic health records  6 months follow-up  N=3075  Patients with multiple MMSE scores  could contribute to more than one overlapping follow-up  period if assessments  were more than 6 months apart, the statistical  analyses assessed the impact of repeat observations  For the 3075, there were 5624 included ‘observation windows’ for the outcomes care home admission |  |  | During the 6-month study ‘windows’ examined 361 patients had a care home admission.  Results from logistic regression for care home admission at 6 months follow-up:  Age: OR 1.04, 95% CI 1.02 to 1.06  Male/female (ref female) OR 1.11, 95% 0.85 to 1.44  Living alone RR 1.29, 95% CI 0.99 to 1.67  Ethnicity (ref: white)  Caribbean/African OR 0.57, 95% CI 0.38 to 0.86  East/South Asian OR 0.57, 95% CI 0.24 to 1.34  Mixed/unknown OR 0.30, 95% CI 0.12 to 0.74  Living alone OR 1.29, 95% CI 0.99 to 1.67  Minor problematic living conditions: OR 1.59, 95% CI 1.19 to 2.12  Significant problems with living conditions OR 1.65, 95% CI 1.18 to 2.32  Significant ADL problems OR 1.87, 95% CI 1.21 to 2.90  Minor problems with agitation OR 1.41, 95% CI 1.05 to 1.89  Significant problems with agitation OR 1.98, 95% CI 1.45 to 2.70  Depression significant problems OR 1.13, 95% CI 0.79 to 1.60  Physical illness significant problems OR 1.87, 95% CI 1.23 to 2.90  Prior 12m general hospital inpatient care OR 1.54, 95% CI 1.22 to 1.94  Prior 12m mental health inpatient care OR 2.59, 95% CI: 1.42 to 4.74  Adjusted for age, sex, ethnicity, living alone, household characteristics, living conditions, LTCs, agitation, depression, year of data collection, ADLs. | Generalised linear model of associated costs for sub-sample who had a care home admission N=361  2010/11 prices GBP  All co-variates listed in the previous column were NS, with the exception of living alone: Exp (b) 0.84, 95% CI 0.74 to 0.97, p<0.01 (Table 2)  Average cost estimates average marginal effects (AME) GBP for care home  Mini-mental state examination 30 items  (MMSE) 6 (severe): AME cost: 10,172 (bootstrapped estimated cost: 059)  MMSE 16 (moderate): AME cost: 9782 (bootstrapped estimated cost: 784)  MMSE 24 (mild): AME cost: 9424 (bootstrapped estimated cost: 325) |
| Lennox Smith 2016  Prospective cohort  18 months follow-up study  N=526 |  |  | Care giver time from baseline to 18 months follow-up increased from: mild dementia 156.5 to 220 hours a month; moderate dementia 215 to 272 hours a month; moderately severe/severe 348 to 396 hours a month.  Kaplan-Meier probability of being institutionalised at 18 months:  Mild AD Dementia: 9.0, 95% CI 5.6 to 14.3  Moderate AD Dementia: 17.9, 95% CI 12.7 to 24.7  Moderately severe/Severe Dementia: 28.0, 95% CI 21.0 to 36.7  Overall: 17.2, 95% CI 14.1 to 21.0  No adjustments | Mean total societal cost per patient over 18 months (2010 values):  Patients with moderately severe/severe AD dementia: £43,560 (95% CI: £39,059–£48,481);  Patients with moderate dementia: £30,905 (£28,539–£33,371);  Patients with mild dementia: £25,865 (£23,444–£28,538);  Informal caregiver cost represented over 50% of costs for each severity group (no cost breakdown provided)  No adjustments |
| MacLeod 2021  Cross-sectional  n/a  N=3593 full sample  N=3153 included in the analysis |  |  | Odds ratios (95% CIs) for self-reported use of social care in the 4 weeks prior to baseline regressed on health (n=3153)  Self-rated health:  Excellent: reference  Good: OR 0.74 (95% CI: 0.41 to 1.32)  Fair: OR 0.81 (95% CI: 0.44 to 1.50)  Poor: OR 0.66 (95% CI: 0.30 to 1.43)  Cognitive impairment defined by a Mini-Mental State Examination Score <26 or a study diagnosis of dementia indicated by the Geriatric Mental State - Automated Geriatric Examination for Computer-Assisted Taxonomy algorithm or as assessed by a medically qualified member of the study team through review of participant and informant interviews, and interviewer vignettes.  No impairment: reference  Impairment: OR 1.90 (95% CI: 1.28 to 2.83)  Activities of daily living (categorised as yes or disability with IADL, or ADL and IADL)  No disability (reference)  IADL disability: OR: 4.83 (95% CI 2.69 to 8.67)  ADL-IADL disability: OR 14.85 (95% CI: 8.15 to 7.04)  (Table 3 model 4)  Adjusted for age, sex, socio-economic status measured by occupation, marital status, accommodation type, social engagement, deprivation, urban/rural, service access. |  |
| Nakubulwa 2022  Retrospective cohort  12 months follow-up  N=13,394 |  |  | At 12-month follow-up: 223 (1.7%) used long-term adult social care (ASC) for at least part of the 12-month follow-up; 62.3% were female compared with 54.5% among those not receiving long-term ASC (p=0.024). Long-term ASC included all local authority funded long-term care in the home and residential care, or arranged by the local authority and paid for by the client.  Risk factors associated with needing future long-term social care [supplementary file]  Age  Aged 80-84 (ref 75-79):  OR 1.28, 95% CI 0.90 to 1.81  Aged ≥85 (ref 75-79)  OR 2.17, 95% CI 1.55 to 3.04  Sex [supplementary file]  Female OR 1.25, 95% CI 0.94 to 1.67  Ethnicity [supplementary file]  Mixed 1.16, 95% CI 0.77 to 1.72  Asian/British Asian OR 1.28, 95% CI 0.77 to 2.03  Black/Black British OR 1.31, 95% CI 0.79 to 2.10  IMD (ref 1^st^ quintile, most deprivation)  2^nd^ quintile OR 0.72, 95% CI 0.51 to 1.03  3^rd^ quintile OR 0.61, 95% CI 0.42 to 0.88  4^th^ -5^th^ quintile (least deprived) OR 0.37, 95% CI 0.23 to 0.56    Living alone (ref does not live alone)  OR 1.35, 95% CI 0.69 to 2.98  Long-term conditions:  mental health OR 1.76, 95% CI 1.27 to 2.40  neurological condition OR 2.11, 95% CI 1.41 to 3.06  cardiovascular OR 0.79, 95% CI 0.60 to 1.06  respiratory disease OR 0.85, 95% CI 0.44 to 1.49  malignant disease OR 0.64, 95% CI 0.36 to 1.06  neurological disease OR 2.11, 95% CI 1.41 to 3.06  endocrine disease OR 1.40, 95% CI 0.70 to 2.97  Adjusted for age, sex, living with a partner, age completed education |  |
| Nikolova 2022  Cross-sectional  n/a  ELSA: N= 2,408 CARE75+ N=1,038 |  |  |  | ELSA social care use costed included self-reported use of home care, lunch clubs, day centres, housing manager, meals on wheels, frozen meals, council handyman.  CARE75+ social care use costed included self-reported help from a warden, home care, night sitter and day sitter.  Mean social care costs  For the non-frail: £321 (ELSA: £195, CARE75: £800) (2012 to 2018)  For the frail: £2895 (ELSA: £3220, CARE75: £4336)    For every 1% of non-frail people not transitioning to frailty from an effective intervention, annual savings of £4.4 million (ELSA data) and £4.8 million (CARE data) on formal social care in people’s home paid for by the local authority and/or privately funded in England; estimates exclude expenditure on care homes.  Adjusted for age, sex, living with a partner, age completed education |
| Turner 2015  Secondary analysis of linked administrative data  6 months follow-up  Post-discharge from hospital for residential care,  6 months for patient reported outcome measures not included in this review  N=105,843; results for N=48,146 aged ≥65 years included in the review |  |  | Probability of discharge to an NHS, local authority or non-NHS nursing home: patients who live alone are 0.5% percentage points more likely to be discharged to one of these settings.  Adjusted for age, gender, ethnicity, local-area deprivation, undergoing  revision surgery, or had previous surgery on their hip/knee. All outcomes were adjusted for the measures of baseline health that could plausibly be viewed as independent of the baseline severity of hip/knee problems, namely dummy variables for a number of self-reported comorbidities; discharge to residential care was also adjusted for the month of hospital admission. |  |
| Vlachantoni 2015  Cross-sectional  n/a  N=3395 |  |  | ORs for receiving paid-for-support  Female (male ref)  Female OR 1.63, 95% CI 1.08 to 2.47  Benefit unit equivalized wealth quintile (poorest quintile ref)  Richest quintile OR 3.27 (95% CI 1.54 to 6.81)  Self-report limiting long-term illness (no limiting long-term illness  OR 2.76 (95% CI 1.71 to 4.49)  Receiving informal support for men:  The strongest predictor of receiving informal support for men: reporting a difficulty with a high number of IADLs (ref = none):  1 IADL difficulty: OR 6.25 (95% CI: 4.12-9.47);  2-4 IADL difficulties: OR 13.43 (95% CI: 7.62-23.68);  5-9 IADL difficulties: OR 31.0 (95% CI: 7.83-122.75).  Other predictors for receiving informal support:  Age 75-79: OR 2.16 (95% CI: 1.24-3.74)  Age 80-84: OR 2.65 (95% CI: 1.50-4.66)  Difficulty with 3-4 mobility activities: OR 2.56 (95% CI: 1.44-4.55)  Difficulty with 5-7 mobility activities: OR 3.92 (95% CI: 2.10-7.36)  Difficulty with 8-10 mobility activities: OR 3.24 (95% CI: 1.31-8.01)  Difficulty with 1 ADL: OR 2.07 (95% CI: 1.35-3.16)  Difficulty with 2-3 ADLs: OR 2.77 (95% CI: 1.48-5.16)  Difficulty with 4-6 ADLs: OR 3.18 (95% CI: 1.05-9.64)  Limiting long-standing illness: OR 2.07 (95% CI: 1.36-3.15)  Receiving informal support for women:  The strongest predictors of receiving informal support for women: reporting a difficulty with a high number of IADLs (ref = none);  1 IADL difficulty OR 4.54 (95% CI: 3.25-6.33);  2-4 IADL difficulties OR 17.58 (95% CI: 11.75-26.31);  5-9 IADL difficulties OR 9.65 (95% CI: 3.65-25.46);  Being diagnosed with dementia (ref = no); yes OR 15.34 (95% CI: 1.32-178.72);  Marital status (ref = single);  Married/civil partnered OR 12.78 ((5% CI 5.54-29.53);  Separated/divorced OR 2.75 (95% CI1.08-7.00);  Widowed OR 3.89 (95% CI 1.71-8.86)  Receiving informal support also associated with:  Children present in the household: OR 1.98 (95% CI: 1.28-3.07)  Two or more errors in orientation and time: OR 1.98 (95% CI: 1.01 to 3.88)  Difficulty with 2 mobility activities: OR 2.07 (95% CI: 1.27 to 3.37)  Difficulty with 3-4 mobility activities: OR 4.07 (95% CI: 2.55 to 6.49)  Difficulty with 5-7 mobility activities: OR 4.86 (95% CI: 2.97to 7.93)  Difficulty with 8-10 mobility activities: OR 6.21 (95% CI: 3.32 to 11.59)  Some difficulty walking a quarter mile: OR 1.76 (95% CI 1.23 to 2.53)  Much difficulty walking a quarter mile: OR 2.25 (95% CI 1.45 to 3.49)  Unable to walk a quarter mile: OR 2.37 (95% CI 1.55 to 3.63)  Adjusted for demographic, socio-economic physical health, mental health, environmental factors |  |
| Vlachantoni 2019  Cross-sectional  n/a  N=5,591 | Prevalence of individuals with unmet need:  Analysis restricted to people with difficulty with ADL (N=1223), IADL (N=1217) and mobility (N=2364). 55% of older individuals with an ADL difficulty reported not receiving any support, compared to 24% of those with an IADL difficulty and 80% of those with a mobility difficulty  ORs for unmet need:  Age: ref 85+ years 65 to 75 years  Help with ADL OR 2.73 (95% CI 1.75 to 4.25)  Help with IADL OR 3.00 (95% CI 1.80 to 4.99)  Help with mobility OR 2.65 (95% CI 1.83 to 3.85)  75 to 84 years  Help with ADL OR 2.15 (95% CI 1.43 to 3.23)  Help with IADL OR 2.20 (95% CI 1.36 to 3.57)  Help with mobility OR 1.91 (95% CI 1.36 to 2.68)  Men (women ref)  Help with ADL OR 1.47 (95% CI 1.07 to 2.02)  Help with IADL OR 1.71 (95% CI 1.19 to 2.43)  Help with mobility OR 2.00 (95% CI 1.49 to 2.67)  Living alone (living in a couple ref)  Help with ADL OR 3.3 (95% CI 1.71 to 6.37)  Help with IADL OR 1.91 (95% CI 0.88 to 4.12)  Help with mobility OR 1.93 (95% CI 1.09 to 3.41)  Limiting long-term illness (no long-term illness ref) self-report  Help with ADL OR 0.61 (95% CI 0.38 to 1.00)  Help with IADL OR 0.65 (95% CI 0.38 to 1.13)  Help with mobility OR 0.59 (95% CI 0.39 to 0.89)  Education (ref high)  Low level of education  Help with ADL OR 1.19 (95% CI 0.67 to 2.12)  Help with IADL OR 1.09 (95% CI 0.56 to 2.12)  Help with mobility OR 1.15 (0.66 to 1.99)  Adjusted for demographic, socio-economic and health factors |  |  |  |
