# Supplementary material for: Social and Health Conditions That Drive the Need and Demand for, Use of and Expenditure on Social Care in the UK: A Systematic Review
Source: Cochrane Evid Synth Methods. 2026 Aug 12;4(5):e70093. doi: 10.1002/cesm.70093 (PMC13489229; doi:10.1002/cesm.70093)
Supplement: Supplementary file 5 [file CESM-4-e70093-s005.docx]

Appendix 3 Excluded studies table

| **No** | **Reference** | **Reasons for exclusion** |
| --- | --- | --- |
| 1 | Adekpedjou R, Léon P, Dewidar O, Al-Zubaidi A, Jbilou J, Kaczorowski J, et al. Effectiveness of interventions to address different types of vulnerabilities in community-dwelling older adults: An umbrella review. Campbell Systematic Reviews. 2023;19(2):e1323. | Topic out of scope |
| 2 | Ahc M. Understanding Social Determinants of Health. Hospital Case Management. 2020;28(10):1-6. | Concept paper |
| 3 | Akehurst J, Giles K, Stronge P. Building workforce capacity and capability for integrated working. International Journal of Integrated Care (IJIC). 2017;17:1-2. | Topic out of scope |
| 4 | Alallawi B, Hastings R, Aabe N. Support Needs and Parent Outcomes in Arab Families of Children with Autism Living in the United Kingdom. Brain Sci. 2022;12(8). | Topic out of scope |
| 5 | Alders P, Deeg DJH, Schut FT. Who will become my co-residents? The role of attractiveness of institutional care in the changing demand for long-term care institutions. Archives of Gerontology & Geriatrics. 2019;81:91-7. | Topic out of scope |
| 6 | Aldridge J. Where are we now? Twenty-five years of research, policy and practice on young carers. Critical Social Policy. 2018;38(1):155-65. | Concept paper |
| 7 | Ashford D, Edwards B, McKay S, King E. Volunteer Coaching for Virtual Care Readiness...21st International Conference on Integrated Care (Virtual), May 1-31, 2021. International Journal of Integrated Care (IJIC). 2022;22:1-2. | Topic out of scope |
| 8 | Atherton IM, Lynch E, Williams AJ, Witham MD. Barriers and Solutions to Linking and Using Health and Social Care Data in Scotland. British Journal of Social Work. 2015;45(5):1614. | Topic out of scope |
| 9 | Badland H, Whitzman C, Lowe M, Davern M, Aye L, Butterworth I, et al. Urban liveability: Emerging lessons from Australia for exploring the potential for indicators to measure the social determinants of health. Social Science & Medicine. 2014;111:64-73. | Wrong setting, and topic out of scope |
| 10 | Ball M, Nanda A. Household Attributes and the Future Demand for Retirement Housing. International Journal of Housing Markets and Analysis. 2013;6(1):45-62. | Topic out of scope |
| 11 | Bardsley M, Billings J, Dixon J, Georghiou T, Lewis G, Steventon A. Predicting who will use intensive social care: case finding tools based on linked health and social care data. Age and ageing. 2011;40:265-70. | Wrong study design |
| 12 | Bardsley M, Georghiou T, Chassin L, Lewis G, Steventon A, Dixon J. Overlap of hospital use and social care in older people in England. Journal of health services research & policy. 2012;17:133-9. | Topic out of scope |
| 13 | Bardsley M. Predicting the future level of social care needs. Community Care. 2010(1821):30. | Wrong study design |
| 14 | Belger M, Haro JM, Reed C, Happich M, Argimon JM, Bruno G, et al. Determinants of time to institutionalisation and related healthcare and societal costs in a community-based cohort of patients with Alzheimer's disease dementia. The European journal of health economics : HEPAC : health economics in prevention and care. 2018;20:343-55. | Not U.K |
| 15 | Björkman M, Jyväkorpi SK, Strandberg TE, Pitkälä KH, Tilvis RS. Sarcopenia Indicators as Predictors of Functional Decline and Need for Care among Older People. Journal of Nutrition, Health & Aging. 2019;23(10):916-22. | Topic out of scope |
| 16 | Bjornestad A, Tysnes O-B, Larsen JP, Alves G. Loss of independence in early Parkinson disease: A 5-year population-based incident cohort study. Neurology. 2016;87(15):1599-606. | Topic out of scope |
| 17 | Bone AE, Gomes B, Etkind SN, Verne J, Murtagh FEM, Evans CJ, et al. What is the impact of population ageing on the future provision of end-of-life care? Population-based projections of place of death. Palliative Medicine. 2018;32(2):329-36. | Topic out of scope |
| 18 | Bosco A, Schneider J, Coleston-Shields DM, Jawahar K, Higgs P, Orrell M. Agency in dementia care: systematic review and meta-ethnography. International Psychogeriatrics. 2019;31(5):627-42. | Topic out of scope |
| 19 | Bremer P, Challis D, Hallberg IR, Leino-Kilpi H, Saks K, Vellas B, et al. Informal and formal care: Substitutes or complements in care for people with dementia? Empirical evidence for 8 European countries. Health Policy. 2017;121(6):613-22. | Topic out of scope |
| 20 | Brimblecombe N, Pickard L, King D, Knapp M. Perceptions of unmet needs for community social care services in England. A comparison of working carers and the people they care for. Health & social care in the community. 2016;25:435-46. | Topic out of scope |
| 21 | Britteon, Philip; Fatimah, Alfariany; Gillibrand, Stephanie; Lau, Yiu-Shing; Anselmi, Laura; Wilson, Paul; Sutton, Matt; Turner, Alex J. The impact of devolution on local health systems: Evidence from Greater Manchester, England | Topic out of scope |
| 22 | Brooks H, Devereux-Fitzgerald A, Richmond L, Caton N, Newton A, Downs J, et al. Adapting a social network intervention for use in secondary mental health services using a collaborative approach with service users, carers/supporters and health professionals in the United Kingdom. BMC Health Services Research. 2022;22(1):1-18. | Topic out of scope |
| 23 | Brownie S, Horstmanshof L, Garbutt R. Factors that impact residents' transition and psychological adjustment to long-term aged care: A systematic literature review. International Journal of Nursing Studies. 2014;51(12):1654-66. | Topic out of scope |
| 24 | Burchardt T, Jones E, Obolenskaya P. Formal and informal long-term care in the community: interlocking or incoherent systems? Journal of Social Policy. 2018;47:479-503. | Topic out of scope |
| 25 | Bürge E, von Gunten A, Berchtold A. Factors favoring a degradation or an improvement in activities of daily living (ADL) performance among nursing home (NH) residents: a survival analysis. Arch Gerontol Geriatr. 2013;56(1):250-7. | Topic out of scope |
| 26 | Buylova Gola A, Morris S, Candy B, Davis S, King M, Kupeli N, et al. Healthcare utilization and monetary costs associated with agitation in UK care home residents with advanced dementia: a prospective cohort study. International Psychogeriatrics. 2020;32(3):359-70. | Topic out of scope |
| 27 | Cardoso T, Oliveira MD, Barbosa-Póvoa A, Nickel S. Modeling the demand for long-term care services under uncertain information. Health Care Manag Sci. 2012;15(4):385-412. | Topic out of scope |
| 28 | Chambers D, Cantrell A, Booth A. Recognition of risk and prevention in safeguarding of children and young people: a mapping review and component analysis of service development interventions aimed at health and social care professionals. BMC Health Services Research. 2021;21(1):1-11. | Wrong population, topic not in scope |
| 29 | Chandola T. Spatial and social determinants of urban health in low-, middle- and high-income countries. Public Health. 2012;126(3):259-61. | Topic out of scope |
| 30 | Chappell NL, Funk LM. Social Capital: Does It Add to the Health Inequalities Debate? Social Indicators Research. 2010;99(3):357-73. | Topic out of scope |
| 31 | Chen Q, Amano T, Park S, Kim B. Home and Community-based Services and Life Satisfaction among Homebound and Poor Older Adults. Journal of Gerontological Social Work. 2019;62(7):708-27. | Topic out of scope |
| 32 | Clare L, Martyr A, Henderson C, Gamble LD, Matthews FE, Quinn C, et al. Living Alone with Mild-To-Moderate Dementia: Findings from the IDEAL Cohort. Journal of Alzheimer's disease : JAD. 2020;78:1207-16. | Topic not in scope, characteristics of those who live alone (does not report social conditions) |
| 33 | Comas-Herrera A, Northey S, Wittenberg R, Knapp M, Bhattacharyya S, Burns A. Future costs of dementia-related long-term care: exploring future scenarios. International Psychogeriatrics 2011; 23(1): 20. | Topic not in scope |
| 34 | Coren E, Hossain R, Pardo Pardo J, Veras MM, Chakraborty K, Harris H, et al. Interventions for promoting reintegration and reducing harmful behaviour and lifestyles in street-connected children and young people. Cochrane Database Syst Rev. 2013(2):Cd009823. | Topic out of scope |
| 35 | Costa N, Wübker A, De Mauléon A, Zwakhalen SMG, Challis D, Leino-Kilpi H, et al. Costs of Care of Agitation Associated With Dementia in 8 European Countries: Results From the RightTimePlaceCare Study. Journal of the American Medical Directors Association. 2018;19(1):95.e1-.e10. | Topic out of scope |
| 36 | Cronqvist A, Sundh K. On collaboration between nurses and social workers in the service of older people living at home. A critical literature review. International Practice Development Journal. 2013;3(2):1-19. | Topic out of scope |
| 37 | Cross R, Greaves CJ, Withall J, Rejeski WJ, Stathi A. Delivery fidelity of the REACT (REtirement in ACTion) physical activity and behaviour maintenance intervention for community dwelling older people with mobility limitations. BMC Public Health. 2022;22(1):1112. | Topic out of scope |
| 38 | Dambha-Miller H, Simpson G, Hobson L, Roderick P, Little P, Everitt H, et al. Integrated primary care and social services for older adults with multimorbidity in England: a scoping review. BMC Geriatr. 2021;21(1):674. | Topic out of scope |
| 39 | de la Maisonneuve C, Martins JO. A Projection Method for Public Health and Long-Term Care Expenditures. 2013. | Topic out of scope |
| 40 | Debski M, Tsampasian V, Haney S, Blakely K, Weston S, Ntatsaki E, et al. Post-COVID-19 syndrome risk factors and further use of health services in East England. PLOS Glob Public Health. 2022;2(11):e0001188. | Topic out of scope |
| 41 | Deokhee Y, Johnston BM, Ryan K, et al. Drivers of care costs and quality in the last 3 months of life among older people receiving palliative care: A multinational mortality follow-back survey across England, Ireland and the United States. Palliative Medicine 2020; 34(4): 513-23. | Topic out of scope |
| 42 | Dermody G, Whitehead L, Wilson G, Glass C. The Role of Virtual Reality in Improving Health Outcomes for Community-Dwelling Older Adults: Systematic Review. J Med Internet Res. 2020;22(6):e17331. | Topic out of scope |
| 43 | Desai S. Will it ever change? The continuing unmet needs of older black and ethnic minority people and social care. Diversity and Equality in Health and Care. 2012;9(2) | Conceptual paper/not a study |
| 44 | Devonport TJ, Ward G, Morrissey H, Burt C, Harris J, Burt S, et al. A Systematic Review of Inequalities in the Mental Health Experiences of Black African, Black Caribbean and Black-mixed UK Populations: Implications for Action. Journal of Racial and Ethnic Health Disparities. 2023;10(4):1669-81. | Topic not in scope |
| 45 | Di Domenico SI, Fournier MA. Socioeconomic Status, Income Inequality, and Health Complaints: A Basic Psychological Needs Perspective. Social Indicators Research. 2014;119(3):1679-97. | Topic out of scope |
| 46 | Diernberger K, Luta X, Bowden J, Fallon M, Droney J, Lemmon E, et al. Healthcare use and costs in the last year of life: a national population data linkage study. BMJ Support Palliat Care. 2021. | Wrong outcomes |
| 47 | Dzingina MD, Reilly CC, Bausewein C, Jolley CJ, Moxham J, McCrone P, et al. Variations in the cost of formal and informal health care for patients with advanced chronic disease and refractory breathlessness: A cross-sectional secondary analysis. Palliative Medicine. 2017;31(4):369. | Wrong outcomes |
| 48 | Emerson E. Health status and health risks of the "hidden majority" of adults with intellectual disability. Intellect Dev Disabil. 2011;49(3):155-65. | Wrong population and does not measure social conditions |
| 49 | Evans S, Hills S, Orme J. Doing More for Less? Developing Sustainable Systems of Social Care in the Context of Climate Change and Public Spending Cuts. British Journal of Social Work. 2012;42(4):744. | Conceptual paper/not a study |
| 50 | Fabbietti P, Santini S, Piccinini F, Giammarchi C, Lamura G. Predictors of Deterioration in Mental Well-Being and Quality of Life among Family Caregivers and Older People with Long-Term Care Needs during the COVID-19 Pandemic. Healthcare (2227-9032). 2024;12(3):383. | Wrong population |
| 51 | Fettes L, Neo J, Ashford S, Higginson IJ, Maddocks M. Trajectories of disability in activities of daily living in advanced cancer or respiratory disease: a systematic review. Disability & Rehabilitation. 2022;44(10):1790-801. | Topic out of scope |
| 52 | Ford J, Knight J, Brittain J, Bentley C, Sowden S, Castro A, et al. Reducing inequality in avoidable emergency admissions: Case studies of local health care systems in England using a realist approach. Journal of Health Services Research & Policy. 2022;27(1):31-40. | Topic out of scope |
| 53 | Forder J. Long-Term Care and Hospital Utilisation by Older People: An Analysis of Substitution Rates. Health Economics 2009; 18(11): 1322-38. | Topic out of scope |
| 54 | Forsman H. Exploring educational pathways over the life course in children with out-of-home care experience: A multi-group path analysis. Children & Youth Services Review. 2020;111:N.PAG-N.PAG. | Paediatric population |
| 55 | Freibott CE, Beaudin E, Frazier B-J, Dias A, Cooper MR. Toward Successful and Sustainable Statewide Screening for Social Determinants of Health: Testing the Interest of Hospitals. Population Health Management. 2021;24(5):567-75. | Wrong setting |
| 56 | Frost R, Avgerinou C, Kalwarowsky S, Mahmood F, Goodman C, Clegg A, et al. Enabling health and maintaining independence for older people at home (HomeHealth trial): a multicentre randomised controlled trial. Lancet. 2023;402 Suppl 1:S42. | Topic out of scope, |
| 57 | Gabrielsson A, Tromans S, Newman H, Triantafyllopoulou P, Hassiotis A, Bassett P, et al. Awareness of social care needs in people with epilepsy and intellectual disability. Epilepsy Behav. 2023;145:109296. | Wrong outcomes |
| 58 | Gale CR, Cooper C, Sayer AA. Prevalence of frailty and disability: findings from the English Longitudinal Study of Ageing. Age and ageing. 2014;44:162-5. | Topic out of scope |
| 59 | Gallagher J, Bolt T, Tamiya N. Advance care planning in the community: factors of influence. BMJ Support Palliat Care. 2020. | Topic out of scope |
| 60 | García-Esquinas E, Ortolá R, Prina M, Stefler D, Rodríguez-Artalejo F, Pastor-Barriuso R. Trajectories of Accumulation of Health Deficits in Older Adults: Are There Variations According to Health Domains? Journal of the American Medical Directors Association. 2019;20(6):710-. | Wrong setting |
| 61 | Garg A, Toy S, Tripodis Y, Silverstein M, Freeman E. Addressing Social Determinants of Health at Well Child Care Visits: A Cluster RCT. Pediatrics. 2015;135(2):e296-304. | Wrong patient population |
| 62 | Garner IW, Burgess AP, Holland CA. Developing and validating the Community-Oriented Frailty Index (COM-FI). Arch Gerontol Geriatr. 2020;91:104232. | Topic out of scope |
| 63 | Garner IW, Holland CA. Age-friendliness of living environments from the older person's viewpoint: development of the Age-Friendly Environment Assessment Tool. Age Ageing. 2020;49(2):193-8. | Topic out of scope |
| 64 | Garnett A, Ploeg J, Markle-Reid M, Strachan PH. Formal Health and Social Services That Directly and Indirectly Benefit Stroke Caregivers: A Scoping Review of Access and Use. Canadian Journal of Nursing Research. 2022;54(2):211-33. | Topic out of scope |
| 65 | Gessa GD, Deindl C. Determinants of Trajectories of Informal Caregiving in later life. Evidence from England. Res Sq. 2024. | Wrong population |
| 66 | Gheorghe M, Hoefman RJ, Versteegh MM, van Exel J. Estimating Informal Caregiving Time from Patient EQ-5D Data: The Informal CARE Effect (iCARE) Tool. PharmacoEconomics. 2019;37(1):93-103. | Wrong setting |
| 67 | Giles LC, Hawthorne G, Crotty M, Giles LC, Hawthorne G, Crotty M. Health-related Quality of Life among hospitalized older people awaiting residential aged care. Health & Quality of Life Outcomes. 2009;7:71-. | Wrong population |
| 68 | Gottlieb LMD, Fichtenberg CP, Alderwick H, Adler NP. Social Determinants of Health: What's a Healthcare System to Do? Journal of Healthcare Management. 2019;64(4):243-57. | Conceptual paper/not a study |
| 69 | Gourieroux C, Lu Y. Long Term Care and Longevity. Working Papers 2013 -2016, Centre for Research in Economics and Statistics. [Long Term Care and Longevity](https://ideas.repec.org/p/crs/wpaper/2013-16.html) | Wrong setting |
| 70 | Gray AM. Preventing isolation in sheltered housing: challenges in an era of reduced support funding. Working With Older People. 2017;21(3):186-94. | Topic out of scope |
| 71 | Gupta A, Craig TK. Diet, smoking and cardiovascular risk in schizophrenia in high and low care supported housing. Epidemiol Psichiatr Soc. 2009;18(3):200-7. | Topic out of scope |
| 72 | Gustavsson A, Brinck P, Bergvall N, Kolasa K, Wimo A, Winblad B, et al. Predictors of costs of care in Alzheimer's disease: A multinational sample of 1222 patients. Alzheimer's and Dementia. 2011;7(3):318-27. | Wrong setting |
| 73 | Gustavsson A, Jonsson L, Rapp T, Reynish E, Ousset PJ, Andrieu S, et al. Differences in resource use and costs of dementia care between European countries: Baseline data from the ICTUS study. Journal of Nutrition, Health and Aging. 2010;14(8):648-54. | Wrong setting |
| 74 | Handels RLH, Sköldunger A, Bieber A, Edwards RT, Gonçalves-Pereira M, Hopper L, et al. Quality of Life, Care Resource Use, and Costs of Dementia in 8 European Countries in a Cross-Sectional Cohort of the Actifcare Study. Journal of Alzheimer's Disease. 2018;66(3):1027-40. | Wrong setting |
| 75 | Hanna JR, McConnell T, Harrison C, Patynowska KA, Finucane AM, Hudson B, et al. 'There's something about admitting that you are lonely' - prevalence, impact and solutions to loneliness in terminal illness: An explanatory sequential multi-methods study. Palliat Med. 2022;36(10):1483-92. | Topic out of scope |
| 76 | Haraldsdottir E, Lloyd A, Bijak M, Milton L, Finucane AM. Inpatient hospice admissions. Who is admitted and why: a mixed-method prospective study. Palliat Care Soc Pract. 2023;17:26323524231182724. | Topic out of scope |
| 77 | Hardy R, Boch S, Keedy H, Chisolm D. Social Determinants of Health Needs and Pediatric Health Care Use. Journal of Pediatrics. 2021;238:275-. | Wrong population |
| 78 | Harris JF, Gorman LP, Doshi A, Swope S, Page SD. Development and implementation of health care transition resources for youth with autism spectrum disorders within a primary care medical home. Autism: The International Journal of Research & Practice. 2021;25(3):753-66. | Wrong population |
| 79 | Hasselberg N, Gråwe RW, Johnson S, Ruud T. Treatment and outcomes of crisis resolution teams: a prospective multicentre study. BMC Psychiatry. 2011;11:183. | Topic out of scope |
| 80 | Hautsalo K, Rantanen A, Astedt‐Kurki P. Family functioning, health and social support assessed by aged home care clients and their family members. Journal of Clinical Nursing (John Wiley & Sons, Inc). 2013;22(19-20):2953-63. | Topic out of scope |
| 81 | He Q, Chen X, Zeh P. Promoting Self-care and Management for Patients With Type 2 Diabetes Based on Lifestyle Changes Under the Concept of Person-centered Care: A Review. Altern Ther Health Med. 2023;29(5):302-7. | Wrong population |
| 82 | Hegagi M, James P, Hsu A, Tanuseputro P. Home Care Use and Out-of-Hospital Death in Pancreatic Cancer Patients: A Retrospective Cohort Study. Journal of Palliative Care. 2023;38(2):175-83. | Topic out of scope |
| 83 | Heinonen T, Metteri A, Leach J. Applying health determinants and dimensions in social work practice. European Journal of Social Work. 2009;12(2):139-53. | Conceptual paper |
| 84 | Henderson M, Scourfield J, Sin Yi C, Sharland E. Predictors of Social Service Contact among Teenagers in England. British Journal of Social Work. 2016;46(6):1485-501. | Wrong population |
| 85 | Hollinghurst J, Fry R, Akbari A, Watkins A, Williams N, Hillcoat-Nallétamby S, et al. Do home modifications reduce care home admissions for older people? A matched control evaluation of the Care & Repair Cymru service in Wales. Age and Ageing. 2020;49(6):1056-61. | Topic out of scope |
| 86 | Holman D, Walker A. Social Quality and Health: Examining Individual and Neighbourhood Contextual Effects Using a Multilevel Modelling Approach. Soc Indic Res. 2018;138(1):245-70. | Topic out of scope |
| 87 | Hong S-L, Hasche L, Lee MJ. Service use barriers differentiating care-givers' service use patterns. Ageing & Society. 2011;31(8):1307-29. | Topic out of scope |
| 88 | Humphries R, Thorlby R, Holder H, Hall P, Charles A. Social care for older people: home truths. Social Care for Older People: Home Truths. King’s Fund. 2016 | Concept/discussion paper |
| 89 | Hu, J. Cartagena-Farias, N. Brimblecombe, S. Jadoolal and R. Wittenberg, Projected Costs of Informal care, Eur J Health Econ 2024 Vol. 25 Issue 6 Pages 1057-1070 | Implications and not associations of social conditions (in parallel to health conditions) on care needs |
| 90 | Hunt TN, Roberts K, Taylor EM, Quintana CP, Kossman MK. The Effect of Social Determinants of Health on Clinical Recovery Following Concussion: A Systematic Review. J Sport Rehabil. 2024:1-9. | Wrong population |
| 91 | Ilinca S, Rodrigues R, Fors S, Zólyomi E, Jull J, Rehnberg J, et al. Gender differences in access to community-based care: a longitudinal analysis of widowhood and living arrangements. European Journal of Ageing. 2022;19(4):1339-50. | Wrong setting |
| 92 | Jason LA, Stoolmiller M, Light J. Latent profile analysis in recovery homes: A single quantitative dimension captures most but not all of the important details of the recovery process. Subst Abus. 2022;43(1):666-74. | Wrong population |
| 93 | Jones RW, Lebrec J, Kahle-Wrobleski K, Dell'Agnello G, Bruno G, Vellas B, et al. Disease Progression in Mild Dementia due to Alzheimer Disease in an 18-Month Observational Study (GERAS): The Impact on Costs and Caregiver Outcomes. Dementia and geriatric cognitive disorders extra. 2017;7:87-100. | Topic out of scope |
| 94 | Jönsson L, Tate A, Frisell O, Wimo A. The Costs of Dementia in Europe: An Updated Review and Meta-analysis. PharmacoEconomics. 2023;41(1):59-75. | Wrong setting |
| 95 | Karikari TK, Pascoal TA, Ashton NJ, Janelidze S, Benedet AL, Rodriguez JL, et al. Blood phosphorylated tau 181 as a biomarker for Alzheimer's disease: a diagnostic performance and prediction modelling study using data from four prospective cohorts. Lancet Neurol. 2020;19(5):422-33. | Topic out of scope |
| 96 | Khondoker M, Macgregor A, Bachmann MO, Hornberger M, Fox C, Shepstone L. Multimorbidity pattern and risk of dementia in later life: an 11-year follow-up study using a large community cohort and linked electronic health records. J Epidemiol Community Health. 2023;77(5):285-92. | Wrong outcomes |
| 97 | Kingston A, Forecasting the care needs of the older population in England over the next 20 years: estimates from the Population Ageing and are Simulation (PACSim) modelling study. The Lancet Public Health, 3 (9), e447 to e455 | Implications of associations of social conditions (in parallel to health conditions) on care needs |
| 98 | Kingston A, Wohland P, Wittenberg R, et al. Is late-life dependency increasing or not? A comparison of the Cognitive Function and Ageing Studies (CFAS). Lancet 2017; 390(10103): 1676-84. | Implications and not associations for projections of states of dependency |
|  |  |  |
| 99 | Kojima G, Iliffe S, Walters K. Smoking as a predictor of frailty: a systematic review. BMC Geriatr. 2015;15:131. | Topic out of scope |
| 100 | Kojima G. Quick and Simple FRAIL Scale Predicts Incident Activities of Daily Living (ADL) and Instrumental ADL (IADL) Disabilities: A Systematic Review and Meta-analysis. J Am Med Dir Assoc. 2018;19(12):1063-8. | Wrong outcomes |
| 101 | Lacey J, Moran N. Social Work with No Recourse to Public Fund Migrants: Obstacles and Strategies. British Journal of Social Work. 2024;54(2):607-28. | Wrong population |
| 102 | Lanne M, Leikas J. Ethical AI in the re-ablement of older people: Opportunities and challenges. Gerontechnology. 2021;20(2):1-13. | Topic out of scope |
| 103 | Lara J, Godfrey A, Evans E, Heaven B, Brown LJ, Barron E, et al. Towards measurement of the Healthy Ageing Phenotype in lifestyle-based intervention studies. Maturitas. 2013;76(2):189-99. | Wrong outcomes |
| 104 | Latham RM, Meehan AJ, Arseneault L, Stahl D, Danese A, Fisher HL. Development of an individualized risk calculator for poor functioning in young people victimized during childhood: A longitudinal cohort study. Child Abuse Negl. 2019;98:104188. | Wrong population |
| 105 | Latham RM, Temple RK, Romeo C, Danese A, Fisher HL. Understanding practitioners' and young people's views of a risk calculator for future psychopathology and poor functioning in young people victimised during childhood. J Ment Health. 2021;30(6):706-23. | Wrong population |
| 106 | Lebcir R, Demir E, Ahmad R, Vasilakis C, Southern D. A discrete event simulation model to evaluate the use of community services in the treatment of patients with Parkinson's disease in the United Kingdom. BMC Health Services Research. 2017;17:1-14. | Wrong outcomes |
| 107 | Lem K, McGilton KS, Aelick K, Iaboni A, Babineau J, Hewitt Colborne D, et al. Social connection and physical health outcomes among long-term care home residents: a scoping review. BMC Geriatrics. 2021;21(1):1-10. | Wrong outcomes |
| 108. | Leniz J, Evans CJ, Yi D, Bone AE, Higginson IJ, Sleeman KE. Formal and Informal Costs of Care for People With Dementia Who Experience a Transition to Hospital at the End of Life: A Secondary Data Analysis. Journal of the American Medical Directors Association. 2022;23:2015-22.e5. | Topic out of scope |
| 109 | Lennox-Chhugani N, Welbourn D, Mallender J. A Framework for Assessing Enablers and Risks in Health and Care System Integration. International Journal of Integrated Care (IJIC). 2016;16(6):1-3. | Conceptual paper/not a study |
| 1100 | Li K, Lorgelly P, Jasim S, Morris T, Gomes M. Does a Working Day Keep the Doctor Away? A Critical Review of the Impact of Unemployment and Job Insecurity on Health and Social Care Utilisation. European Journal of Health Economics. 2023;24(2):179-86. | Topic out of scope |
| 111 | Li X, Gao L, Qiu Y, Zhong T, Zheng L, Liu W, et al. Social frailty as a predictor of adverse outcomes among older adults: a systematic review and meta-analysis. Aging Clin Exp Res. 2023;35(7):1417-28. | Wrong outcomes |
| 112 | Li Y, McLernon DJ, Counsell CE, Macleod AD. Incidence and risk factors of institutionalisation in Parkinson's disease and atypical parkinsonism. Parkinsonism Relat Disord. 2024;118:105928. | Topic out of scope |
| 113 | Lindberg-Larsen M, Odgaard A, Fredborg C, Schrøder HM. One-stage versus two-stage revision of the infected knee arthroplasty - a randomized multicenter clinical trial study protocol. BMC Musculoskelet Disord. 2021;22(1):175. | Topic out of scope |
| 114 | Litt JS, Coll-Planas L, Sachs AL, Masó Aguado M, Howarth M. Current Trends and Future Directions in Urban Social Prescribing. Curr Environ Health Rep. 2023;10(4):383-93. | Topic out of scope |
| 115 | Lotfi MS, Shahboulaghi FM, Jablonski RA, Ebadi A, Fadayevatan R, Foroughan M. Facilitators and barriers for families caring for adults living with Alzheimer's dementia: A qualitative study. Geriatr Nurs. 2022;47:61-70. | Wrong study design |
| 116 | Luppa M, Luck T, Weyerer S, König H, Brähler E, Riedel-Heller SG. Prediction of Institutionalization in the Elderly. A Systematic Review. Age and ageing. 2009;39:31-8. | Topic out of scope |
| 117 | Lyu JY, Hu B, Wittenberg R, King D. The relationships between informal and formal social care for older people in England: A comparison before and after the Care Act 2014. Journal of aging & social policy. 2023;36:621-38. | Topic out of scope |
| 118 | MacRae C, Mercer SW, Lawson A, Marshall A, Pearce J, Abubakar E, et al. Impact of individual, household, and area characteristics on health and social care outcomes for people with multimorbidity: Protocol for a multilevel analysis. PLoS One. 2023;18(10):e0282867. | Protocol |
| 119 | Martinussen M, Kaiser S, Adolfsen F, Patras J, Richardsen AM. Reorganisation of healthcare services for children and families: Improving collaboration, service quality, and worker well-being. Journal of Interprofessional Care. 2017;31(4):487-96. | Wrong population |
| 120 | Maschi T, Dasarathy D. Aging With Mental Disorders in the Criminal Justice System: A Content Analysis of the Empirical Literature. Int J Offender Ther Comp Criminol. 2019;63(12):2103-37. | Topic out of scope |
| 121 | Mayrhofer AM, Greenwood N, Smeeton N, Almack K, Buckingham L, Shaheen S, et al. Understanding the financial impact of a diagnosis of young onset dementia on individuals and families in the United Kingdom: Results of an online survey. Health & Social Care in the Community. 2021;29(3):664-71. | Topic out of scope |
| 122 | McCarthy M, McIntyre JC, Nathan R, Ashworth EL, Saini P. Socioeconomic Predictors of Crisis and Clinical Pathways Among People Contacting a Mental Health Crisis Line. Health Serv Insights. 2023;16:11786329231212120. | Topic out of scope |
| 123 | McFeeters S, Pront L, Cuthbertson L, King L. Massage, a complementary therapy effectively promoting the health and well-being of older people in residential care settings: a review of the literature. International Journal of Older People Nursing. 2016;11(4):266-83. | Topic out of scope |
| 124 | McGowan EC, Du N, Hawes K, Tucker R, O'Donnell M, Vohr B, et al. Maternal Mental Health and Neonatal Intensive Care Unit Discharge Readiness in Mothers of Preterm Infants. Journal of Pediatrics. 2017;184:68-74. | Topic out of scope |
| 125 | McGrath-Lone L, Dearden L, Nasim B, Harron K, Gilbert R. Changes in first entry to out-of-home care from 1992 to 2012 among children in England. Child Abuse & Neglect. 2016;51:163. | Wrong population |
| 126 | Meyer AM, Siri G, Becker I, Betz T, Bödecker AW, Robertz JW, et al. The Multidimensional Prognostic Index in general practice: One-year follow-up study. International Journal of Clinical Practice. 2019;73(12):N.PAG-N.PAG. | Topic out of scope |
| 127 | Miranda-Castillo C, Woods B, Orrell M. People with dementia living alone: what are their needs and what kind of support are they receiving? Int Psychogeriatr 2010; 22(4): 607-17. | Wrong study design |
| 128 | Moharra M, Vela E, Dueñas-Espín I, Pauws S, Bescos C, Cano I, et al. Health risk assessment and stratification in an integrated care scenario. International Journal of Integrated Care (IJIC). 2016;16(6):1-3. | Topic out of scope |
| 129 | Mountain G, Wright J, Cooper CL, Lee E, Sprange K, Beresford-Dent J, et al. An intervention to promote self-management, independence and self-efficacy in people with early-stage dementia: the Journeying through Dementia RCT. Health Technol Assess. 2022;26(24):1-152. | Topic out of scope |
| 130 | Needham, L. Vale Community Resource Service: a seven day occupational therapy led reablement service in the Vale of Glamorgan locality...RCOT (Royal College of Occupational Therapist) Annual Conference 2017. British Journal of Occupational Therapy. 2017;80:69-70. | Topic out of scope |
| 131 | Nelson MLA, Saragosa M, Singh H, Yi J. Examining the Role of Third Sector Organization Volunteers in Facilitating Hospital-to-Home Transitions for Older Adults - a Collective Case Study. Int J Integr Care. 2024;24(1):16. | Topic out of scope |
| 132 | Nicholas RS, Heaven ML, Middleton RM, Chevli M, Pulikottil-Jacob R, Jones KH, et al. Personal and societal costs of multiple sclerosis in the UK: A population-based MS Registry study. Mult Scler J Exp Transl Clin. 2020;6(1):2055217320901727. | Topic out of scope |
| 133 | Nilsen KM, Medvene LJ, Ofei-Dodoo S, Smith R, DiLollo A, Graham A, et al. Aging in community: home- and community-based services clients' use of computers as a protective factor for social isolation and loneliness. Educational Gerontology. 2018;44(10):648-61. | Topic out of scope |
| 134 | Niwa H, Ojima T, Watanabe Y, Ide K, Yamato Y, Hoshino H, et al. Association between the 25-question Geriatric Locomotive Function Scale score and the incidence of certified need of care in the long-term care insurance system: The TOEI study. Journal of Orthopaedic Science. 2021;26(4):672-7. | Wrong setting |
| 135 | Norman P, Purdam K. Unpaid Caring Within and Outside the Carer's Home in England and Wales. Population, Space and Place. 2013;19(1):15-31. | Wrong outcomes |
| 136 | Okura M, Ogita M, Yamamoto M, Nakai T, Numata T, Arai H. Self-Assessed Kyphosis and Chewing Disorders Predict Disability and Mortality in Community-Dwelling Older Adults. J Am Med Dir Assoc. 2017;18(6):550.e1-.e6. | Topic out of scope |
| 137 | O'Leary C, Ralphs R, Stevenson J, Smith A, Harrison J, Kiss Z, et al. The effectiveness of abstinence-based and harm reduction-based interventions in reducing problematic substance use in adults who are experiencing homelessness in high income countries: A systematic review and meta-analysis: A systematic review. Campbell Systematic Reviews. 2024;20(2):e1396. | Topic out of scope |
| 138 | O'Shaughnessy Í, Romero-Ortuno R, Edge L, Dillon A, Flynn S, Briggs R, et al. Home FIRsT: interdisciplinary geriatric assessment and disposition outcomes in the Emergency Department. Eur J Intern Med. 2021;85:50-5. | Topic out of scope |
| 139 | Paganini-Hill A. Aging in Place in a Retirement Community: 90+ Year Olds. J Hous Elderly. 2013;27(1-2):191-205. | Wrong setting |
| 140 | Palmer, G. I.; Harper, P.; Knight, V.; Brooks, C. Modelling changes in healthcare demand through geographic data extrapolation | Topic out of scope |
| 141 | Panca M, Livingston G, Barber J, Cooper C, Frenais FL, Marston L, et al. Healthcare resource utilisation and costs of agitation in people with dementia living in care homes in England - The Managing Agitation and Raising QUality of LifE in Dementia (MARQUE) study. PLoS ONE. 2019;14(2). | Topic out of scope |
| 142 | Paquete AT, Martins R, Kotsopoulos N, Urbich M, Green C, Connolly MP. Fiscal Consequences of Alzheimer's Disease and Informal Care Provision in the UK: A "Government Perspective" Microsimulation. Journal of the Economics of Ageing. 2022;23. | Topic out of scope |
| 143 | Pearson M, Hemsley A, Blackwell R, Pegg L, Custerson L. Improving Hospital at Home for frail older people: insights from a quality improvement project to achieve change across regional health and social care sectors. BMC Health Serv Res. 2017;17(1):387. | Topic out of scope |
| 144 | Penzenstadler L, Soares C, Anci E, Molodynski A, Khazaal Y. Effect of Assertive Community Treatment for Patients with Substance Use Disorder: A Systematic Review. Eur Addict Res. 2019;25(2):56-67. | Topic out of scope |
| 145 | Percival A, Newton C, Mulligan K, Petrella RJ, Ashe MC. Systematic review of social prescribing and older adults: where to from here? Fam Med Community Health 2022; 10(Suppl 1). [The Elston study from this systematic review] | Topic out of scope |
| 146 | Pham TM, Petersen I, Walters K, Raine R, Manthorpe J, Mukadam N, et al. Trends in dementia diagnosis rates in UK ethnic groups: analysis of UK primary care data. Clin Epidemiol. 2018;10:949-60. | Topic out of scope |
| 147 | Pickard L. A growing care gap' the supply of unpaid care for older people by their adult children in England to 2032. Ageing and Society. 2015;35(1):96-123. | Topic out of scope - supply |
| 148 | Piirainen K, Piirainen S, Kuha S, Ylisirniö M, Kanste O. Sosiaali- ja terveysalan lähiesihenkilöiden kokemuksia hoitotyön varahenkilöstöstä henkilöstövoimavarojen johtamisen näkökulmasta. Hoitotiede. 2023;35(4):365-81. | Wrong setting |
| 149 | Pilotto A, Aprile PL, Veronese N, Lacorte E, Morganti W, Custodero C, et al. The Italian guideline on comprehensive geriatric assessment (CGA) for the older persons: a collaborative work of 25 Italian Scientific Societies and the National Institute of Health. Aging Clin Exp Res. 2024;36(1):121. | Wrong setting |
| 150 | Poon AWC, Joubert L, Mackinnon A, Harvey C. Recovery for Carers of People with Psychosis: A Longitudinal Population-Based Study with Implications for Social Work. British Journal of Social Work. 2018;48(6):1754-73. | Topic out of scope |
| 151 | Reed C, Belger M, Vellas B, Andrews JS, Argimon JM, Bruno G, et al. Identifying factors of activities of daily living important for cost and caregiver outcomes in Alzheimer's disease. International psychogeriatrics. 2015;28:247-59. | Topic out of scope |
| 152 | Reilly S, Miranda-Castillo C, Malouf R, Hoe J, Toot S, Challis D, et al. Case management approaches to home support for people with dementia. Cochrane Database of Systematic Reviews. 2015(1):N.PAG-N.PAG. | Topic out of scope |
| 153 | Richman EL, Lombardi BM, de Saxe Zerden L, Forte AB. What Do EHRs Tell Us about How We Deploy Health Professionals to Address the Social Determinants of Health. Social Work in Public Health. 2022;37(3):287-96. | Topic out of scope |
| 154 | Rodrigues R, Glendinning C. Choice, Competition and Care - Developments in English Social Care and the Impacts on Providers and Older Users of Home Care Services. Social Policy and Administration. 2015;49(5):649-64. | Topic out of scope |
| 155 | Sandhaug M, Andelic N, Langhammer B, Mygland A. Community integration 2 years after moderate and severe traumatic brain injury. Brain Inj. 2015;29(7-8):915-20. | Topic out of scope |
| 156 | Savvas S, Goh AMY, Batchelor F, Doyle C, Wise E, Tan E, et al. Promoting Independence Through quality dementia Care at Home (PITCH): a research protocol for a stepped-wedge cluster-randomised controlled trial. Trials. 2021;22(1):1-9. | Conceptual paper/not a study |
| 157 | Schaller S, Mauskopf J, Kriza C, Wahlster P, Kolominsky-Rabas PL. The main cost drivers in dementia: a systematic review. Int J Geriatr Psychiatry. 2015;30(2):111-29. | Topic out of scope |
| 158 | Shand J, Gomes M, Morris S. The impact of having a carer on adult health and social care utilisation across five settings of care: A matched cohort study. Health Policy 2023; 129: 104705 | Wrong population |
| 159 | Siaw-Teng L, Ansari S, Jonnagaddala J, Narasimhan P, Ashraf MM, Harris-Roxas B, et al. Use of mHealth for promoting healthy ageing and supporting delivery of age-friendly care services: a systematic review. International Journal of Integrated Care (IJIC). 2019;19(S1):1-2. | Topic out of scope |
| 160 | Simkiss DE, Stallard N, Thorogood M. A systematic literature review of the risk factors associated with children entering public care. Child Care Health Dev. 2013;39(5):628-42. | Topic out of scope |
| 161 | Simon C, Kumar S, Kendrick T. Cohort study of informal carers of first-time stroke survivors: profile of health and social changes in the first year of caregiving. Soc Sci Med. 2009;69(3):404-10. | Wrong population |
| 162 | Sinclair DR, Maharani A, Chandola T, Bower P, Hanratty B, Nazroo J, et al. Frailty among Older Adults and Its Distribution in England. J Frailty Aging. 2022;11(2):163-8. | Wrong outcomes |
| 163 | Singh SP, Islam Z, Brown LJ, Gajwani R, Jasani R, Rabiee F, et al. Programme Grants for Applied Research. Ethnicity, detention and early intervention: reducing inequalities and improving outcomes for black and minority ethnic patients: the ENRICH programme, a mixed-methods study. 2013. | Topic out of scope |
| 164 | Slobbe L, Wong A, Verheij R, Oers H, Polder J, Slobbe LCJ, et al. Determinants of first-time utilization of long-term care services in the Netherlands: an observational record linkage study. BMC Health Services Research. 2017;17(1):1-10. | Wrong setting |
| 165 | Smith J, Ali P, Birks Y, Curtis P, Fairbrother H, Kirk S, et al. Umbrella review of family-focused care interventions supporting families where a family member has a long-term condition. Journal of Advanced Nursing (John Wiley & Sons, Inc). 2020;76(8):1911-23. | Topic out of scope |
| 166 | Smith KJ, George C, Ferriera N. Factors emerging from the "Zarit Burden Interview" and predictive variables in a UK sample of caregivers for people with dementia. Int Psychogeriatr. 2018;30(11):1671-8. | Wrong population |
| 167 | Smith W, McCrone P, Goddard C, Gao W, Burman R, Jackson D, et al. Comparisons of Costs between Black Caribbean and White British Patients with Advanced Multiple Sclerosis in the UK. Mult Scler Int. 2014;2014:613701. | Wrong population |
| 168 | Soysal P, Perera G, Isik AT, Onder G, Petrovic M, Cherubini A, et al. The relationship between polypharmacy and trajectories of cognitive decline in people with dementia: A large representative cohort study. Exp Gerontol. 2019;120:62-7. | Topic out of scope |
| 169 | Steventon A, Roberts A. Estimating length of stay in publicly-funded residential and nursing care homes: a retrospective analysis using linked administrative data sets. BMC Health Serv Res. 2012;12:377. | Wrong outcomes |
| 170 | Steventon A, Roberts A. Estimating Lifetime Costs of Social Care: A Bayesian Approach Using Linked Administrative Datasets from Three Geographical Areas. Health economics. 2014;24:1573-87. | Wrong outcomes (life-time costs for men and women) |
| 171 | Stiefler S, Seibert K, Domhoff D, Görres S, Wolf-Ostermann K, Peschke D. [Influencing factors for nursing home admission in case of pre-existing need of care - a systematic review]. Z Evid Fortbild Qual Gesundhwes 2020; 153-154: 60-75. | Wrong setting |
| 172 | Stokes J, Kristensen SR, Checkland K, Bower P. Effectiveness of multidisciplinary team case management: Direct and spillover effects. International Journal of Integrated Care (IJIC). 2016;16(6):1-3. | Topic out of scope |
| 173 | Sultana J, Fontana A, Giorgianni F, Basile G, Patorno E, Pilotto A, et al. Can information on functional and cognitive status improve short-term mortality risk prediction among community-dwelling older people? A cohort study using a UK primary care database. Clin Epidemiol. 2018;10:31-9. | Wrong outcomes |
| 174 | Swan, L., Warters, A. & O’Sullivan, M. Socioeconomic Disadvantage is Associated with Probable Sarcopenia in Community-Dwelling Older Adults: Findings from the English Longitudinal Study of Ageing. J Frailty Aging 11, 398–406 (2022). https://doi.org/10.14283/jfa.2022.32 | Wrong outcomes |
| 175 | Taylor C, Tsakirides C, Moxon J, Moxon JW, Dudfield M, Witte K, et al. Exercise dose and all-cause mortality within extended cardiac rehabilitation: a cohort study. Open Heart. 2017;4(2):e000623. | Topic out of scope |
| 176 | Tilley E, Jordan J, Larkin M, Vseteckova J, Ryan S, Wallace L. Transitions for older people with intellectual disabilities and behaviours that challenge others: A rapid scoping review. J Appl Res Intellect Disabil. 2023;36(2):207-29. | Topic out of scope |
| 177 | Tsiachristas A, Broad A, Coates A, Singh S, Fossey J. Survival and community care use by care home residents in England: does mental health matter? Ageing and Society. 2021;43:1089-103. | Topic out of scope |
| 178 | Tsiachristas A, Coates A, Broad A, Fossey J. Cost-effectiveness of an Integrated Care Home Support Service in Oxfordshire, England. International Journal of Integrated Care (IJIC). 2017;17:1-2. | Topic out of scope |
| 179 | Urwin S, Lau YS, Grande G, Sutton M. The extent and predictors of discrepancy between provider and recipient reports of informal caregiving. Soc Sci Med. 2021;277:113890. | Wrong outcomes |
| 180 | van Horik JO, Collins R, Martyr A, Henderson C, Jones RW, Knapp M, et al. Limited receipt of support services among people with mild-to-moderate dementia: Findings from the IDEAL cohort. International journal of geriatric psychiatry. 2022;37. | Wrong outcomes |
| 181 | van Lier LI, van der Roest HG, Oosten BS, Garms-Homolová V, Onder G, Finne-Soveri H, et al. Predictors of Societal Costs of Older Care-Dependent Adults Living in the Community in 11 European Countries. Health Serv Insights. 2019;12:1178632918820947. | Wrong setting |
| 182 | Victor CR, Dobbs C, Gilhooly K, Burholt V. Exploring intergenerational, intra-generational and transnational patterns of family caring in minority ethnic communities: the example of England and Wales. International Journal of Care and Caring. 2019;3(1):75. | Wrong population |
| 183 | Vilches-Moraga A, Price A, Braude P, Pearce L, Short R, Verduri A, et al. Increased care at discharge from COVID-19: The association between pre-admission frailty and increased care needs after hospital discharge; a multicentre European observational cohort study. BMC Medicine. 2020;18(1):1-9. | Topic out of scope |
| 184 | Vlachantoni A, Shaw RJ, Willis R, Evandrou M, Falkingham J, Luff R. Measuring unmet need for social care amongst older people. Population Trends. 2011;145:60-76. | Concept paper |
| 185 | Walters K, Frost R, Kharicha K, et al. Home-based health promotion for older people with mild frailty: the HomeHealth intervention development and feasibility RCT. Health Technol Assess 2017; 21(73): 1-128 | Topic out of scope |
| 186 | Weaver RH, Burley M. Identification of Functional, Social, and Health Needs of Older Residents in Nonurban Settings: A Community Risk Assessment. Journal of Aging & Health. 2020;32(10):1614-24. | Wrong setting |
| 187 | Weed HG. 2017 - Preoperative geriatric assessment reduced hospital stay and complications in patients ≥65 y having vascular surgery. ACP Journal Club. 2017;167(2):1-. | Topic out of scope |
| 188 | Wei K, Liu Y, Yang J, Gu N, Cao X, Zhao X, et al. Living arrangement modifies the associations of loneliness with adverse health outcomes in older adults: evidence from the CLHLS. BMC Geriatrics. 2022;22(1):1-11. | Wrong setting |
| 189 | Welch V, Mathew CM, Babelmorad P, Yanfei L, Ghogomu ET, Borg J, et al. Health, social care and technological interventions to improve functional ability of older adults living at home: An evidence and gap map. Campbell Systematic Reviews. 2021;17(3):1-245. | Topic out of scope |
| 190 | Werner S, Auslander GK, Shoval N, Gitlitz T, Landau R, Heinik J, et al. Caregiving burden and out-of-home mobility of cognitively impaired care-recipients based on GPS tracking. International Psychogeriatrics. 2012;24(7):1836-45. | Wrong outcomes, no data on social conditions and social care need/use |
| 191 | Whalley LJ, Staff RT, Lemmon H, Fox HC, McNeil C, Murray AD. Cognitive Test Scores and Progressive Cognitive Decline in the Aberdeen 1921 and 1936 Birth Cohorts. Brain Sci. 2022;12(3). | Topic out of scope |
| 192 | Williams ED, Tillin T, Whincup P, Forouhi NG, Chaturvedi N. Ethnic differences in disability prevalence and their determinants studied over a 20-year period: a cohort study. PLoS One. 2012;7(9):e45602. | Topic out of scope |
| 193 | Wilson P, Wood R, Lykke K, Hauskov Graungaard A, Ertmann RK, Andersen MK, et al. International variation in programmes for assessment of children's neurodevelopment in the community: Understanding disparate approaches to evaluation of motor, social, emotional, behavioural and cognitive function. Scand J Public Health. 2018;46(8):805-16. | Wrong population |
| 194 | Wittenberg |  |
| 195 | Wittenberg |  |
| 196 | Wolff JL, Mulcahy J, Roth DL, Cenzer IS, Kasper JD, Huang J, et al. Long‐Term Nursing Home Entry: A Prognostic Model for Older Adults with a Family or Unpaid Caregiver. Journal of the American Geriatrics Society. 2018;66(10):1887-94. | Wrong setting |
| 197 | Woolham J, Benton C. The Costs and Benefits of Personal Budgets for Older People: Evidence from a Single Local Authority. British Journal of Social Work. 2013;43(8):1472. | Wrong population |
| 198 | Woolham J, Daly G, Hughes E. Loneliness amongst older people: findings from a survey in Coventry, UK. Quality in Ageing and Older Adults. 2013;14(3):192-204. | Wrong outcomes |
| 199 | Wubker A, Zwakhalen SMG, Challis D, Suhonen R, Karlsson S, Zabalegui A. Costs of Care for People with Dementia Just before and after Nursing Home Placement: Primary Data from Eight European Countries. European Journal of Health Economics 2015; 16(7): 689-707. | Wrong outcomes |
| 200 | Xie C, Hughes J, Sutcliffe C, Chester H, Challis D. Promoting Personalization in Social Care Services for Older People. Journal of Gerontological Social Work. 2012;55(3):218. | Topic out of scope |
| 201 | Young J, Hulme C, Smith A, Buckell J, Godfrey M, Holditch C, et al. Health Services and Delivery Research. Measuring and optimising the efficiency of community hospital inpatient care for older people: the MoCHA mixed-methods study. 2020. | Topic out of scope |
| 202 | Zavertnik JE. Self-care in Older Adults With Heart Failure. Clinical Nurse Specialist: The Journal for Advanced Nursing Practice. 2014;28(1):19-32. | Topic out of scope |
| 203 | Zhang W, Rand S, Milne A, Collins G, Silarova B. The quality of life of older carers and the people they support: An international scoping review. Health & Social Care in the Community. 2022;30(6):e3342-e53. | Topic out of scope |
| 204 | Zhou Z, Ryan J, Tonkin AM, Zoungas S, Lacaze P, Wolfe R, et al. Association Between Triglycerides and Risk of Dementia in Community-Dwelling Older Adults: A Prospective Cohort Study. Neurology. 2023;101(22):e2288-e99. | Topic out of scope |
