# Supplementary material for: Social and Health Conditions That Drive the Need and Demand for, Use of and Expenditure on Social Care in the UK: A Systematic Review
Source: Cochrane Evid Synth Methods. 2026 Aug 12;4(5):e70093. doi: 10.1002/cesm.70093 (PMC13489229; doi:10.1002/cesm.70093)
Supplement: Supplementary file 6 [file CESM-4-e70093-s006.docx]

# Table S1 – Characteristics of Included Studies

| ***Author and study aim*** | ***Geographical Setting*** | ***Study design*** | ***Data source*** | ***Study population*** | ***Ethnicity*** | ***Age*** | ***Sex*** | ***Education*** |
| --- | --- | --- | --- | --- | --- | --- | --- | --- |
| **Burton 2018**  **Aim**: to describe the association between social demographic, clinical factors and long-term admission to a care home | Scotland | Prospective cohort; 3 year follow-up.  The research team combined the intervention and control groups from a randomised trial | PROSPER linked to national routine data using the Scottish Community Health Index; recruitment 1997 to 1999, follow-up 2012 to 2015; [WOSCOPS excluded due to study population age] | Primary prevention, elevated CV risk for secondary prevention  N=2033 | NR | PROSPER mean age 75.8 (SD 3.3) | Female  N=1155, 57% | NR |
| **Chukwusa 2022**  **Aim:** to understand the association and prevalence of MLTCs, adjusted for socio-demographic factors, with expenditure on social care in England | England | Cross-sectional population based local authority area level analysis | ONS death registry 2018 data    NHS Digital Adult Social Care Activity and Finance report (2018-19) to calculate expenditure on adult social care, data were aggregate expenditure | Older people aged 65 years and above who died in 2018. Underlying causes of death included: cancers, cerebrovascular disease, CVD, neurological conditions. Contributing causes of death were used as a proxy for MLTCs, and included: chronic respiratory disease, diabetes, CVDs, dementia, stroke, and CKDs as a secondary cause of death recorded on their death certificate  N=409,551 | NR | Mean: 83.57 (SD 8.18) | Female: 216,120 (52.8%)  Male: 193,431 (47.2%) | NR |
| **Davies 2022**  **Aim:** To investigate the influence of multi-morbidity on (a) transitions in mobility disability and (b) mobility disability-free life expectancy | England | A population-based prospective cohort study (the Newcastle 85+ Study) of community-dwelling and institutionalised individuals who were born in 1921; 10-year follow-up | Newcastle 85+ cohort; recruited in 2006 | A population aged 85 years in 2006 who were registered with a General Practice in Newcastle or North Tyneside. Excluded people with end-stage terminal illness  N=845 | Details NR but acknowledges limited ethnic diversity in the Newcastle 85% study. | 85 years in 2006  95 years in 2016 | F 62% (N=524)  M 38% (N=321) | Mean years of education: 9.91 (SD 1.86) |
| **Dunatchik 2019 & Dunatchik 2016**  **Aim:** to predict unmet care needs | England | English Longitudinal Survey of Ageing;^1^ 10-year follow-up | ELSA), wave 1, 2002 to wave 6, 2012 | Data from waves 1-6; includes respondents without ADL difficulties at wave 1 (2002) and with difficulties (1 ADL and/or 2 or more IADLs or mobility difficulties) at wave 6 (2012), who were aged 60 or over at wave 6.  Study sample N=1,082 | NR | 49-54: 14%  55-59: 19%  60-64: 15%  65-69: 16%  70-74: 21%  75+: 15%  Reference group aged 49 to 54.  Analysis based on 55 to 75 + years | F 59%  M 41% | Data from wave 1: 59% no education  31% medium education, 10% high education |
| **Gousia 2020**  **Aim:** to estimate the prevalence of unmet social care needs of people over 50 living in England with cancer and the effect of cancer on unmet needs | England | Cross-sectional | Pooled data from ELSA, all 8 waves 2002/03 to 2016/17 | N=81,338 total sample  N= 4,767 people living with and beyond cancer (LWBC)  N=76,571 without cancer | Full sample non-white 0.03 (0.18); people living with and beyond cancer: non‐white 0.02 (0.14) | Full sample: mean 65.8 (SD 10.4) | Full sample mean (SD): M 0.44 (0.49) | Total sample:  Less than O-level: 0.45 (0.49)  O-level: 0.26 (0.44)  Higher than A-level: 0.29 (0.45) |
| **Henderson 2019 (baseline data)**  **Henderson 2022 (3 waves)**  **Aim:** To characterize use and costs of paid and unpaid care over time in a cohort of people with dementia living in Britain. To explore the relationship between demographic and clinical characteristics and service costs**.**  **Sabatini 2024 reporting additional results (3 waves)**  To assess predictors of moving to a care home | England, Scotland, Wales | Prospective cohort, 4- year follow-up | Improving the Experience of Dementia and Enhancing Active Life (IDEAL) programme, waves 1 to 3, 2014 to 2018 | Participants from England (90%), Wales (5%), and Scotland (5%); living in the community; have clinical diagnosis of mild to moderate dementia; score Mini-Mental State Examination ≥15 and their primary caregivers who provide unpaid support, when available.  N=1537 wave 1; 1175 wave 2; 846 wave 3 | NR | For all 3 waves: mean age: 76 | For all 3 waves: % female 43-44% | Baseline (Henderson 2019)  No qualification n=430 (28%)  School certificate age 16: n= 274 (18%)  School certificate age 18: n= 529 (34%)  College-level: n=314 (20%) |
| **Hu 2022**  Aim: to cluster the trajectories of low, medium and high long-term care needs and low, medium and high care utilisation in older people aged 65 years and over in England | England | English Longitudinal Survey of Ageing;^1^ 6-year follow-up | ELSA waves 6–9, 2012–2018 | Community-dwelling 65+ year olds in wave 6, who developed care needs in at least one wave.  Sample size across waves 6 to 9 of ELSA data N=13,425  Sample size of population >65 years included in the analysis and who developed care needs in at least one wave of data N=4,629 | White: 97.7% (n=4522); ethnic minority 2.3% (n=106) | All participants 65+ year old. Sample age mean: 75. | Male: 43.7% (n=2022); female 56.3% (2607) | No qualifications: 48.5% (2208); secondary education 26.2% (1189); higher education 25.3% (1153) |
| **Hu 2024**  **Aim:** To investigate socioeconomic  inequality and inequity in the utilisation of long-term care for older people with and without dementia in England. | England | Cross-sectional | Pooled ELSA waves 6, 7 and 8, 2012, 2014, 2016 | Pooled sample of older people living in the community (not a care home) aged ≥65 years selected from ELSA with and without dementia  N=16,458 analysed; 16,046 no diagnosis of dementia, 412 had a diagnosis of dementia | NR | ≥65 years  Group without dementia: mean 73.9  Group with dementia: mean age 80.0 years | Group without dementia  M 46%  F 54%  Group with dementia  M 47%  F 53% | Higher education: group with no dementia 29%.  group with dementia 22%,  Secondary education: group with no dementia 30%, group with dementia 25%;  Primary education: group with no dementia 41%, group with dementia 53%, |
| **Iparraguire 2020**  **Aim:** do funding levels affect unmet needs irrespective of whether any help was provided and, if it was, who provided and who funded the service; used multi-level model cross classified logistic regression that included co-variates relevant to this review | England | English Longitudinal Survey of Ageing;^1^ 8-year follow-up | ELSA (wave 2, 2004/2005 to wave 6, 2012/2013)  Council with Adult Social Services  Responsibility (CASSR) data  2015/16 prices | Living in private households in England  N=4,058 | NR | ≥65 years  Mean age: from 76.74 | Unclear | NR |
| **Kasteridis 2015**  **Aim:** To identify which groups of the South Somerset population in England would most benefit from greater integration across primary, community, acute and social care settings | England | Cross-sectional | Linked health and social care data for the South Somerset population for the financial year 2012/2013 | Subgroup of South Somerset population with 3+ chronic conditions  N=1,458 | NR | Mean 77.5 years | M: 53% | NR |
| **Knapp 2016**  **Aim:** To examine the links between the clinical and other characteristics of people with Alzheimer’s disease living in the community and both the likelihood of admission to a care home or hospital  within a 6-month period, and the associated costs of those admissions. | England | Secondary analysis of Electronic Health Records; 6-month follow-up. | Electronic health records of the South London and Maudsley National Health Service (NHS) Foundation Trust (SLAM) that used a  Clinical Record Interactive Search (CRIS) system to retrieve de-identified clinical records, linked to Hospital Episode Statistics. Data available from 2008, extracted in 2012. | All individuals in the CRIS system with a diagnosis of Alzheimer disease (AD) from structured ICS10 and GATE data, and at least one Mini-Mental State Examination (MMSE) rating, not resident in a care home at the time of the MMSE assessment.  N=3,075 | White 2529 (82.2%)  Caribbean/African 310 (10.1%)  East/South Asian 96 (3.1%)  Mixed/unknown 140 (4.6%) | Number reported in age bands:  40–59 years 43 (1.4%)  60–69 years 207 (6.7%)  70–79 years 1049 (34.1%); 80–89 years 1486 (48.3%); 90 years or above 289 (9.4%) | Female 2059 (67%)  Male 1013 (33%) | NR |
| **Lenox-Smith 2016**  **Aim:** to estimate clinical outcomes and costs for people with mild, moderate and severe AD dementia in U.K, using national data on resource use and costs | United Kingdom | Prospective cohort;18-month follow-up | GERAS, a prospective observational multi-centre European study | Community dwelling patients; aged at least 55 years, presenting within  the normal course of clinical care; diagnosed with probable  AD dementia  N=526 | NR | Mean: 78.5 years (SD 7.8) | F 54.2% (n=285) | NR |
| **MacLeod 2021**  **Aim:** to model the relationship between self-rated health, cognitive impairment and ADLs) | Wales | Cross-sectional | Cognitive Function and Ageing Study Wales (CFAS Wales) wave 1 & 2; participants selected from primary care lists from 2011 to 2013 | Older population: 25% with cognitive impairment, 75% without impairment  N=3,593 (full sample)  N=3,153 (analysis sample) | 99% white (full sample), the same as the ethnic profile of over 65s in Wales | ≥65 years  Analysis sample mean age 74 years (SD 7 years, range 65-102) | Analysis sample:  M 1486 (47%),  F 1667 (53%) | NR |
| **Nakubulwa 2022**  **Aim:** to describe risk factors for long-term adult social care (ASC) in two inner London boroughs and develop a risk prediction model for long-term ASC | England | Retrospective cohort; 1 year follow-up | Pseudo-anonymised person-level data from an integrated care dataset (Northwest London Discover Database) that links data on primary, secondary and tertiary care, community mental health care, emergency departments and social care in 2 NW London boroughs covering a population of 2.3 million; 2012 to 2018 | Registered with a GP and resident in either one of two NWL inner boroughs; aged 75 or older at the start of the follow up period; not have accessed long-term or short-term ASC in the 6 year period to 31/12/2018  N=13,394 | White 9678 (72.3%), mixed other ethnic groups 1,776 (13.3%), Asian/Asian British 1,029 (7.7%), Black/Black British 911 (6.8%) | >75 years  75-79: n=6196 (46.3%)  80-84: n=4133 (30.9%)  85+: n=3065 (22.9%) | F 7,322 (54.7%)  M 6,072 (45.3%) | NR |
| **Nikolova 2022**  **Aim:** to examine the relationship between community-dwelling older people living with frailty and annual formal social care costs for the 2012–2018 period. | England | Cross-sectional | Community Ageing Research 75+ (CARE75+ 2018) ELSA (wave 6 data, 2012-13) and ONS population data to estimate the number becoming frail by age category | CARE75+ data: community-dwelling older people aged 75 and over, with wide geographical representation across urban and rural areas of the country;  ELSA wave 6\; representative of adults aged 50+ in England  ELSA: N= 2,408 CARE75+ N=1,038 | NR | CARE75 mean: 81.5 (SD 4.9)  ELSA mean: 80.6 (SD 4.6) | CARE75:  F 52%  ELSA:  F 56% | Age at finishing education:  Education in years  CARES 75+: <=14: 18%;  15: 33%;  16: 20%;  17: 7%;  18: 5%;  >= 19: 11%  Missing: 7%  ELSA:  <=14: 31%;  15: 27%;  16: 17%;  17: 6%;  18: 5%;  >= 19: 11%  Missing: 3% |
| **Turner 2015**  **Aim:** effect of living alone at each stage of the treatment pathway (hip or knee replacement) | England | Secondary analysis of linked administrative data; 6-month follow-up | NHS Health and Social Care Information Centre (HSCIC) for financial years 2009/10 and 2010/11 linked to  Hospital Episode Statistics | Population who had a hip or knee replacement paid for by the NHS in financial years 2009/10 and 2010/11 | Not living alone:  white 97% (N=65089), Asian/Asian British/Asian mixed 2% (N=1457), Black/Black British/Black Mixed 0.9% (N=583).  Living alone White 98% (N=24,031), Asian/Asian British/Asian mixed 1% (N=201);  Black/Black British/Black Mixed 1% (N=273). | Not living alone: mean 68.25 (SD 8.42)  Living alone: mean 73.38 (SD 8.85) | Not living alone: M N=36,333 (48.5%); living alone M N=6666 (24.5%) | NR |
| **Vlachantoni 2015**  **Aim:** to examine the relationship between demographic  and socio-economic characteristics and the receipt of support from different sources by older people who report difficulty with daily activities | England | Cross-sectional | ELSA (wave 4)  2008 | Core respondents of the ELSA dataset who are aged >=65, and had no missing information in their report of difficulty with at least one ADL, IADL or mobility. | NR | ≥65 years  Unweighted sample N:  65-69: 301  70-74: 363  75-79: 244  80-84: 181  85-89: 82  90+: 35 | Unweighted sample N:  Men: 1215 women 1922 | NR |
| **Vlachantoni 2019**  **Aim:** to identify the  patterns of unmet need for social care; focusing on the individual characteristics associated with experiencing  unmet need in relation to mobility tasks, activities of daily living (ADLs) and instrumental activities of daily living (IADLs). | England | Cross-sectional | ELSA (wave 7)  2014/15 | ≥65 years recruited to ELSA and had no missing information in their report of difficulty with at least one ADL, IADL or mobility.  N= | NR | ≥65 years  65-74: 3,154 75-84: 1,875 85+: 562 | Men: 3154/5591 (56%)  Women: 1875/5591 (34%) | None: 35%  Low: 52.9%  High: 12.2% |

^1^ [The English Longitudinal Study of Ageing (ELSA)](https://www.elsa-project.ac.uk/) recruited participants age < 50 years for ELSA wave 0 from the Health Survey of England (HSE) (1998 to 2001). The same group of respondents has been interviewed at two-yearly interviews, known as ‘waves’, to measure changes in their health, economic and social circumstances. The ELSA sample is refreshed, additional participants were added to waves 3, 4, 6, 7, 9, and 10. When a refreshment sample is drawn, households from HSE are selected for ELSA if at least one HSE interview was with an age eligible respondent who agreed to be re-contacted. Each refreshment sample is part of the cohort for subsequent waves.
