# Supplementary material for: Social and Health Conditions That Drive the Need and Demand for, Use of and Expenditure on Social Care in the UK: A Systematic Review
Source: Cochrane Evid Synth Methods. 2026 Aug 12;4(5):e70093. doi: 10.1002/cesm.70093 (PMC13489229; doi:10.1002/cesm.70093)
Supplement: Supplementary file 7 [file CESM-4-e70093-s007.docx]

Table S4 – GRADE and Summary of Findings of the social and health conditions associated with the need and demand for, use and cost of social care

**Summary of Findings 1**

| **Patients or population: defined by age, range 50 years *(>50% of the study sample aged >60 years*) to > 85 years**  **Settings: living in the community**  **Social condition: age** | | | |
| --- | --- | --- | --- |
| Outcomes | Impact | Number of  participants (studies) | Certainty of evidence (GRADE) |
| Need for care | **Uncertain** of the association with age and the need for care, two cohort studies did not find a clear association (HR 1.02, 95% CI 0.96 to 1.09, >85 years), (1) (OR 1.03, 95% CI 0.88 to 1.01, mean age 77), (2) one that increasing age was associated with less likely to have unmet need (OR 0.70, 95% CI 0.36 to 1.36, age 65 to 69 years to OR 0.24, 95% CI: 0.12 to 0.47, age 75+ years). (3) Two cross-sectional studies (mean age 66 years; and ≥ 65 years) found age was associated with being more likely to have unmet need for care with age (OR 1.01, 95% CI 1.00 to 1.02, to OR 3.00, 95% CI 1.8 to 4.99). (4) (5) | 5 studies (3 cohort and 1 cross-sectional study)  *N= 92,914*  *Follow-up for the three cohort studies 8 to 10 years*  *Unmet need measured by: mobility difficulties,* (1) *difficulties in activities of daily living,* (4) *difficulties in ADL, IADL or mobility* (2, 3, 5) | ⊕⊖⊖⊖  Very low certainty due to risk of bias from residual confounding, lack of data on post-exposure interventions; and inconsistency in study design, findings and measures of the need for care |
| Demand for care | No studies reported this outcome | *-* | - |
| **Use** of care | Age **may increase** admission to a long-term care facility (mean age 76 years, HR 1.06 per year, 95% CI 1.03–1.09) (6) and on-going care in the home or admission to a care facility (age 85+, ref 75-79, OR 2.17, 95% CI 1.55 to 3.04); (7) | *2 cohort studies*  *N=15,427*  *Follow-up 6 months to 3 years* | ⊕⊕⊖⊖  Low certainty due to risk of bias from residual confounding, lack of data on post-exposure interventions, |
| **Cost** of or expenditure on care | **Uncertain** of the association of age, one cross-sectional studies (mean age 76 years) found no association with age and social care costs. (8) | *1 cross sectional study*  *N=1,458 (452 used social care)*  *Follow-up N/A* | ⊕⊖⊖⊖  Very low certainty, due to risk of bias, high level of variation in costs, and 31% of the sample with 3 + LTCs incurred social care costs |
| **Patients or population: older population, mean age 66 to > 85 years**  **Settings: living in the community**  **Social condition: sex** | | | |
| Need for care | **Uncertain** of the association with sex.  One cohort study found that females were more likely to need care (HR 1.64, 95% CI 1.25-2.14); (1) two cross-sectional studies that males were more likely to need care (OR 1.64, 95% CI 1.44 to 1.87; (4) OR 1.47, 95% CI 1.07 to 2.02 to OR 2.00, 95% CI 1.49 to 2.67 for different measures of need); (5) two cohort studies no clear association (OR 1.35, 95% CI 0.93 to1.95; (3) OR 1.003, 95% CI 0.895 to 1.123. (2)  For trajectories of the need and use of care, females were more likely to follow a medium need/use trajectory vs low need/use OR 1.35, SE 0.1, p<0.001. (9) | *6 studies (4 cohort and 2 cross sectional studies)*  *N=97,543*  *Follow-up 6 to 10 years*  *Unmet need measured by: mobility difficulties,* (1) *difficulties in activities of daily living,* (4) *difficulties in ADL, IADL or mobility, (2)* (3) (5) and *number of functional limitations and intensity by the weekly hours of care received. (9)* | ⊕⊖⊖⊖  Very low certainty due to risk of bias from residual confounding, lack of data on post-exposure interventions, and inconsistency in study design, findings and measures |
| Demand for care | No studies reported this outcome | *-* | - |
| Use of care | **Uncertain** of the association with sex (male) and admission to long-term care (HR 0.72, 95% CI 0.53 to 0.99); (6) or of long-term residential or home care (OR 1.25, 95% CI 0.94 to 1.67); (7) a cross-sectional study found females were more likely to use paid care (OR 1.63, 95% CI 1.08 to 2.47). (10) | *3 studies (1 prospective, 1 retrospective cohort, 1 cross sectional study)*  *N=18,822*  *Follow-up for cohort studies 1 to 3 years* | ⊕⊖⊖⊖  Very low certainty due to risk of bias from residual confounding, lack of data on post-exposure interventions, and inconsistency in study design, findings and measures |
| Cost of or expenditure on care | **Uncertain** of the association with sex, a cross-sectional study found lower social care costs for males with 3+ LTCs. (8) | *1 cross-sectional study*  *N=1,458 (452 used social care)* | ⊕⊖⊖⊖  Very low certainty due to risk of bias, high level of variation in costs and 31% of the sample with 3 + LTCs incurred social care costs |
| **Patients or population: older population, mean age 66 to > 85 years**  **Settings: living in the community**  **Social condition: ethnicity** | | | |
| Need | **Uncertain** of the association with ethnicity, a cross-sectional study found no clear association with ethniciy and unmet need (OR 1.15, 95% CI 0.83 to 1.59). (4) For trajectories of the need and use of care, those in a minority ethnic group (ref white) were more likely to follow a medium need/medium intensity use of care vs low need/low intensity use of care (OR 2.02, SE 0.47, p<0.01). (9) | *2 studies (1 cohort and 1 cross-sectional study)*  *N=85,967*  *Follow-up 6 years for trajectories of need/use of care*  *Need defined by the number of functional limitations and intensity by the weekly hours of care received, (9) and difficulties in activities of daily living.* (4) | ⊕⊖⊖⊖  Very low certainty due to risk of bias from residual confounding, lack of data on post-exposure interventions, and inconsistency in study design and measures. |
| Demand | No studies reported this outcome | *-* | - |
| Use | **Uncertain** of the association with ethnicity and the use of care (Asian/British Asian OR 1.28, 95% CI 0.77 to 2.03; Black/Black British OR 1.31, 95% CI 0.79 to 2.10, Mixed ethnicity OR 1.16, 95% CI 0.77 to 1.72). (7) | *1 cohort study*  *N=13,394*  *Follow-up 12 months* | ⊕⊖⊖⊖ Very low certainty due to risk of bias from residual confounding, lack of data on post-exposure interventions and imprecision |
| Cost | No studies reported this outcome | *-* | - |
| **Patients or population: older population, mean age 66 to > 85 years**  **Settings: living in the community**  **Social condition: living alone** | | | |
| Need | **May increase** the need for care, a cohort and cross sectional study found living alone was associated with more likely to have unmet need for care (OR 1.74, 95% CI 1.08 to 2.8), (3) (range OR 3.30, 95% CI 1.71 to 6.37 for help with ADLs to OR 1.93, 95% CI 1.09 to 3.41 for help with mobility). (5)  In one cohort study the trajectories of need and use of care, married vs single people were more likely to follow a high need/high intensity care vs high need/ medium intensity care trajectory (OR 3.57, SE 0.84, p<0.001). (9) | *3 studies (2 cohort studies and 1 cross-sectional study)*  *N=11, 302*  *Follow-up 6 to 10 years*  *Need defined by difficulties in ADL, IADL or mobility,* (3) *the number of functional limitations and intensity by the weekly hours of care received, (9) and difficulties in activities of daily living.* (4) | ⊕⊕⊖⊖  Low certainty due to risk of bias from residual confounding, lack of data on post-exposure interventions, inconsistency in study design |
| Demand | No studies reported this outcome | - | - |
| Use | **Uncertain** of the association with living alone, in a population aged ≤65 years following a hip or knee replacement living alone was associated with a 0.6 percentage points (p<0.001) increase in the probability of discharge to a nursing home following after a hip or knee replacement, (11) and no clear association in a cohort study (OR 1.35, 95% CI 0.69 to 2.98); (7) | *2 studies (1 retrospective cohort, 1 secondary analysis of linked administrative data)*  *N=61,540*  *Follow-up 6 to 12 months* | ⊕⊖⊖⊖  Very low certainty due to risk of bias from residual confounding, lack of data on post-exposure interventions, and inconsistency in study design and populations, findings and measures |
| Cost of or expenditure on care | No studies reported this outcome | - | - |
| **Patients or population: older population, mean age 66 to > 85 years**  **Settings: living in the community**  **Social condition: deprivation** | | | |
| Need for care | **Uncertain** of the association with deprivation.  No clear association for education (two cohort studies), years of education HR 0.97, 95% CI 0.82 to 1.14, (1) higher education (ref no education) OR 1.05, 95% CI 0.57 to 1.93; (3) a cross-sectional study found unmet need was less likely with higher levels of education OR 0.58, 95% CI 0.49 to 0.69 for education >18 years. (4)  No clear association for housing tenure rental (ref house ownership, OR 0.69, 95% CI 0.43 to 1.1), (3) or non-housing wealth (OR 1.0, 95% CI 1.00 to 1.00). (2)  For trajectories of need and use of care, (9) housing problems were associated with a trajectory of more need and intensity of the use of care (OR 1.71, SE 0.05, p<0.001); and a higher level of wealth with lower need and intensity of use of care (OR 0.76 SE 0.08 p<0.05); in a cross-sectional study, higher wealth quintiles were associated with being less likely to need care, (ref=1^st^ quintile low wealth) range 2^nd^ quintile OR 0.74, 95% CI 0.65 to 0.85 to 5^th^ quintile OR 0.41, 95% CI 0.34 to 0.50). (4) | *5 studies (4 cohort studies and 1 cross-sectional study)*  *N=91,952*  *Follow-up 6 to 10 years*  *Unmet need measured by: mobility difficulties,* (1) *difficulties in ADL, IADL or mobility,* (3) } *difficulties in ADL, IADL or mobility* (2)*the number of functional limitations and intensity by the weekly hours of care received, (9) and difficulties in activities of daily living.* (4) | ⊕⊕⊖⊖  Very low certainty due to risk of bias from residual confounding, lack of data on post-exposure interventions, and inconsistency in study design, findings and measures of deprivation |
| Demand for care | No studies reported this outcome | - | - |
| Use of care | **Uncertain** of the association on the use of care, a retrospective cohort study found that higher wealth quintiles were associated with less likely to use long term care (ref 1^st^ quintile most deprived, range 2^nd^ quintile OR 0.72, 95% CI 0.51 to 1.03 to 4^th^/ 5^th^ quintile OR 0.37, 95% CI 0.23 to 0.56); (7) one study that calculated the concentration index (CI) found a low level of wealth was associated with the use of formal and informal care (CI -0.052, (SE 0.005), p<0.001; CI - 0.186, SE 0.007, p<0.001) (12) | *2 studies (1 retrospective cohort, 1 cross sectional study)*  *N=29, 852 Follow-up 1 to 7 years* | ⊕⊖⊖⊖  Very low certainty due to risk of bias from residual confounding, lack of data on post-exposure interventions, and inconsistency in study design, findings and measures |
| Cost of or expenditure on care | **Uncertain** of the association of deprivation and the cost of care in one cross-sectional study. (8) | *1 cross-sectional study*  *N=1,458 (452 used social care)* | ⊕⊖⊖⊖  Very low certainty due to risk of bias with high level of variation in costs and 31% of the sample with 3 + LTCs incurred social care costs |
| **Patients or population:** older population, mean age 66 to > 85 years  **Settings:** community living in England  **Social condition:** long-term conditions LTC | | | |
| Need | **Uncertain** association of LTCs and the need for care, each additional LTC was associated with being more likely to need care in one cohort (HR 1.16, 95% CI 1.07 to 1.25, p<0.001) (1) and a cross-sectional study (range for eight LTCs OR 1.52, 95%CI 1.29 to 1.80 for diabetes to OR 2.69, 95% CI 2.37 to 3.07 for arthritis) (4) and less likely in one cohort (none=ref, OR 0.56, 95% CI 0.39-0.81; (3) and cross-sectional study for help with mobility (OR 0.59, 95% CI 0.39 to 0.89). (5) | *4 studies (2 cohort and 2 cross-sectional studies)*  *N=88, 856*  *Follow-up 10 years for each cohort study* | ⊕⊖⊖⊖  Very low certainty due to risk of bias from residual confounding, lack of data on post-exposure interventions and inconsistency in study design, findings and LTCs. |
| Demand for care | No studies reported this outcome | - | - |
| Use of care | **May increase** the use of long-term adult social care, a retrospective cohort study found a mental health or neurological condition was associated with the use of long-term adult social care (OR 1.76, 95% CI 1.27 to 2.40; OR 2.11, 95% CI 1.41 to 3.06). (7) | *1 study (retrospective cohort)*  *N=13,394*  *Follow-up 12 months* | ⊕⊖⊖⊖  Very low certainty due to risk of bias and lack of data on post-exposure interventions |
| Cost of or expenditure on care | **Uncertain** association with the cost of care, a percentage point increase in the prevalence of multiple long-term conditions was associated with £8.13 (95% CI 0.24 to 16.02) per capita LA social care expenditure (2018/19 prices); (13) annual savings of £4.4 million beween 2012 to 2018 on formal social care in England, excluding care homes, for every 1% of non-frail people aged ≥75 not transitioning to frailty; (14) and no clear association with the number of LTCs. (8) | *3 cross-sectional studies*  *N=414, 455^1^*  *Follow-up N/A*  *^1^ one study the population size was 409, 551 for an area level analysis of 148 local authorities* | ⊕⊖⊖⊖  Very low certainty due to risk of bias, different years for costs and methods used, indirectness for one study that measured frailty and not a LTC, and imprecision |
| HR: hazard ratio; CI: confidence interval; OR: odds ratio; RR: relative risk  **GRADE Working Group grades of evidence** **High certainty:** we are very confident that the true effect lies close to that of the estimate of the effect. **Moderate certainty:** we are moderately confident in the effect estimate: the true effect is likely to be close to the estimate of the effect, but there is a possibility that it is substantially different. **Low certainty:** our confidence in the effect estimate is limited: the true effect may be substantially different from the estimate of the effect. **Very low certainty:** we have very little confidence in the effect estimate: the true effect is likely to be substantially different from the estimate of effect. | | | |

| **Summary of findings table 2: Population with cognitive impairment or a diagnosis of dementia** | | | |
| --- | --- | --- | --- |
| **Patients or population:** Diagnosis of dementia, and in one study dementia and Parkinson’s Disease  **Settings:** Community, England, Scotland and Wales  **Social condition:** health condition | | | |
| **Outcomes** | **Age** | **Number of  participants (Studies) ^1^** | **Certainty of evidence (GRADE)** |
| **Need for care** | No studies reported this outcome | **-** | **-** |
| **Demand for care** | No studies reported this outcome | **-** | **-** |
| **Use of care** | **May increase** the use of care. Age was associated with an increase in care home admission (OR 1.04, 95% CI 1.02-1.06) (15) and a reduction in the use of care for those ≤ 80 years, RR 0.35, 95% CI 0.15 to 0.82 for 65 to 69 years, to RR 0.50, 95% CI 0.29 to 0.89 for 75 to 79 years. (16) | *2 cohort studies*  *N=6,470 ^1^*  *Follow-up 6 months to* *2 years* | ⊕⊕⊖⊖  Low certainty due to risk of bias from residual confounding, missing data and lack of data on post-exposure interventions and different study designs |
| **Cost or expenditure on care** | **Uncertain** association with cost, an additional year of age (sample mean 76 years) was associated with a 0.8% (95% CI 1.002 to 1.015) increase in the rate of weekly costs of paid and unpaid care for a population with Parkinson’s Disease and dementia 2018/19 (17) there was no clear association for the cost of informal and paid care (18) or care home admission. (15) | *3 studies (2 cohort studies and 1 cross-sectional study)*  *N=4,360; in one study ^1^ 361/3075 provided cost data*  *Follow-up 6 months to 2 years* | ⊕⊖⊖⊖  Very low certainty due to risk of bias from residual confounding, lack of data on post-exposure interventions, missing data, and imprecision |
|  | **Male/female** |  |  |
| **Need** | No studies reported this outcome | *-* | - |
| **Demand** | No studies reported this outcome | *-* | - |
| **Use** | **Uncertain** association with male/female and care home admission in a population with AD (OR 1.1, 95%I 0.85 1.44) (15), and mild to moderate dementia (RR 0.63, 0.39 to 1.01). (16) | *2 studies (2 cohort studies) ^1^*  *N=6,475*  *Follow-up 6 months to 2 years* | ⊕⊖⊖⊖  Very low certainty due to risk of bias from residual confounding, lack of data on post-exposure interventions, missing data, and imprecision |
| **Cost** | **Uncertain** association with male/female and a care home admission in a population with AD (Exp (b) 0.99, 95% CI 0.86 to 1.14) (15) and a cross-sectional study for a population with dementia and Parkinson’s disease (Exp (b) 1.070, 95% CI 0.945, 1.211). (17) | *2 studies*  *N=1,271; in one study ^1^ 361/3075 provided cost data*  *Follow-up 6 months to 2 years* | ⊕⊖⊖⊖  Very low certainty due to risk of bias from residual confounding, lack of data on post-exposure interventions, missing data, and imprecision |
|  | **Ethnicity** |  |  |
| **Need** | No studies reported this outcome | *-* | - |
| **Demand** | No studies reported this outcome | *-* | - |
| **Use** | **Uncertain** association in one cohort study, ethnicity was associated with being less likely to use care (ref: white) for Caribbean/African and Mixed/unknown (OR 0.57, 95% CI 0.38 to 0.86; OR 0.30, 95% CI 0.12 to 0.74), and no clear association for East/South Asian (OR 0.57, 95% CI 0.24 to 1.34). (15) | *1 cohort study ^1^*  *N=5,624*  *Follow-up 6 months* | ⊕⊖⊖⊖  Very low certainty due to risk of bias from residual confounding, lack of data on post-exposure interventions, missing data, and imprecision |
| **Cost** | **Uncertain** association for ethnicity and cost of care. (15) | *1 cohort study ^1^*  *N=361*  *Follow-up 6 months* | ⊕⊖⊖⊖  Very low certainty due to risk of bias from residual confounding, lack of data on post-exposure interventions, missing data, and imprecision |
|  | **Living alone** |  |  |
| **Need** | No studies reported this outcome | *-* | - |
| **Demand** | No studies reported this outcome | *-* | - |
| **Use** | **Uncertain** association for living alone and care home admission (OR 1.29, 95% CI 0.99 to 1.67). (15) | *1 cohort study ^1^*  *N=5,624*  *Follow-up 6 months* | ⊕⊖⊖⊖  Very low certainty due to risk of bias from residual confounding, lack of data on post-exposure interventions, missing data, and imprecision |
| **Cost** | **Uncertain** association with living alone and the cost of social care with an unclear association for a change in the rate of mean weekly service costs, (17) and increased costs from admission to a care home. (15) | *2 cohort studies*  *N=1,207 ^1^*  *Follow-up 6 months to 2 years* | ⊕⊖⊖⊖  Very low certainty due to risk of bias from residual confounding, lack of data on post-exposure interventions, inconsistency, missing data, and imprecision |
|  | **Deprivation** |  |  |
| **Need** | No studies reported this outcome | *-* | - |
| **Demand** | No studies reported this outcome | *-* | - |
| **Use** | **May increase** the likelihood of care home admission from minor and significant problems with living conditions ((OR 1.59, 95% CI 1.19-2.12; OR 1.65, 95% CI 1.18-2.32). (15) | *1 cohort study ^1^*  *N=5,624*  *Follow-up 6 months* | ⊕⊕⊖⊖  Low certainty due to risk of bias from residual confounding, lack of data on post-exposure interventions, missing data, and imprecision |
| **Cost** | **Uncertain** association for ethnicity and cost of care. (15) | *1 cohort study ^1^*  *N=361*  *Follow-up 6 months* | ⊕⊖⊖⊖  Very low certainty due to risk of bias from residual confounding, lack of data on post-exposure interventions, missing data, and imprecision |
|  | **Health indicators** |  |  |
| **Need** | No studies reported this outcome | *-* | - |
| **Demand** | No studies reported this outcome | *-* | - |
| **Use** | **May increase** the probability of a care home admission if a prior admission in the previous 12 months to a hospital or mental health facility (OR 1.54, 95% CI 1.22-1.94; OR 2.59, 95% CI 1.42-4.74). (15)  **May increase** the probability of a care home admission if minor or significant agitation (OR 1.41, 95% CI 1.05 to 1.89; OR 1.98, 95% CI 1.45 to 2.70). (15)  **May increase** the probability of institutionalisation for mild or moderately severe/severe dementia (range: 9.0, 95% CI: 5.6 to 14.3 to 28.0, 95% CI: 21.0 to 36.7), (19) for Parkinson’s disease dementia/ dementia with Lewy bodies (RR 3.20, 95% CI 1.43 to 7.77), (16) and with cognitive impairment (Mini-Mental State Examination Score <26 or a diagnosis of dementia) was associated with self-reported use of social care in the 4 weeks prior to baseline OR 1.90 95% CI 1.28 to 2.83, p = 0.002.(18) | *4 studies (3 cohort studies ^1^ and 1 cross-sectional study)*  *N=10,154*  *Follow-up 6 months to 4 years* | ⊕⊕⊖⊖  Low certainty due to risk of bias from residual confounding, lack of data on post-exposure interventions, and self-reported use of social care in the last 4 weeks |
|  |  |  |  |
| **Cost of or expenditure on** |  |  |  |
| OR: odds ratio; RR: relative risk; CI: confidence interval  **GRADE Working Group grades of evidence** **High certainty:** we are very confident that the true effect lies close to that of the estimate of the effect. **Moderate certainty:** we are moderately confident in the effect estimate: the true effect is likely to be close to the estimate of the effect, but there is a possibility that it is substantially different. **Low certainty:** our confidence in the effect estimate is limited: the true effect may be substantially different from the estimate of the effect. **Very low certainty:** we have very little confidence in the effect estimate: the true effect is likely to be substantially different from the estimate of effect. | | | |

^1^ *Knapp 2016: N= 3,075 individuals who had 5,912 eligible 6 month windows, missing data for 266, data from N= 5,624 eligible windows contributed to the analysis; N=361/5624 contributed cost data.* (15)

NB: all but one study^19^ adjusted for confounding factors

**References**

1.	Davies LE, Mercer SW, Brittain K, Jagger C, Robinson L, Kingston A. The association between multimorbidity and mobility disability-free life expectancy in adults aged 85 years and over: A modelling study in the Newcastle 85+ cohort. PLoS Med. 2022;19(11):e1004130.

2.	Iparraguirre JL. Reductions in local government spending on community-based social care and unmet social care needs of older people in England. J Econ Ageing. 2020;17:100126.

3.	Dunatchik A, Icardi R, Blake M. Predicting Unmet Need for Social Care. J Long-Term Care. 2019:194–205.

4.	Gousia K, Towers AM. Unmet social care needs of people living with and beyond cancer: prevalence and predictors from an English longitudinal survey. Psychooncology. 2021;30(6):874-81.

5.	Vlachantoni A. Unmet need for social care among older people. Ageing Soc. 2019;39(4):657-84.

6.	Burton JK, Papworth R, Haig C, McCowan C, Ford I, Stott DJ, et al. Statin Use is Not Associated with Future Long-Term Care Admission: Extended Follow-Up of Two Randomised Controlled Trials. Drugs Aging. 2018;35(7):657-63.

7.	Nakubulwa M, Junghans C, Novov V, Lyons-Amos C, Lovett D, Majeed A, et al. Factors associated with accessing long-term adult social care in people aged 75 and over: a retrospective cohort study. Age and ageing. 2022;51(3).

8.	Kasteridis P, Street A, Dolman M, Gallier L, Hudson K, Martin J, et al. Who would most benefit from improved integrated care? Implementing an analytical strategy in South Somerset. Int J Integr Care. 2015;15:e001.

9.	Hu B, Cartagena-Farias J, Brimblecombe N. Functional disability and utilisation of long-term care in the older population in England: a dual trajectory analysis. Eur J Ageing. 2022;19(4):1363-73.

10.	Vlachantoni A, Shaw RJ, Evandrou M, Falkingham J. The determinants of receiving social care in later life in England. Ageing Soc. 2015;35:321-45.

11.	Turner AJ, Nikolova S, Sutton M. The effect of living alone on the costs and benefits of surgery amongst older people. Social science & medicine (1982). 2016;150:95-103.

12.	Hu B, Read S, Wittenberg R, Brimblecombe N, Rodrigues R, Banerjee S, et al. Socioeconomic inequality of long-term care for older people with and without dementia in England. Ageing Soc. 2024;44(7):1597-617.

13.	Chukwusa E, Font-Gilabert P, Manthorpe J, Healey A. The association between social care expenditure and multiple-long term conditions: A population-based area-level analysis. J Comorb Multimorb. 2023;13:26335565231208994.

14.	Nikolova S, Heaven A, Hulme C, West R, Pendleton N, Humphrey S, et al. Social care costs for community-dwelling older people living with frailty. Health & social care in the community. 2022;30(3):e804-e11.

15.	Knapp M, Chua KC, Broadbent M, Chang CK, Fernandez JL, Milea D, et al. Predictors of care home and hospital admissions and their costs for older people with Alzheimer's disease: Findings from a large London case register. BMJ Open. 2016;6(11).

16.	Sabatini S, Martyr A, Gamble LD, Matthews FE, Thom JM, Henderson C, et al. Identifying predictors of transition to a care home for people with dementia: findings from the IDEAL programme. Aging Ment Health. 2024:1-9.

17.	Henderson C, Knapp M, Martyr A, Gamble LD, Nelis SM, Quinn C, et al. The Use and Costs of Paid and Unpaid Care for People with Dementia: Longitudinal Findings from the IDEAL Cohort. J Alzheimers Dis. 2022;86(1):135-53.

18.	MacLeod CA, Bu F, Rutherford AC, Phillips J, Woods R. Cognitive impairment negatively impacts allied health service uptake: Investigating the association between health and service use. SSM - population health. 2021;13:100720.

19.	Lenox-Smith A, Reed C, Lebrec J, Belger M, Jones RW. Resource utilisation, costs and clinical outcomes in non-institutionalised patients with Alzheimer’s disease: 18-month UK results from the GERAS observational study. BMC Geriatr. 2016;16:195-.
